# Integration of deep transcriptome and proteome analyses reveals the components of alkaloid metabolism in opium poppy cell cultures

**DOI:** 10.1186/1471-2229-10-252

**Published:** 2010-11-18

**Authors:** Isabel Desgagné-Penix, Morgan F Khan, David C Schriemer, Dustin Cram, Jacek Nowak, Peter J Facchini

**Affiliations:** 1 Department of Biological Sciences, University of Calgary, Calgary, Alberta, T2N 1N4, Canada; 2 Department of Biochemistry and Molecular Biology, University of Calgary, Calgary, Alberta, T2N 4N1, Canada; 3 National Research Council-Plant Biotechnology Institute, Saskatoon, Saskatchewan, S7N 0W9, Canada

## Abstract

**Background:**

*Papaver somniferum *(opium poppy) is the source for several pharmaceutical benzylisoquinoline alkaloids including morphine, the codeine and sanguinarine. In response to treatment with a fungal elicitor, the biosynthesis and accumulation of sanguinarine is induced along with other plant defense responses in opium poppy cell cultures. The transcriptional induction of alkaloid metabolism in cultured cells provides an opportunity to identify components of this process via the integration of deep transcriptome and proteome databases generated using next-generation technologies.

**Results:**

A cDNA library was prepared for opium poppy cell cultures treated with a fungal elicitor for 10 h. Using 454 GS-FLX Titanium pyrosequencing, 427,369 expressed sequence tags (ESTs) with an average length of 462 bp were generated. Assembly of these sequences yielded 93,723 unigenes, of which 23,753 were assigned Gene Ontology annotations. Transcripts encoding all known sanguinarine biosynthetic enzymes were identified in the EST database, 5 of which were represented among the 50 most abundant transcripts. Liquid chromatography-tandem mass spectrometry (LC-MS/MS) of total protein extracts from cell cultures treated with a fungal elicitor for 50 h facilitated the identification of 1,004 proteins. Proteins were fractionated by one-dimensional SDS-PAGE and digested with trypsin prior to LC-MS/MS analysis. Query of an opium poppy-specific EST database substantially enhanced peptide identification. Eight out of 10 known sanguinarine biosynthetic enzymes and many relevant primary metabolic enzymes were represented in the peptide database.

**Conclusions:**

The integration of deep transcriptome and proteome analyses provides an effective platform to catalogue the components of secondary metabolism, and to identify genes encoding uncharacterized enzymes. The establishment of corresponding transcript and protein databases generated by next-generation technologies in a system with a well-defined metabolite profile facilitates an improved linkage between genes, enzymes, and pathway components. The proteome database represents the most relevant alkaloid-producing enzymes, compared with the much deeper and more complete transcriptome library. The transcript database contained full-length mRNAs encoding most alkaloid biosynthetic enzymes, which is a key requirement for the functional characterization of novel gene candidates.

## Background

Opium poppy (*Papaver somniferum*) remains our most important source for several pharmaceutical benzylisoquinoline alkaloids (BIAs) including the narcotic analgesic morphine, the anti-tussive drug codeine, the vasodilator papaverine and the antimicrobial agent sanguinarine. In opium poppy plants, most BIAs (e.g., morphine, codeine and papaverine) occur in the cytoplasm (i.e. latex) of specialized cells, known as laticifers, that are associated with the phloem in all organs. Although latex and, thus, most BIAs are most abundant on shoot organs of opium poppy, the antimicrobial alkaloid sanguinarine accumulates constitutively in roots possibly in association with cell types other than laticifers. Although opium poppy cell cultures do not produce BIAs constitutively, the biosynthesis of sanguinarine is induced in response to treatment of the cells with a fungal elicitor. As such, opium poppy cell cultures provide an effective model system to investigate the inducible regulation of BIA metabolism and other plant defense pathways. The induction of sanguinarine biosynthesis and supporting metabolism in elicitor-treated opium poppy cell cultures has been characterized using a variety of technologies including EST and species-specific microarray analyses to analyze the transcriptome [1], LC-MS/MS to survey the proteome [2], and Fourier-transform ion-cyclotron resonance-mass spectrometry (FT-ICR-MS) to profile the metabolome [1,3]. Although these studies provided valuable insights into the response of opium poppy cell cultures to fungal elicitor treatment, the technologies used to generate the various databases were limited in terms of the depth of penetration into the transcriptome, proteome and metabolome of the cultured cells. For example, LC-MS/MS peptide analysis of 340 spots isolated by two-dimensional SDS-PAGE led to the identification of 219 proteins using a combination of public and species-specific sequence databases. More extensive genomics resources for opium poppy would improve the downstream identification and discovery of enzymes involved in alkaloid biosynthesis. New sequencing technologies such as 454 pyrosequencing, and advances in LC-MS/MS-based proteomics and bioinformatics, will expand the application of genomics methodologies to a vast array of non-model plants that produce interesting and valuable metabolites.

**Figure 1:**
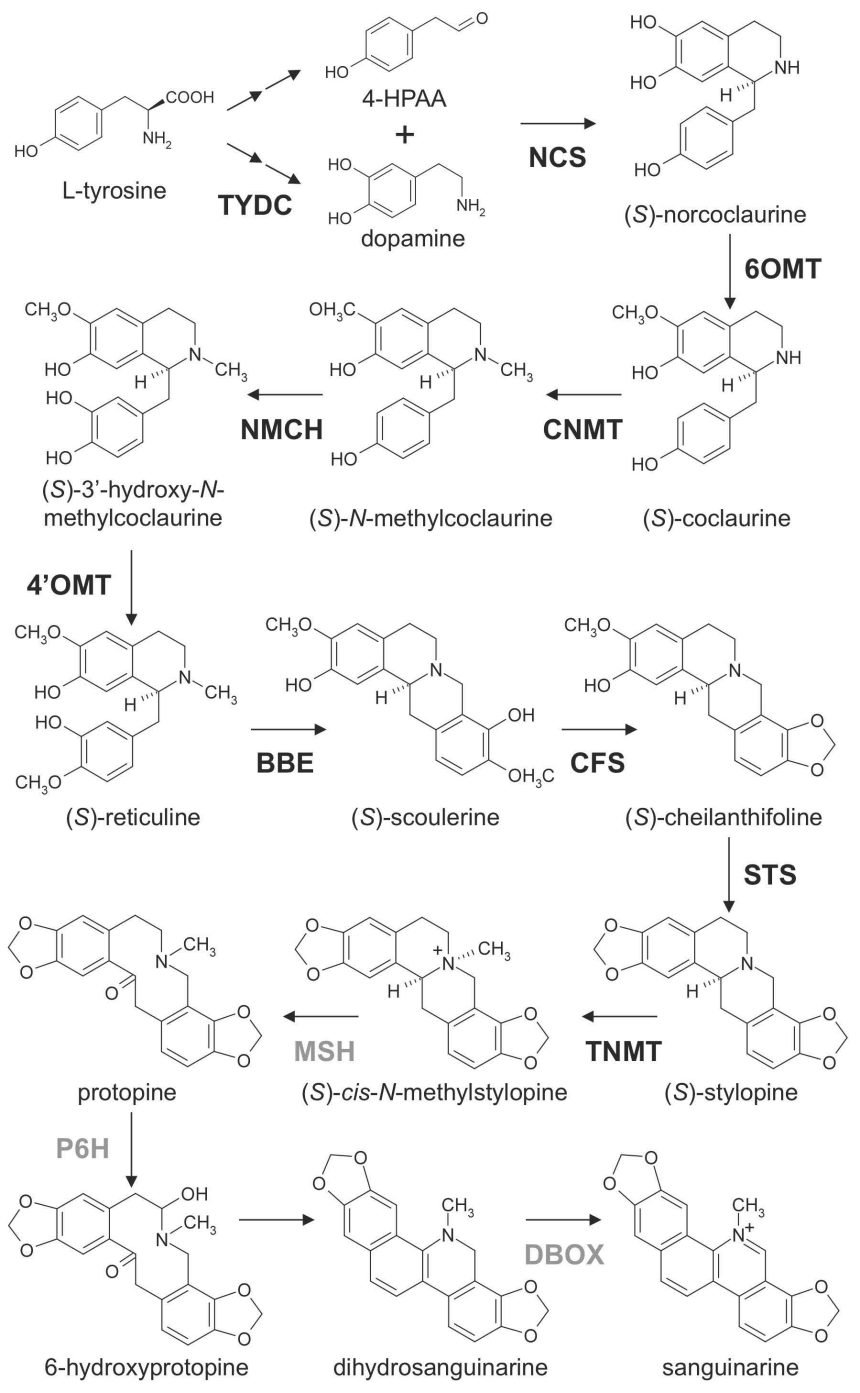
**Biosynthetic pathway from tyrosine to sanguinarine**. Biosynthesis of sanguinarine from tyrosine. Enzymes for which cognate cDNAs have been isolated are shown in black. Abbreviations: TYDC, tyrosine/dopa decarboxylase; NCS, norcoclaurine synthase; 6OMT, (S)-norcoclaurine 6-O-methyltransferase; CNMT, (S)-coclaurine N-methyltransferase; NMCH, (S)-N-methylcoclaurine 3'-hydroxylase; 4'OMT, (S)-3'-hdroxy-N-methylcoclaurine 4'-O-methyltransferase; BBE, berberine bridge enzyme; CheSyn, cheilanthifoline synthase; StySyn, stylopine synthase; TNMT, tetrahydroprotoberberine N-methyltransferase; MSH, methylstylopine hydroxylase; P6 H, protopine 6-hydroxylase; DBOX, dihydrobenzophenanthridine oxidase. StySyn and CheSyn cDNAs were functionally characterized in plant species other than opium poppy.

The biosynthesis of BIAs in opium poppy starts with the condensation of two tyrosine derivatives, dopamine and 4-hydroxyphenylacetaldehyde (4-HPAA), by norcoclaurine synthase (NCS) to yield (S)-norcoclaurine (Figure 1) [4-6]. The formation of dopamine involves the decarboxylation of tyrosine and/or dihydrophenylalanine (DOPA) by tyrosine/DOPA decarboxylase (TYDC) [7]. (S)-Norcoclaurine is then methylated by the norcoclaurine 6-O-methyltransferase (6OMT) and coclaurine N-methyltransferase (CNMT) to yield (S)-methylcoclaurine [8-11]. The P450-dependent monooxygenase (S)-N-methylcoclaurine-3'-hydroxylase (NMCH or CYP80B3) catalyzes the 3'-hydroxylation of (S)-N-methylcoclaurine prior to the formation of (S)-reticuline by the 3'-hydroxy-N-methylcoclaurine 4'-O-methyltransferase (4'OMT) [8,11-14]. (S)-Reticuline is the central intermediate in the biosynthesis of most BIA structural types including morphinans (e.g., morphine), benzophenanthridines (e.g., sanguinarine) and substituted benzylisoquinolines (e.g., laudanine and papaverine) (Additional File 1).

The berberine bridge enzyme (BBE) converts (S)-reticuline to (S)-scoulerine as the first committed step in sanguinarine biosynthesis (Figure 1) [13,15-17]. Two P450-dependent enzymes, cheilanthifoline synthase (CheSyn) and the stylopine synthase (StySyn), catalyze the formation of two methylenedioxy bridges and yield (S)-stylopine [18,19]. Subsequently, tetrahydroprotoberberine cis-N-methyltransferase (TNMT) converts (S)-stylopine to (S)-cis-N-methylstylopine [20]. Two additional P450-dependent enzymes, N-methylstylopine 14-hydroxylase (MSH) [21] and protopine 6-hydroxylase (P6H) are responsible for the conversion of (S)-cis-N-methylstylopine to 6-hydroxyprotopine, which spontaneously rearranges to yield dihydrosanguinarine [21,22]. Finally, dihydrosanguinarine is oxidized to sanguinarine by the oxygen-dependent oxidoreductase dihydrobenzophenanthridine oxidase (DBOX) [23,24]. The biosynthesis of morphine involves the epimerization of (S)-reticuline to (R)-reticuline followed by a series of C-C phenol coupling, two reductions, O-acetylation and two O-demethylations [25-29]. Reticuline 7-O-methyltransferase (7OMT) converts (S)-reticuline to (S)-laudanine [9], whereas norreticuline 7-O-methyltransferase (N7OMT) yields norlaudanine from norreticuline [30] (Additional File 1). Cognate cDNAs have been reported for all of the aforementioned enzymes with the exception of MSH, P6H and DBOX.

The standard approach to establish genomics resources for non-model plant species involves the random generation of expressed sequence tags (ESTs) from a cDNA phagemid library using dideoxy chain-termination (Sanger) sequencing technology. Next-generation technologies, such as 454 pyrosequencing, have the potential to dramatically increase the availability of sequence data [31,32]. The redundancy and depth of coverage of 454 pyrosequencing also provides and unbiased representation of transcript abundance, which is useful for relative gene expression analysis especially in non-model plants that lack complete genome sequence information [33,34]. However, despite the fundamental importance of transcriptome analysis in genomics-based research, the frequent incongruity between steady-state protein levels and the abundance of cognate gene transcripts [35] is crucial to the interpretation of relative gene expression profiles in the context of systems biology or gene discovery applications. Complementary analysis of the most abundant proteins combined with a comprehensive transcriptome database provides an important validation tool for the relative importance of gene transcripts within a given cell, tissue or organ. Transcript and protein sequence databases have recently been reported for opium poppy cell cultures using Sanger sequencing of randomly selected cDNAs and first-generation LC-MS/MS analysis of proteins isolated by two-dimensional SDS-PAGE [1,2]. In terms of the components of sanguinarine metabolism, transcripts corresponding to all known biosynthetic genes were present in the EST database, although some were represented by relatively few sequence reads. Combined with a low-throughput two-dimensional sampling approach the limited availability of sequence data more severely hampered the identification of known sanguinarine biosynthetic enzymes using LC-MS/MS analysis, which yielded only 6OMT [2].

We report the integration of next-generation 454 pyrosequencing and advanced LC-MS/MS analysis to establish a deep survey of the transcriptome and proteome of opium poppy cell cultures in response to treatment with a fungal elicitor. The effectiveness of 454 pyrosequencing is compared to standard Sanger sequencing with respect to depth of penetration into the transcriptome of elicitor-treated opium poppy cells. Use of the enhanced EST database for the identification of corresponding peptide sequences obtained by one-dimensional SDS-PAGE and LC-MS/MS peptide analysis facilitated the identification of more than 1,000 peptides and polypeptides. Most of the known enzymes involved in sanguinarine biosynthesis and many components of primary metabolic pathways that support alkaloid production are present in the protein database. Several candidate proteins and transcripts that potentially represent novel biosynthetic enzymes involved in the biosynthesis of sanguinarine and other BIAs are also represented.

## Results

### Induction of sanguinarine accumulation in opium poppy cell cultures

The content of reticuline, protopine and sanguinarine were determined at several time points after elicitor treatment of opium poppy cell cultures to facilitate a correlation of the occurrence of specific transcript and proteins with the abundance of intermediate and end-product alkaloids (Additional File 2). Reticuline was detected at low levels over the entire time course, but the levels of this central pathway intermediate decreased beginning 50 h after elicitor treatment. Protopine and sanguinarine were not detected at early time points after elicitor treatment, but both accumulated later in the time course. Sanguinarine levels began to increase rapidly between 10 and 50 h after the addition of elicitor, and reach a concentration of more than 5 μg/g fresh-weight of cells by the end of the 100-h time course. At 100 h post-elicitation, the level of sanguinarine was 40-fold greater than that of reticuline or protopine. An inverse correlation between the levels of reticuline and protopine/sanguinarine was apparent over the duration of the time course.

### Properties of the transcript database generated by 454 pyrosequencing

A total of 427 369 high-quality expressed sequence tags (ESTs) with an average read length of 462 bp were generated by 454 pyrosequencing of half a plate using GS FLX Titanium system (Table 1). The assembly of overlapping sequences yielded a total of 93,723 unigenes, of which 37,329 (39.8%) were composed of two or more contiguous ESTs (i.e. contigs), whereas 56,394 (60.2%) consisted of only a single unique sequence (i.e. singletons) (Table 1). BLASTx analysis showed that these unigenes could be classified into two groups. The first group contained 73,496 (78.4%) unigenes that displayed similarity to known genes (BLASTx expectation value of e < 10^-5^). The second group consisted of 20,227 (21.6%) unigenes that showed no similarity with any gene in the public UniProt database. Unigenes in the latter group could represent previously uncharacterized or unknown genes, sequences specific to opium poppy, or gene fragments that are too short to annotate.

**Table 1:** Summary of the expressed sequence tag databases for elicitor-treated opium poppy cell cultures obtained using 454 GS-FLX Titanium pyrosequencing.

Feature	Number
Total number of EST sequences clustered*	427 369
Average length of EST sequences (bp)	462
Number of contigs	37 329
Number of singletons	56 394
Total number of unigenes**	93 723
Number of unigenes blasted with no hits***	20 27

*After removal of sub-standard sequence

**Sum of contigs and singletons

***BLASTx search of the UniProt Plants v14.8 database (e-value cutoff of 10^-5^)

**Figure 2:**
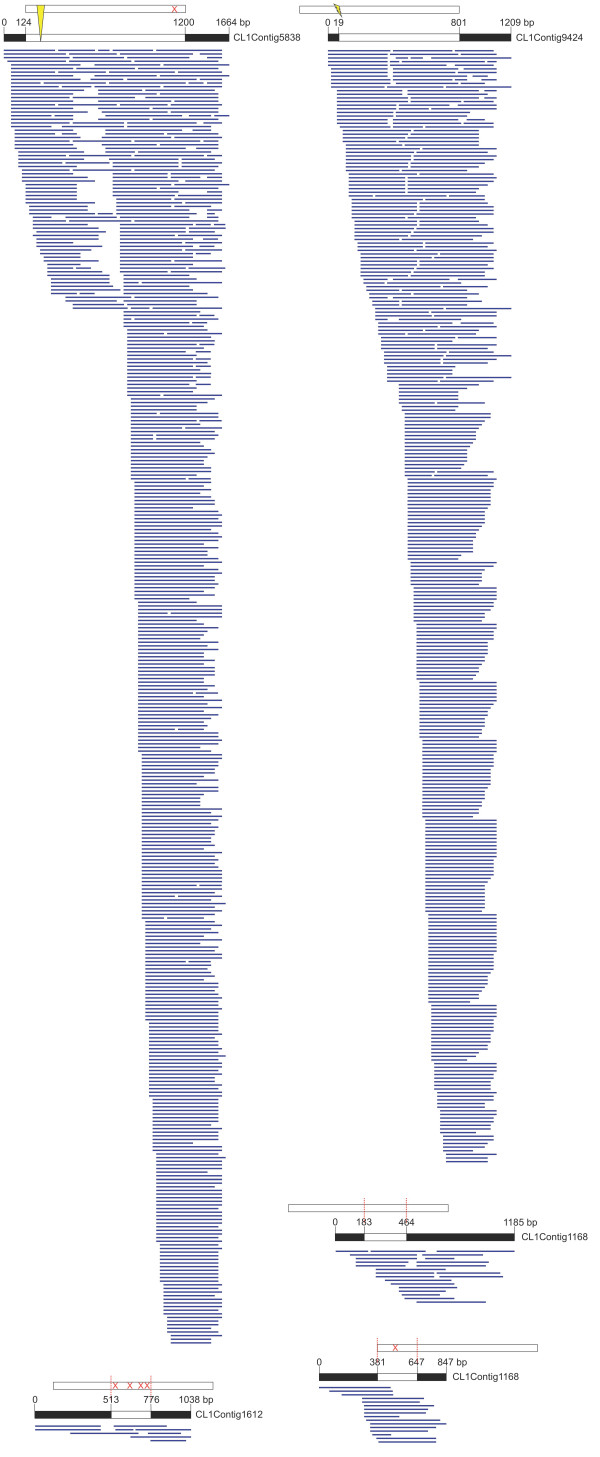
**Clustering of 454 pyrosequencing data annotated as TNMT**. Various examples representing assembly of ESTs for TNMT annotated unigenes found in the 454 database. The upper bar corresponds to the translated TNMT protein (Accession number Q108P1_PAPSO). The lower bar represents the unigene found in the 454 database and labelled with the contig number. The white region reflects the TNMT open reading frame. See Additional File 4 for a summary of unigenes shown in this figure.

The discovery rate of new unigenes reach saturation after approximately 250,000 pyrosequencing reads suggesting that near-complete representation of the elicitor-treated opium poppy cell culture transcriptome was achieved (Additional File 3A). The majority of unigenes were between 200 and 600 bp in length and although the percentage of unigenes longer than 600 bp was considerably lower, 1,716 contigs showed greater than 90% coverage of predicted open reading frames among known genes identified by BLASTx analysis (Additional File 3B and 3C). Errors in sequencing and the assembly of contigs could have resulted in the apparently low representation of full-length transcripts. The possibility that greater overall sequence coverage was present in the database is supported by the frequent occurrence of several independent unigenes encoding the same gene product. For example, opium poppy TNMT was represented by 19 unigenes (Figure 2 and Additional File 4). The most abundant of these unigenes (i.e. Contig1) was assembled from 534 independent 454 pyrosequencing reads and, although it covered the entire open reading frame encoding TNMT, the predicted protein showed only 96% amino acid identity compared with the published sequence (Figure 2) [20]. Two other independent unigenes (i.e. Contig2 and Contig3) displayed 100% amino acid sequence identity with respect to the published sequence, but did not show complete open reading frame coverage. The multiple contigs likely represent different TNMT isoforms of independent genes expressed in elicitor-treated opium poppy cell cultures. Alternatively, the large number of unigenes might reflect sequencing and/or assembly errors.

An anticipated feature of the EST database is the substantially greater coverage of the 3'-ends of several contigs resulting from (1) the proportionately larger number of partial cDNAs in the library and (2) the relative inefficiency of shearing short cDNAs prior to 454 pyrosequencing (Figure 2). In most cases and as demonstrated for TNMT (Figure 2), relatively few unigenes were assembled from the majority of sequence reads corresponding to the same gene product. Taking this into account, the gene density of opium poppy can be estimated by only dividing the number of contigs (i.e. 37,329) by the size of the opium poppy genome, estimated at 3,724 Mbp [36], which yields a gene density of approximately 10 genes per Mbp.

### Deep transcriptome analysis of elicitor-treated opium poppy cell cultures

The large number of reads generated by 454 pyrosequencing allows a robust comparison of the relative expression of different genes. Moreover, the saturation of newly discovered unigenes after the analysis of approximately 250,000 ESTs (Additional File 2A) suggests near-complete coverage of the elicitor-treated opium poppy cell culture transcriptome. However, accurate identification of each EST is essential to facilitate the quantification of all reads corresponding to selected genes. The unigenes were mapped to UniProt version 14.8 (minus genomic sequences from *Vitis vinifera*, which have not been annotated) using BLASTx analysis with an expectation highly expressed value of e < 10^-5 ^included in the high-scoring segment pair. The 50 most abundant unigenes represented in the transcriptome accounted for approximately 9% of the transcriptome (Table 2). The single most abundant transcript was sampled 3,165 times and annotated as a senescence-associated protein, a putative cytochrome P450 monooxygenase, from pea. The remaining top-50 most highly represented transcripts were sampled between 1,579 and 409 times and encode proteins involved in metabolism, defense, signaling, transport and cellular structure (Table 2). Transcripts encoding several enzymes involved in the biosynthesis of S-adenosylmethionine (SAM) such as SAM synthetase, S-adenosylhomocyteinase, and methionine synthase were highly represented in the database. Abundant transcripts encoding defense-response proteins encoded chitinase, β-lactamase, polyphenol oxidase, xyloglucanase inhibitor, peroxidase, and pathogenesis-related (PR) proteins. Transcripts encoding components of the proteosome and polyubiquitin, along with several housekeeping proteins including an elongation factor, ubiquitin and ribosomal proteins were also abundant. Most importantly, six of the top-50 most abundant transcripts encoded known enzymes involved in sanguinarine biosynthesis: NCS, CNMT, BBE, StySyn and two putative TNMT isoforms (Table 2).

**Table 2:** The fifty most abundant unigenes in the opium poppy 454 G S-F L X Titanium pyrosequencing database.

Rank	Number of reads	Annotation	Protein score*	Plant species	Accession number
1	3165	Senescence-associated protein	859	Pisum sativum	Q9AVH2_PEA
2	1579	S-Adenosylmethionine synthetase	609	Nicotiana suaveolens	Q069K3_9SOLA
3	1579	S-Adenosylmethionine synthetase	1846	Solanum tuberosum	METK2_SOLTU
4	1323	Multiprotein bridging factor	600	Solanum tuberosum	Q9LL86_SOLTU
5	1220	Heat shock protein	2973	Cucurbita maxima	Q8GSN4_CUCMA
6	1176	Chitinase, class IV	903	Nepenthes alata	A9ZMK1_NEPAL
7	1154	Berberine bridge enzyme	2698	Papaver somniferum	RETO_PAPSO
8	1120	60 S ribosomal protein L6	827	Mesembryanthemum crystallinum	RL6_MESCR
9	1106	Elongation factor 1α	2227	Lilium longiflorum	Q9SPA1_LILLO
10	1009	Beta lactamase	1388	Zea mays	Q285M4_MAIZE
11	978	Heat shock protein 90	2846	Nicotiana tabacum	Q14TB1_TOBAC
12	950	40 S ribosomal protein S9	862	Solanum demissum	Q60CZ2_SOLDE
13	863	Methionine synthase	3422	Carica papaya	A6YGE7_CARPA
14	855	Coclaurine *N*-methyltransferase	1351	Papaver somniferum	Q7XB08_PAPSO
15	847	Polyphenol oxidase	1498	Malus domestica	PPO_MALDO
16	844	Fructose-bisphosphate aldolase	1644	Solanum tuberosum	Q2PYX3_SOLTU
17	841	Nodulin protein	457	Oryza sativa subsp japonica	Q5VRN2_ORYSJ
18	807	Proteasome component protein	69	Medicago truncatula	A2Q5C5_MEDTR
19	757	Nectarin IV/xyloglucanase inhibitor	1484	Nicotiana langsdorffii × N. sanderae	Q3KU27_NICLS
20	735	Cellulose synthase	1669	Zea mays	B6SW15_MAIZE
21	729	Luminal-binding protein 5	2728	Nicotiana tabacum	BIP5_TOBAC
22	717	Elongation factor 1α	2231	Prunus persica	B6V864_PRUPE
23	686	Uncharacterized protein	864	Arabidopsis thaliana	Q9LZN8_ARATH
24	680	Peroxidase	1154	Medicago truncatula	A4UN76_MEDTR
25	652	Adenosylhomocysteinase	2300	Medicago sativa	SAHH_MEDSA
26	647	Pathogenesis-related protein	383	Solanum lycopersicum	Q53U35_SOLLC
27	630	Norcoclaurine synthase 1	1114	Papaver somniferum	Q4QTJ2_PAPSO
28	621	Sterol dehydrogenase	1133	Arabidopsis thaliana	O22856_ARATH
29	572	ADP ribosylation factor	936	Daucus carota	Q38JU3_DAUCA
30	569	Pathogenesis-related protein	376	Solanum lycopersicum	Q53U35_SOLLC
31	534	Tetrahydroprotoberberine N-methyltransferase	1780	P apaver somniferum	Q108P1_PAPSO
32	528	Polyubiquitin	740	Euphorbia esula	Q9M5X0_EUPES
33	507	ABC transporter	1930	Oryza sativa subsp japonica	Q84ZB2_ORYSJ
34	506	Polyphenol oxidase	1866	Annona cherimola	A0A168_ANNCH
35	479	S-Adenosylmethionine synthetase	1928	Vitis vinifera	METK2_VITVI
36	468	Lipid transfer protein	239	Oryza sativa subsp japonica	Q6L4H1_ORYSJ
37	465	Glycoprotein	515	Daucus carota	Q05929_DAUCA
38	463	β-D-glucosidase	2574	Gossypium hirsutum	Q7XAS3_GOSHI
39	456	Cysteine proteinase	1690	Elaeis guineensis var. tenera	A6N8F8_ELAGV
40	449	Ripening-regulated protein	857	Oryza sativa subsp japonica	Q6ZJI2_ORYSJ
41	448	Stylopine synthase	1996	Eschscholzia californica	Q50LH3_ESCCA
42	436	Glycoprotein	506	Daucus carota	Q05929_DAUCA
43	435	Calreticulin	1738	Berberis stolonifera	CALR_BERST
44	434	FAD-dependent oxidoreductase	1206	Arabidopsis thaliana	O64743_ARATH
45	434	Xyloglucanase inhibitor	1485	Solanum tuberosum	Q7XJE7_SOLTU
46	434	Uncharacterized protein	1245	Arabidopsis thaliana	Q8VZ33_ARATH
47	422	Tetrahydroprotoberberine *N*- methyltransferase	1413	Papaver somniferum	Q108P1_PAPSO
48	419	ATPase, AAA-type	1486	Arabidopsis thaliana	Q9FKM3_ARATH
49	415	Spindle disassembly protein	2459	Nicotiana tabacum	Q1G0Z1_TOBAC
50	409	Pathogenesis-related protein	383	Solanum lycopersicum	Q53U35_SOLLC

* Refers to a measure of similarity between a previously characterized protein with the listed annotation and an amino acid sequence translated from the contig. A high score indicates substantial amino acid identity between the two proteins.

**Figure 3:**
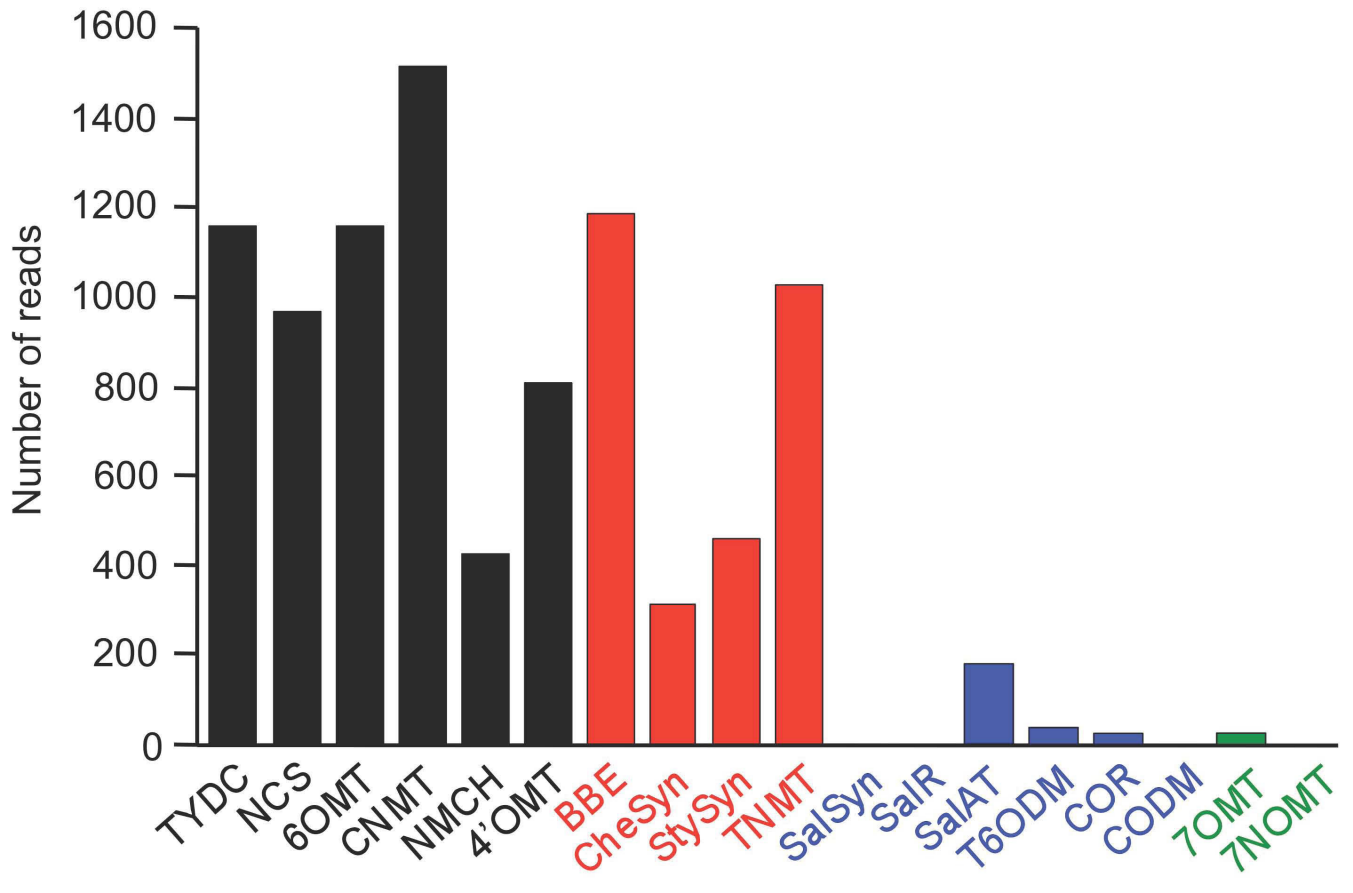
**Number of 454 pyrosequence reads representing gene transcripts corresponding to known benzylisoquinoline alkaloid biosynthetic enzymes **. The cDNA library used for 454 pyrosequencing was prepared from opium poppy cell cultures treated with a fungal elicitor for 10 h. Sequence counts include unigenes encoding predicted proteins with > 90% amino acid sequence identity to known opium poppy enzymes except for CheSyn and StySyn, which were compared with known enzymes from *Eschscholzia californica*. Black bars represent unigenes encoding enzymes involved in the conversion of precursor tyrosine to the central intermediate (*S*)-reticuline. Red bars refer to unigenes encoding enzymes involved in the formation of sanguinarine, blue bars represent unigenes encoding enzymes involved in the biosynthesis of morphine, and green bars correspond to other enzymes with a role in benzylisoquinoline alkaloid metabolism. Abbreviations are as indicated in Figure 1 and Additional File 1.

Local BLASTx analysis was performed to identify ESTs encoding all known BIA biosynthetic enzymes (Figure 3). In the pathway from tyrosine to (S)-reticuline (Figure 1), sequence reads corresponding to known enzymes were relatively abundant with CNMT showing the highest transcript level and the P450-dependent enzyme NMCH displaying the lowest transcript abundance (Figure 3). Several unigenes showed less than 90% amino acid sequence identity compared with 6OMT and 4'OMT suggesting that the corresponding transcripts encode O-methyltransferases that potentially accept BIA substrates other than norcoclaurine and 3'-hydroxy-N-methylcoclaurine, respectively. In the branch pathway from (S)-reticuline to sanguinarine, sequence reads encoding known enzymes were similarly abundant with the P450-dependent enzymes CheSyn and StySyn showing the lowest transcript levels. It should be noted that the transcript levels shown in Figure 3 reflect the total of all putative isoforms (i.e. unigenes with > 90% amino acid identity compared with functionally verified genes); thus, individual NCS, CNMT, BBE, StySyn and TNMT unigenes were assembled from the largest number of sequence reads (Table 2; Figure 2) although the total number of ESTs corresponding to all putative isoforms was higher for certain other enzymes (Figure 3). For example, no individual unigenes encoding TYDC were found among the 50 most abundant transcripts (Table 2) although the total number of reads for all unigenes encoding TYDC was substantial suggesting the occurrence of several different isoforms (Figure 3). Unigenes encoding CheSyn and StySyn were identified on the basis of their similarity to characterized cDNAs from *Eschscholzia californica*[18,19] and their sequences have been deposited in GenBank accession numbers GU325749 and GU325750 respectively.

Transcripts encoding three out of six known enzymes involved in the conversion of (S)-reticuline to morphine were not detected in the database, with ESTs corresponding to the other three enzymes represented at substantially lower levels than enzymes involved in sanguinarine biosynthesis (Figure 3; Additional File 1). Similarly, transcripts encoding two other known BIA biosynthetic enzymes from opium poppy were represented at low levels (e.g., 7OMT, N7OMT) or were not found in the database (Figure 3; Additional File 1). The failure of elicitor-treated opium poppy cell cultures to express genes encoding SalSyn, SalR and CODM explains the absence of morphine in dedifferentiated cells. It is notable that all unigenes encoding enzymes involved in sanguinarine biosynthesis displayed full-length open reading frames, which further supports the depth of transcriptome coverage that is possible using 454 pyrosequencing.

### Deep proteome analysis of elicitor-treated opium poppy cell cultures

**Figure 4:**
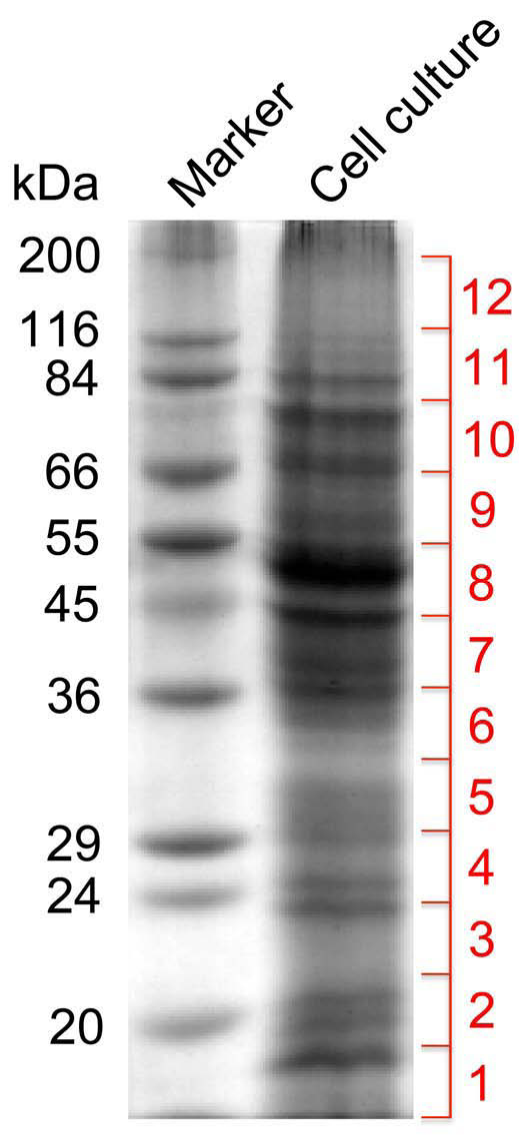
**Fractionation of the gel containing proteins separated by SDS-PAGE prior to LC-MS/MS **. Coomassie stained gel of a total protein extract (10 μg) from opium poppy cell cultures treated with a fungal elicitor for 50 h. Each of the 12 gel slices was treated with trypsin and independently analyzed by LC-MS/MS peptide analysis.

A total protein extract from elicitor-treated opium poppy cells was fractionated by one-dimensional SDS-PAGE and the gel was cut into 12 equal-sized fragments (Figure 4). Proteins in each gel slice were digested with trypsin and subjected to LC-MS/MS. Peptide fragment spectra were used to search both the public NCBI non-redundant green plant protein database and the opium poppy database created by 454 pyrosequencing. Using a stringent cut-off, 288 peptides and polypeptides were identified using the public protein database, of which 177 were represented by two or more peptides. In contrast, 1,004 peptides and polypeptides were identified using the opium poppy-specific 454 pyrosequencing database, of which 571 were represented by two or more peptides (Additional File 5). The species-specificity and depth of coverage offered by the 454 pyrosequencing database added substantial identification power to the analysis.

**Figure 5:**
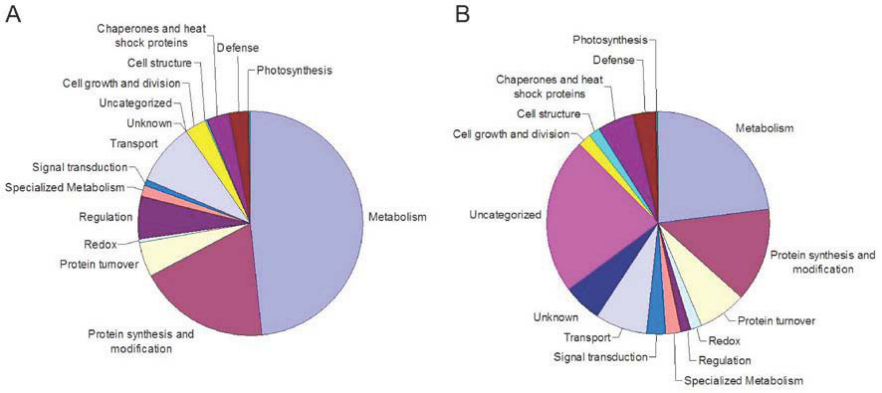
**Functional categories of (A) trans cripts represented in the 454 pyrosequence database and (B) peptides identified by LC-MS/MS **. (A) GO annotations were assigned for 23,753 contigs and singletons out of a total of 93,723 unigenes in the opium poppy 454 pyrosequencing database. (B) GO annotations were assigned for a total of 1,004 putative opium poppy proteins identified by LC-MS/MS peptide analysis.

Annotated unigenes in the 454 pyrosequencing database and proteins identified by LC-MS/MS peptide analysis were classified into functional categories based on their putative roles in cellular processes (Figure 5). Putative GO annotations could be assigned to 72% of the peptides and polypeptides with corresponding ESTs, whereas the remaining 28% belong to unknown, uncategorized and no hit categories (Figure 5B). The most abundant category (e.g., metabolism) represented 23% of all identified proteins and included enzymes involved in primary metabolism, such as SAM synthetase, methionine synthase and enzymes involved in central metabolic pathways such as glycolysis and the tricarboxylic acid cycle (Additional File 5). A number of peptides and polypeptides (14%) were associated with protein synthesis and modification suggesting a substantial role for these processes in elicitor-treated opium poppy cell cultures (Figure 5B). Chaperones and heat shock proteins (5%), defense proteins (3%) and proteins involved in protein turnover (7%) were also well represented. Almost all enzymes involved in BIA biosynthesis that were represented in the 454 pyrosequencing database were identified by LC-MS/MS peptide analysis (Additional File 5), including NCS, 6OMT, CNMT, NMCH, 4'OMT, BBE, StySyn, and TNMT. The morphine biosynthetic enzyme COR1 was also identified. Interestingly, TYDC and CheSyn were not identified despite similar transcript levels compared with other soluble and P450-dependent enzymes, respectively (Figure 3).

### Integration of transcriptome and proteome databases

**Figure 6:**
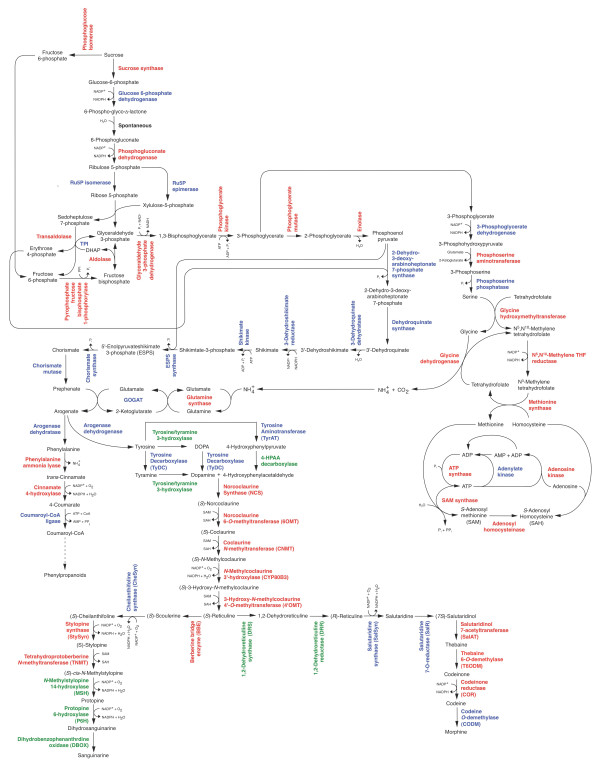
**Metabolic networks from sucrose to sanguinarine and morphine**. Gene transcripts corresponding to enzymes shown in black or red were identified in the 454 pyrosequencing database, whereas those written in grey were not. Enzymes written in red were found among proteins identified by LC-MS/MS peptide analysis. Cognate cDNAs have not been isolated for enzymes shown in blue.

A broad survey of cellular metabolism involved in the conversion of sucrose to sanguinarine resulted in the identification of transcripts or proteins corresponding to a substantial number of metabolic enzymes (Figure 6). With the exception of 3-dehydroquinate dehydratase, all enzymes required for the formation of tyrosine and SAM were represented in the 454 pyrosequencing database, whereas 20 out of 36 enzymes were found in the LC-MS/MS-generated protein database (Additional File 5). Many of these enzymes were also among the top-50 most abundant unigenes (Table 2). Notably, four independent unigenes in the top 50 encoded enzymes involved in the metabolism of SAM, the methyl donor for the various O-and N-methyltransferases in BIA biosynthesis.

The remaining sanguinarine biosynthetic enzymes for which cognate cDNAs have not been isolated catalyze three of the four steps involved in the formation of dopamine and 4-HPAA, and the final three conversions from (S)-cis-N-methylstylopine to sanguinarine (Figure 6). Some of these enzymes likely belong to known protein families including the cytochromes P450 MSH and P6H [21,22] and the oxidoreductase DBOX [23,24]. Candidate proteins with substantial identity to oxidoreductase and other enzyme categories potentially involved in BIA metabolism were found in the LC-MS/MS-generated peptide and polypeptide database (Additional File 6).

## Discussion

Integration of 454 pyrosequencing and LC-MS/MS peptide analysis were used to survey the transcriptome and proteome, respectively, of elicitor-treated opium poppy cell cultures. The depth of each database provides new insights into the regulation of BIA metabolism and plant defense responses, establishes valuable resources for the discovery of new alkaloid biosynthetic genes, and allows an assessment of next-generation-omics technologies as tools to study natural product biosynthesis in plants that currently lack genome sequence resources [37].

Treatment of cell cultures with the elicitor for 10 and 50 h maximized the accumulation of BIA biosynthetic gene transcripts and cognate enzymes, respectively. Using microarray and northern blot analyses, the maximum induction of BIA biosynthetic genes was previously shown to occur 10 h after the elicitor treatment of opium poppy cell cultures [1]. Corresponding western blot analysis showed that BIA biosynthetic enzyme levels were highest 50 h after elicitor treatment [2,38]. The elicitor-induced accumulation profiles of protopine and sanguinarine (Additional File 2) were in agreement with the temporal induction of BIA products and pathway intermediates determined using FT-ICR-MS [1].

The EST database generated by 454 pyrosequencing was compared with that established by random sequencing of clones from an elicitor-treated opium poppy cell culture cDNA library using dideoxy chain-termination (Sanger) technology [1]. Although the average read length produced by 454 pyrosequencing was less than that of Sanger-based sequencing (462 bp and 653 bp, respectively), substantially more ESTs were generated (427,369 and 10,224, respectively), which led to the acquisition of a large number of additional unigenes (93,723 and 7,225, respectively; Table 1). The most abundant transcripts in the 454 pyrosequencing database encoded enzymes and proteins involved in metabolism, defense, signaling, transport and cellular structure (Table 2). Transcripts encoding biosynthetic enzymes involved in the regeneration of (S)-adenosylmethionine (i.e. SAM synthetase, S-adenosylhomocyteinase and methionine synthase) were among the most abundant in the database, which is in agreement with their widespread occurrence in an EST database generated by Sanger sequencing [1].

An EST database was established using 454 pyrosequencing to investigate the flavonoid pathway in the Chinese medicinal plant *Epimedium sagittatum*[39]. A total of 217,380 reads with an average length of 225 bp were assembled into 76,459 unigenes consisting of 17,231 contigs and 59,228 singletons. Similarly, 454 pyrosequencing of cDNA obtained from the glandular trichomes of *Artemisia annua*, which produces the antimalarial sesquiterpene artemisinin, yielded 406,044 reads with and average length of 210 bp asembling into 42,678 contigs and 147,699 singletons [40]. Real time-PCR confirmed the expression of all known terpenoid biosynthetic genes and revealed several novel gene transcripts in the 454 pyrosequencing database including putative sesquiterpene synthase homologues. Our opium poppy cell culture library is comparable or superior to these reported databases in terms of sequence coverage (Table 1). The large number singletons in the opium poppy cell culture database was also reported in the other systems and might result from assembly errors due or from the occurrence of low abundance transcripts. As shown for TNMT (Figure 2; Additional file 4) several unigenes were found for most BIA biosynthetic enzymes (i.e. using the criterion of > 90% amino acid identity), which could reflect the occurrence of multiple gene family members. Separate unigenes encoding proteins with 100% amino acid identity could also result from the improper assembly of contigs (Figure 2; Additional file 4). Clearly, the reported 93,723 unigenes is a substantial overestimate of the actual number of transcripts expressed in those cells. The lack of assembly potentially caused by sequencing errors could account for the high number of orphaned ESTs, although some could represent low-expression level genes.

Our 454 pyrosequencing reads showed a substantial bias for the 3'-end of gene transcripts most likely due to the priming of the first-strand cDNA synthesis using oligo-dT (Figure 2). Moreover, the nebulization of relatively short, partial cDNAs process was also inefficient (Figure 2). Comparison of 454 pyrosequencing using California poppy (*Eschscholzia californica*) cDNA libraries prepared using oligo-dT or random-primers confirmed that the 3'-end bias resulted from priming first-strand cDNA synthesis with oligo-dT [41]. Assembly of both the oligo-dT and random-primed ESTs generated from two full plate of GS-FLX 454 pyrosequencing resulted in 120,585 unigenes with an average length of 157 bp which assembled into 30,603 contigs and 89,892 singletons [41]. Many of the partial ESTs are also truncated at common points at their 5'-end (Figure 2). Genomic 454 pyrosequencing of Escherichia coli showed that 80% of single nucleotide polymorphisms were falsely linked to reads having the same starting point [42]. The high number of replicate sequences suggested that the phenomenon was not random and was associated with the emulsion PCR step and not with nebulization. Similar artefacts were also reported in a metagenomics study on several species, which found systematic errors in genomes sequenced by 454 pyrosequencing technologies (i.e. GS20 or GS-FLX) [43]. Multiple reads from a single template were suggested to occur when amplified DNA attaches to empty beads during emulsion PCR. Although duplicate sequences are occasionally removed prior to assembly, clearly some are still present in our database (Figure 2).

Since the number of 454 pyrosequencing reads in each contig is directly proportional to the abundance of specific cDNAs in the library, quantification of the data provides an accurate measure of the relative expression level of selected transcripts. Comparisons of 454 pyrosequencing and hybridization-based gene expression analyses (i.e. DNA microarray or northern blot) have shown reproducible correlations [32,35,44]. The induction of all known genes encoding BIA biosynthetic enzymes involved in the formation of sanguinarine has been shown previously [1]. However, 454 pyrosequencing provides the ability to quantify the relative abundance of different gene transcripts (Figure 3). In the conversion of tyrosine to sanguinarine (Figure 1),454 pyrosequencing reads encoding CNMT and TNMT were most abundant and were represented among the top-50 most highly expressed unigenes (Table 2). In contrast, reads corresponding to the P450-dependent enzymes NMCH, CheSyn and StySyn were the least abundant (Figure 3). Overall, elicitor-treated opium poppy cell cultures show a strong commitment to sanguinarine biosynthesis as demonstrated by the cumulative abundance of 454 pyrosequencing reads corresponding to genes involved in sanguinarine biosynthesis in agreement with previously published reports comparing control and elicitor-treated opium poppy cell cultures [1]. The total number of reads (i.e. 8,505) with greater than 90% amino acid identity to all known BIA biosynthetic enzymes represent approximately 2.0% of the transcriptome. Of these, 62.5% encoded enzymes leading to (S)-reticuline, 35.0% corresponded to enzymes involved in the conversion of (S)-reticuline to sanguinarine, 2.4% represented enzymes in the morphinan alkaloid branch pathway, and 0.1% were relevant to the formation of other BIAs, such as laudanine (Figure 1 and Additional File 1). The absence of any 454 pyrosequncing reads corresponding to three (i.e. SalSyn, SalR and CODM) out of the six enzymes leading from (S)-salutaridine to morphine explains the lack of codeine or morphine in elicitor-treated opium poppy cell cultures (Figure 3). Interestingly, genes encoding other known biosynthetic enzymes in the morphinan alkaloid branch pathway (i.e. SalAT, T6ODM and COR) were expressed, albeit at low levels compared with those involved in the formation of (S)-reticuline or sanguinarine (Figure 3). DNA microarray and northern blot analyses have previously shown that SalAT mRNA levels were induced in opium poppy cell cultures in response to elicitor treatment, whereas COR transcripts are constitutive [1]. Moreover, the recently identified T6ODM cDNA was represented in a Sanger-based opium poppy cell culture EST database, whereas the CODM was only found in an opium poppy stem EST database [29]. The differential expression of genes encoding morphinan biosynthetic enzymes in elicitor-treated opium poppy cells could reflect a requirement for specific cellular or developmental conditions not present in dedifferentiated cell cultures [38,45].

Two-dimensional (2D) SDS-PAGE is the most commonly used method to separate proteins for plant proteomics applications. However, penetration into the proteome is limited owing mostly to low abundance proteins that are difficult isolate from the 2D gel. Our previous application of LC-MS/MS to analyze the proteome of elicitor-treated opium poppy cell cultures resulted in the identification of 219 proteins based on peptide fragment fingerprint searches using a combination of public and opium poppy EST (i.e. Sanger sequenced) databases [2]. A total of 340 spots were isolated by 2D SDS-PAGE and 6OMT was the only BIA biosynthetic enzyme represented among the identified proteins [2]. Recently, the mass spectral analysis of proteins partially separated by one-dimensional SDS-PAGE was used to assemble an impressive proteome map for Arabidopsis thaliana for different organs, developmental stages, and undifferentiated cultured cells [46]. A total of 86,456 peptide matches yielded 13,029 identified proteins. Proteomics in many plants is typically forced to rely on cross-species identification owing to the lack of genome or transcriptome sequence information, which leads to relatively low numbers of unambiguously identified proteins and the potential for false-positive identification. For example, proteome analysis of banana, which is distantly related to most plant species with substantial DNA sequence data, was used to compare various protein extraction methods and one-dimensional versus 2D SDS-PAGE techniques within the context of cross-species matching of peptide mass spectra. One-dimensional SDS-PAGE on proteins extracted in chloroform:methanol (5:4) followed by LC-MS/MS facilitated the identification of the most proteins, including several hydrophobic proteins that were underrepresented when 2D SDS-PAGE was used [47].

Our one-dimensional SDS-PAGE, LC-MS/MS proteomics approach coupled with the unambiguous identification of peptide spectra using an extensive opium poppy EST database generated by 454 pyrosequencing led to the identification of five-fold more peptides and polypeptides (1,004 in total) than our previous effort [2]. Most identified proteins are involved in metabolism, defense, signalling, transport and cellular structure (Figure 5, Additional file 5). The majority of identified transcripts (Figure 5A) and proteins (Figure 5B) are involved in metabolism and include enzymes of primary metabolic pathways, such as glycolysis and the tricarboxylic acid cycle, and intermediary metabolic enzymes, such as SAM synthetase and methionine synthase (Additional File 5; Figure 6). Almost all enzymes involved in BIA biosynthesis were identified by LC-MS/MS peptide analysis (Additional File 5; Figure 6). Since the likelihood of identifying a certain protein is proportional to the abundance of specific peptides, the absence of TYDC in the identified protein list (Additional File 5) could be due to the large number of isoforms encoded by the approximately 15-member TYDC gene family [7]. Alternatively, the absence of TYDC in the identified protein list (Additional File 5) could result from issues related to solubility, extraction efficiency, digestion, or the nature of peptide sequences.

With one exception (i.e. 3-dehydroquinate dehydratase), transcripts encoding all enzymes required for the formation of tyrosine and SAM were represented in the EST database (Figure 6) and several were also among the 50 most abundant unigenes (Table 2). Similarly, many of these enzymes were also found in the protein database (Additional File 5; Figure 6). Altogether these results further demonstrate the metabolic commitment of elicitor-treated opium poppy cell cultures to the overall biosynthesis of sanguinarine.

Based on the abundance of characterized mRNAs and enzymes involved in BIA metabolism, the remaining components of sanguinarine biosynthesis for which cognate cDNAs have not been isolated should be represented in the transcript and protein databases at similar levels. The penultimate and third-to-last enzymes in sanguinarine biosynthesis, which catalyze the conversion of (S)-cis-N-methylstylopine to dehydrosanguinarine (Figure 1), are cytochromes P450 [21,22]. Although the transcript database contains numerous sequences that annotated as cytochromes P450, a more focused number of candidate cytochromes P450 were represented in the protein database (Additional File 6). Cytochromes P450 represented in the 454 pyrosequencing database at levels similar to transcripts encoding NMCH, CheSyn and StySyn coupled with the corresponding representation of the cognate enzymes in the LC-MS/MS protein database provides a basis for the selection of genes putatively involved in BIA biosynthesis.

## Conclusions

The integration of state-of-the-art 454 GS-FLX Titanium pyrosequencing and LC-MS/MS-based protein profiling technologies is an effective strategy to establish deep transcriptome and proteome databases for the investigation of natural product metabolism in non-model plant systems. A near-complete transcriptome from relatively homogenous, elicitor-treated opium poppy cell cultures could be achieved by the assembly of fewer than one-half million 454 pyrosequencing reads using the GS-FLX Titanium technology. All known sanguinarine biosynthetic gene transcripts were represented in the database, along with a multitude of transcripts encoding primary and intermediary metabolic enzymes providing precursors and co-substrates in support of alkaloid production. The absence of several transcripts encoding morphinan alkaloid biosynthetic enzymes reveals the transcriptional basis for the lack of morphine production in dedifferentiated opium poppy cell cultures. The establishment of tandem mass spectra derived from predicted peptides represented in the deep transcript database facilitated the empirical identification of a large number of corresponding proteins fractionated by one-dimensional SDS-PAGE. The depth of proteome coverage was dramatically greater than that achieved using 2D SDS-PAGE to isolate individual proteins base on the representation of most sanguinarine biosynthetic enzymes and a substantial number of metabolic enzymes relevant to BIA metabolism. The integration of a near-complete and species-specific transcript database was crucial to the successful identification of multiple peptides. Profiling of the more abundant proteins in elicitor-treated opium poppy cell cultures also revealed a number of uncharacterized enzymes that potentially catalyze steps in sanguinarine biosynthesis.

## Methods

### Cell culture and elicitor treatment

Cell suspension cultures of opium poppy (*Papaver somniferum*) cv Marianne, cell line 2009 [48], were grown at 23°C on a gyrotary shaker at 125 rpm in Gamborg 1B5C medium [49] containing B5 salts and vitamins, 20 g/L sucrose, 1 g/L casein hydrolysate and 1 mg/L 2,4-dichlorophenoxyacetic acid. The cell cultures were sub-cultured biweekly using a 1:3 dilution of inoculum to fresh medium. Elicitor treatment was performed by adding 1 ml of *Botrytis cinerea* homogenate to 50 ml of cultured cells in rapid growth phase (2 days after sub-culture) and grown for an additional 10 h or 50 h for the isolation of RNA or protein, respectively. Cells were collected by vacuum filtration and stored at -80°C. The fungal elicitor was prepared by inoculating 50 ml of 1B5C medium lacking 2,4-dichlorophenoxyacetic acid with 1 cm^3 ^of *B. cinerea* mycelium. The fungal culture was grown at 120 rpm on a gyratory shaker at 22°C in the dark for 1 week. Fungal mycelia and medium were homogenized in a blender (Waring Instruments, Torrington, CT), autoclaved at 121°C for 20 min and stored at -20°C.

### R NA extraction, cDNA library construction, 454 pyrosequencing and data processing

Cell cultures treated with the fungal elicitor for 10 h were ground to a fine powder under liquid nitrogen and total RNA was isolated according to [50]. Poly(A)^+ ^RNA was extracted by two rounds of Dynabeads oligo(dT)-based purification according to the manufacturer's instruction (Invitrogen, Carlsbad, CA). A double-stranded cDNA library was prepared using a protocol optimized for 454 pyrosequencing developed by the Joint Genome Institute http://www.jgi.doe.gov/[51]. The cDNA library was randomly sheared and sequenced using Titanium FLX series reagents on a Genome Sequencer FLX instrument (454 Life Sciences, Branford, CT). A total of 427,369 high-quality expressed sequence tags (ESTs) with an average read length of 462 bp were obtained after processing the raw data to eliminate low-quality sequences and poly(A) tails. Pre-processing of the data included the removal of reads with BLASTn expectation values of e < 10^-20 ^with respect to a plant repeat sequence database [52], the trimming of poly(A/T) tails, the removal of low-complexity sequences using the mdust filtering program http://www.tigr.org/tdb/tgi/software, and the elimination of reads shorter than 40 bp. Clustering was done using the TGI Clustering Tools software http://compbio.dfci.harvard.edu/tgi/software. Unigenes were annotated by BLASTx analysis against the Uniprot Plants 9.2 public database.

### Protein extraction and SDS-PAGE

Opium poppy cells (1 g) treated with the fungal elicitor for 50 h were ground to a fine powder under liquid nitrogen and extracted in 0.5 M Tris-HCl, pH 7.5, 50 mM EDTA, 1% (w/v) SDS, and 2% (w/v) 2-mercaptoethanol. The extract was centrifuged at 15,000 g and the supernatant extracted with an equal volume of phenol. Subsequently, the emulsion was centrifuged at 15,000 g to separate the phases. The aqueous phase was discarded and an equal volume of extraction buffer was vigorously mixed with the phenol phase. The emulsion was centrifuged and the phenol phase was recovered. Five volumes of methanol containing 0.1 M ammonium acetate and 0.068% (v/v) 2-mercaptoethanol were added, and the mixture incubated overnight at -20 °C. Precipitated proteins were collected by centrifugation at 15,000 g and washed twice with the methanol solution. The pellet was dried and dissolved in rehydration buffer (7 M urea, 2 M thiourea, 56 mM dithiothreitol, and 2.5% (v/v) 3-[(3-cholamidopropyl)dimethylammonio]-1-propane-sulfonic acid (CHAPS)). Protein concentration was determined using the RC DC protein assay (BioRad, Hercules, CA). Ten micrograms of total proteins were separated by SDS-PAGE. To evaluate the quality of the electrophoretic separation, the gel was stained with Coomassie Brilliant Blue. The lane on the gel containing protein was cut into 12 equal segments (Figure 2). Proteins in each gel segment were digested with trypsin prior to LC-MS/MS.

### Mass spectrometry and spectrum data analysis

Tryptic protein digests were analyzed using an Agilent 1100 LC-Ion-trap-XCT-Ultrasystem (Agilent Technologies, Santa Clara, CA) fitted with an integrated fluidic cartridge for peptide capture, separation and nanospraying (HPLC-Chip technology) as described previously [2]. Injected protein samples were trapped and desalted on a pre-column channel (40-nl volume; Zorbax 300 SC-C_18_) for 5 min with 0.2% (v/v) formic acid delivered by an auxiliary pump at 4 μl/min. The peptides were then reverse-eluted from the trapping column and separated on the analytical channel (43-mm channel length; Zorbax 300 SC-C_18_) at 0.3 μl/min. Peptides were eluted using a 5-70% (v/v) acetonitrile gradient in 0.2% (v/v) formic acid over 10 min. MS/MS spectra were collected by data-dependent acquisition, with parent ion scans of 8100 Th/s over m/z 400-2000 and MS/MS scans at the same rate over m/z 100-2200. Peak-list data were extracted from these files by the DataAnalysis software for the 6300 series ion trap, v3.4 (build 175). Mascot v2.1 (Matrix Science, Boston, MA) was used to search the MS/MS data using the following parameters: 1.6 Da precursor ion mass tolerance, 0.8 Da fragment ion mass tolerance, 1 potential missed cleavage, carbamidomethyl modification of cysteine and variable oxidation of methionine. Peptide sequence data was used to search the Viridiplantae (green plants) database (containing 468,052 sequences) in NCBI http://www.ncbi.nlm.nih.gov. The peptide sequence data was then used to query the elicitor-treated opium poppy cell culture 454 EST database (containing 427,369 sequences) in all potential open reading frames using Mascot v2.1. Results were indexed with the aid of a prior-clustering and annotation exercise. In all cases, human and Botrytis cinerea proteins were included in the searches to avoid contaminant-based erroneous assignment of the data.

Protein hits were scored based on the quality and abundance of the underlying peptide MS/MS data and their scores. A cut-off score (p < 0.012) of 56 with a false discovery rate of 1% was used for all peptides identified through matches in the public databases, and a cut-off score (p < 0.05) of 46 with a false discovery rate of 1.13% was used for all peptides identified through matches in the 454 EST database. The resulting MS/MS spectra were manually assessed for consistency with the proposed sequences and distance from the next highest scoring peptide(s). The protein names associated with each hit were determined by selecting the highest scoring entry and the most common name representing the dataset.

### Metabolite extraction and HPLC analysis

Frozen cell cultures (1 g) were ground to a fine powder under liquid nitrogen and extracted for 2 h in 100% (v/v) methanol at room temperature. The extracts were centrifuged for 10 min to pellet debris and the supernatants were reduced to dryness under reduced pressure. Pellets were resuspended in 100 μl 100% (v/v) methanol. Ten microliters of each extract was diluted in 100 μl of 98% (v/v) H_2_O: 2% (v/v) acetonitrile: 0.04% (v/v) H_3_PO_4 _and analyzed using a System Gold HPLC and photodiode array detector (Beckman-Coulter, Mississauga, Canada). All separations were performed at a flow rate of 1.5 ml/min on a LiChrospher RP-Select B 5μ column 150 × 4.6 mm (Alltech, Illinois, USA). Separation was achieved using a gradient of solvent A [98% (v/v) H_2_O: 2% (v/v) acetonitrile: 0.04% (v/v) H_3_PO_4_] and solvent B [98% (v/v) acetonitrile: 2% (v/v) H_2_O: 0.04% (v/v) H_3_PO_4_]. Chromatography was initiated and maintained for 5 min using 90% solvent A. Subsequently, the gradient was ramped to 35% solvent B over 40 min and then to 100% solvent B over 5 min. Peaks corresponding to reticuline, protopine and sanguinarine were monitored at 210 nm and identified on the basis of their retention times and UV spectra compared with authentic standards. Dextromethorphan was used as an internal standard for the quantification of data.

### Accession numbers

The sequences described in this paper have been submitted to GenBank under the accession numbers GU325749 and GU325750.

## Authors' contributions

IDP carried out all experimental work, with the exception of the bioinformatics and proteomics. DC and JN performed the bioinformatics on the 454 pyrosequencing data. MFK and DCS conducted the LC-MS/MS peptide analysis. IDP and PJF designed the experiments. IDP wrote the manuscript and PJF was its primary editor. All authors read and approved the final manuscript.

## Supplementary Material

Additional file 1 **Biosynthetic pathways leading to morphine (A), laudanine (B) and norlaudanine (C)**. Enzymes for which cognate cDNAs have been isolated are shown in black. Abbreviations: DRS, 1,2-dehydroreticuline synthase; DRS, 1,2-dehydroreticuline reductase; SalSyn, salutaridine synthase; SalR, salutaridine reductase; SalAT, salutaridinol 7-O-acetyltransferase; THS, thebaine synthase; T6ODM, thebaine 6-O-demethylase; COR1, codeinone reductase 1; CODM, codeine O-demethylase; 7OMT, (R,S)-reticuline 7-O-methyltransferase; N7OMT, (R,S)-norreticuline 7-O-methyltransferase. Click here for file

Additional file 2 **Alkaloid content of opium poppy cells after elicitor treatment**. Reticuline (blue), protopine (yellow) and sanguinarine (red) levels in opium poppy cell cultures at various times after elicitor treatment. Click here for file

Additional file 3 **Summary of characteristics for the 454 pyrosequencing database**. (A) Number of new unigenes discovered per 10,000 sequences. (B) Frequency distribution of unigene length after sequence assembly. (C) Frequency distribution of the percentage of full-length open reading frame coverage among unigenes with > 50% or higher amino acid identity. Click here for file

Additional file 4 **Unigenes that annotate as TNMT in the 454 pyrosequencing database**. Click here for file

Additional file 5 **List of peptides and polypeptides identified by LC-MS/MS analysis**. Color coding: known enzymes involved in sanguinarine biosynthesis (yellow); enzymes involved in the primary metabolism relevant to sanguinarine biosynthesis (green); candidate enzymes potentially involved in benzylisoquinoline alkaloid metabolism. Click here for file

Additional file 6 **Candidate proteins identified by LC-MS/MS and potentially involved in benzylisoquinoline alkaloid metabolism in opium poppy cell cultures**. Click here for file

## References

[B1] Zulak KG Cornish A Daskalchuk TE Deyholos MK Goodenowe DB Gordon PP Klassen D Pelcher LE Sensen CW Facchini PJ Gene transcript and metabolite profiling of elicitor-induced opium poppy cell cultures reveals the coordinate regulation of primary and secondary metabolism Planta 2007 225 1085 1106 10.1007/s00425-006-0419-5 17077972

[B2] Zulak KG Khan MF Alcantara J Schriemer DC Facchini PJ Plant defense responses in opium poppy cell cultures revealed by liquid chromatography tandem mass spectrometry proteomics Molecular & Cellular Proteomics 2009 8 86 98 10.1074/mcp.M800211-MCP200 18682378

[B3] Zulak KG Weljie AM Vogel HJ Facchini PJ Quantitative ^1^H NMR metabolomics reveals extensive metabolomic reprogramming of primary and secondary metabolism in elicitor-treated opium poppy cell cultures BMC Plant Biology 2008 8 5 21 10.1186/1471-2229-8-5 18211706 PMC2257952

[B4] Samanani N Facchini PJ Purification and characterization of norcoclaurine synthase. The first committed enzyme in benzylisoquinoline alkaloid biosynthesis in plants J Biol Chem 2002 277 33878 33883 10.1074/jbc.M203051200 12107162

[B5] Samanani N Liscombe DK Facchini PJ Molecular cloning and characterization of norcoclaurine synthase, an enzyme catalyzing the first committed step in benzylisoquinoline alkaloid biosynthesis Plant J 2004 40 302 313 10.1111/j.1365-313X.2004.02210.x 15447655

[B6] Liscombe DK MacLeod BP Loukanina N Nandi OI Facchini PJ Evidence for the monophyletic evolution of benzylisoquinoline alkaloid biosynthesis in angiosperms Phytochemistry 2005 66 2501 2520 16342378 10.1016/j.phytochem.2005.04.044

[B7] Facchini PJ DeLuca V Differential and tissue-specific expression of a gene family for tyrosine/dopa decarboxylase in opium poppy J Biol Chem 1994 269 26684 26690 7929401

[B8] Facchini PJ Park S-U Developmental and inducible accumulation of gene transcripts involved in alkaloid biosynthesis in opium poppy Phytochemistry 2003 64 177 186 10.1016/S0031-9422(03)00292-9 12946416

[B9] 9. Ounaroon A Decker G Schmidt J Lottspeich F Kutchan TM (R,S)-Reticuline 7-O-methyltransferase and (R,S)-norcoclaurine 6-O-methyltransferase of *Papaver somniferum*-cDNA cloning and characterization of methyl transfer enzymes of alkaloid biosynthesis in opium poppy Plant J 2003 36 808 819 10.1046/j.1365-313X.2003.01928.x 14675446

[B10] Morishige T Choi K-B Sato F In vivo bioconversion of tetrahydroisoquinoline by recombinant coclaurine N-methyltransferase Biosci Biotechnol Biochem 2004 68 939 941 10.1271/bbb.68.939 15118328

[B11] Ziegler J Díaz-Chávez ML Kramell R Ammer C Kutchan TM Comparative macroarray analysis of morphine containing *Papaver somniferum* and eight morphine free *Papaver* species identifies an O-methyltransferase involved in benzylisoquinoline biosynthesis Planta 2005 222 458 471 10.1007/s00425-005-1550-4 16034588

[B12] Pauli HH Kutchan TM Molecular cloning and functional heterologous expression of two alleles encoding (*S*)-N-methylcoclaurine 3'-hydroxylase (CYP80b1), a new methyl jasmonate-inducible cytochrome P-450-dependent monooxygenase of benzylisoquinoline alkaloid biosynthesis Plant J 1998 13 793 801 10.1046/j.1365-313X.1998.00085.x 9681018

[B13] Huang FC Kutchan TM Distribution of morphinan and benzo[c]phenanthridine alkaloid gene transcript accumulation in *Papaver somniferum* Phytochemistry 2000 53 555 564 10.1016/S0031-9422(99)00600-7 10724180

[B14] Morishige T Tsujita T Yamada Y Sato F Molecular characterization of the S-adenosyl-L-methionine: 3'-hydroxy-N-methylcoclaurine 4'-O-methyltransferase involved in isoquinoline alkaloid biosynthesis in *Coptis japonica* J Biol Chem 2000 275 23398 23405 10.1074/jbc.M002439200 10811648

[B15] Dittrich H Kutchan TM Molecular cloning, expression, and induction of berberine bridge enzyme, an enzyme essential to the formation of benzophenanthridine alkaloids in the response of plants to pathogenic attack Proc Natl Acad Sci USA 1991 88 9969 9973 10.1073/pnas.88.22.9969 1946465 PMC52848

[B16] Facchini PJ Penzes C Johnson AG Bull D Molecular characterization of berberine bridge enzyme genes from opium poppy Plant Physiol 1996 112 1669 1677 10.1104/pp.112.4.1669 8972604 PMC158100

[B17] Samanani N Park SU Facchini PJ Cell type-specific localization of transcripts encoding nine consecutive enzymes involved in protoberberine alkaloid biosynthesis Plant Cell 2005 17 915 926 10.1105/tpc.104.028654 15722473 PMC1069708

[B18] Ikezawa N Iwasa K Sato F Molecular cloning and characterization of methylenedioxy bridge-forming enzymes involved in stylopine biosynthesis in *Eschscholzia californica* FEBS J 2007 274 1019 1035 10.1111/j.1742-4658.2007.05652.x 17250743

[B19] Ikezawa N Iwasa K Sato F CYP719A subfamily of cytochrome P450 oxygenases and isoquinoline alkaloid biosynthesis in E schscholzia californica Plant Cell Rep 2009 28 123 133 10.1007/s00299-008-0624-8 18854999

[B20] Liscombe DK Facchini PJ Molecular cloning and characterization of tetrahydroprotoberberine *cis*-N-methyltransferase, an enzyme involved in alkaloid biosynthesis in opium poppy J Biol Chem 2007 282 14741 14751 10.1074/jbc.M611908200 17389594

[B21] Rueffer M Zenk MH Enzymatic formation of protopines by a microsomal cytochrome P-450 system of *Corydalis vaginans* Tetrahedron Lett 1987 28 5307 5310 10.1016/S0040-4039(00)96715-7

[B22] Tanahashi T Zenk MH Elicitor induction and characterization of microsomal protopine-6-hydroxylase, the central enzyme in benzophenanthridine alkaloid biosynthesis Phytochemistry 1990 29 1113 1122 10.1016/0031-9422(90)85414-B

[B23] Ignatov A Clark WG Cline SD Psenak M Krueger J Coscia CJ Elicitation of dihydrobenzophenanthridine oxidase in *Sanguinaria canadensis* cell cultures Phytochemistry 1996 43 1141 1144 10.1016/S0031-9422(96)00540-7 8987906

[B24] Schumacher H-M Zenk MH Partial purification and characterization of dihydrobenzophenanthridine oxidase from *Eschscholtzia californica *cell suspension cultures Plant Cell Rep 1988 7 43 46 10.1007/BF00272975 24241413

[B25] Gesell A Rolf M Ziegler J Díaz Chávez ML Huang F-C Kutchan TM CYP719B1 is salutaridine synthase, the C-C phenol-coupling enzyme of morphine biosynthesis in opium poppy J Biol Chem 2009 284 24432 24442 10.1074/jbc.M109.033373 19567876 PMC2782036

[B26] Ziegler J Voigtlander S Schmidt J Kramell R Miersch O Ammer C Gesell A Kutchan TM Comparative transcript and alkaloid profiling in *Papaver* species identifies a short chain dehydrogenase/reductase involved in morphine biosynthesis Plant J 2006 48 177 192 10.1111/j.1365-313X.2006.02860.x 16968522

[B27] Grothe T Lenz R Kutchan TM Molecular characterization of the salutaridinol 7-*O*-acetyltransferase involved in morphine biosynthesis in opium poppy *Papaver somniferum* J Biol Chem 2001 276 30717 30723 10.1074/jbc.M102688200 11404355

[B28] Unterlinner B Lenz R Kutchan TM Molecular cloning and functional expression of codeinone reductase: the penultimate enzyme in morphine biosynthesis in the opium poppy *Papaver somniferum* Plant J 1999 18 465 475 10.1046/j.1365-313X.1999.00470.x 10417697

[B29] Hagel JM Facchini PJ (2010) Novel O-demethylases of morphine biosynthesis in opium poppy Nature Chem Biol 2010 6 273 275 10.1038/nchembio.317 20228795

[B30] Pienkny S Brandt W Schmidt J Ziegler J Functional characterization of a novel benzylisoquinoline-O-methyltransferase suggests its involvement in papaverine biosynthesis in opium poppy (*Papaver somniferum* L) Plant J 2009 60 56 67 10.1111/j.1365-313X.2009.03937.x 19500305

[B31] Weber AP Weber KL Carr K Wilkerson C Ohlrogge JB Sampling the Arabidopsis transcriptome with massively parallel pyrosequencing Plant Physiol 2007 144 32 42 10.1104/pp.107.096677 17351049 PMC1913805

[B32] Droege M Hill B The Genome Sequencer FLX System-longer reads, more applications, straight forward bioinformatics and more complete data sets J Biotechnol 2008 136 3 10 10.1016/j.jbiotec.2008.03.021 18616967

[B33] Andreas PM Weber KL Weber KC Wilkerson C Ohlrogge JB Sampling the Arabidopsis transcriptome with massively parallel pyrosequencing Plant Physiol 2007 144 32 42 10.1104/pp.107.096677 17351049 PMC1913805

[B34] Torres TT Metta M Ottenwalder B Schlotterer C Gene expression profiling by massively parallel sequencing Genome Res 2008 18 172 177 10.1101/gr.6984908 18032722 PMC2134766

[B35] Gygi SP Rochon Y Franza BR Aebersold R Correlation between protein and mRNA abundance in yeast Mol Cell Biol 1999 19 1720 1730 10022859 10.1128/mcb.19.3.1720 PMC83965

[B36] Bennett MD Smith JB Nuclear DNA amounts in angiosperms Phil Trans Royal Soc London B 1976 274 227 274 10.1098/rstb.1976.0044 6977

[B37] Emrich SJ Barbazuk WB Li L Schnable PS Gene discovery and annotation using LCM-454 transcriptome sequencing Genome Res 2007 17 69 73 10.1101/gr.5145806 17095711 PMC1716268

[B38] Alcantara J Bird DA Franceschi VR Facchini PJ Sanguinarine biosynthesis is associated with the endoplasmic reticulum in cultured opium poppy cells after elicitor treatment Plant Physiol 2005 138 173 183 10.1104/pp.105.059287 15849302 PMC1104173

[B39] Zeng S Xiao G Guo J Fei Z Xu Y Roe BA Wang Y Development of a EST dataset and characterization of EST-SSR s in a traditional Chinese medicinal plant *Epimedium sagittatum* (Sieb. et Zucc.) Maxim BMC Genomics 2010 11 94 42 10.1186/1471-2164-11-94 20141623 PMC2829513

[B40] Wang W Wang Y Zhang Q Qi Yan Guo D Global characterization of *Artemisia annua* glandular trichome using 454 pyrosequencing BMC Genomics 2009 10 465 475 10.1186/1471-2164-10-465 19818120 PMC2763888

[B41] Wall PK Leebens-Mack J Chanderbali AS Barakat A Wolcott E Liang H Landherr L Tomsho LP Hu Y Carlson JE Ma H Schuster SC Soltis DE Soltis PS Altman N dePamphilis CW Comparison of next generation sequencing technologies for transcriptome characterization BMC Genomics 2009 10 347 365 10.1186/1471-2164-10-347 19646272 PMC2907694

[B42] Klopp C Pyrosequencing read bioas: evidences and correction proposal for genome sequencing 2009 http:////www.eadgene.info/Events/NextGenSeqWorkshop2009/tabid/375/Default.aspx

[B43] Gomez-Alvarez V Teal TK Schmidt TM Systematic artifacts in metagenomes from complex microbial communities ISME J 2009 3 1314 1317 10.1038/ismej.2009.72 19587772

[B44] Hornshøj H Bendixen E Conley LN Andersen PK Hedegaard J Panitz F Bendixen C Transcriptomic and proteomic profiling of two porcine tissues using high-throughput technologies BMC Genomics 2009 10 30 10.1186/1471-2164-10-30 19152685 PMC2633351

[B45] Bird DA Franceschi VR Facchini PJ A tale of three cell types: alkaloid biosynthesis is localized to sieve elements in opium poppy Plant Cell 2003 15 2626 2635 10.1105/tpc.015396 14508000 PMC280566

[B46] Baerenfaller K Grossmann J Grobei MA Hull R Hirsch-Hoffman M Yalovsky S Zimmermann P Grossniklaus U Gruissem W Baginsky S Genome-scale proteomics reveals *Arabidopsis thaliana* gene models and proteome dynamics Science 2008 320 938 941 10.1126/science.1157956 18436743

[B47] Vertommen A Panis B Swennen R Carpentier SC Evaluation of chloroform methanol extraction to facilitate the study of membrane proteins of non-model plants Planta 2010 231 1113 1125 10.1007/s00425-010-1121-1 20177697 PMC2840667

[B48] Eilert U Kurz WGW Constabel F Stimulation of sanguinarine accumulation in *Papaver somniferum* cell cultures by fungal elicitors J Plant Physiol 1985 119 65 76

[B49] Gamborg OL Miller RA Ojima K Nutrient requirements of suspension cultures of soybean root cells Exp Cell Res 1968 50 151 158 10.1016/0014-4827(68)90403-5 5650857

[B50] Meisel L Fonseca B Gonzalez S Baeza-Yates R Cambiazo V Campos R Gonzalez M Orellana A Retamales J Silva H A rapid and efficient method for purifying high quality total RNA from peaches (*Prunus persica*) for functional genomics analyses Biol Res 2005 38 83 88 10.4067/S0716-97602005000100010 15977413

[B51] Zhao ZJ Ng D cDNA library creation protocol 2007 http:////my.jgi.doe.gov/general/protocols/SOP_DRAFT_cDNA_library_creation_454

[B52] Ouyang S Buell CR (2004) The TIGR Plant Repeat Databases: A collective resource for identification of repetitive sequences in plants NAR 2004 32 Database D360 363 10.1093/nar/gkh099 14681434 PMC308833

