# Supplementary figures and images for: Integration of deep transcriptome and proteome analyses reveals the components of alkaloid metabolism in opium poppy cell cultures
Source: BMC Plant Biol. 2010 Nov 18;10:252. doi: 10.1186/1471-2229-10-252 (PMC3095332; doi:10.1186/1471-2229-10-252)

# Additional File 1

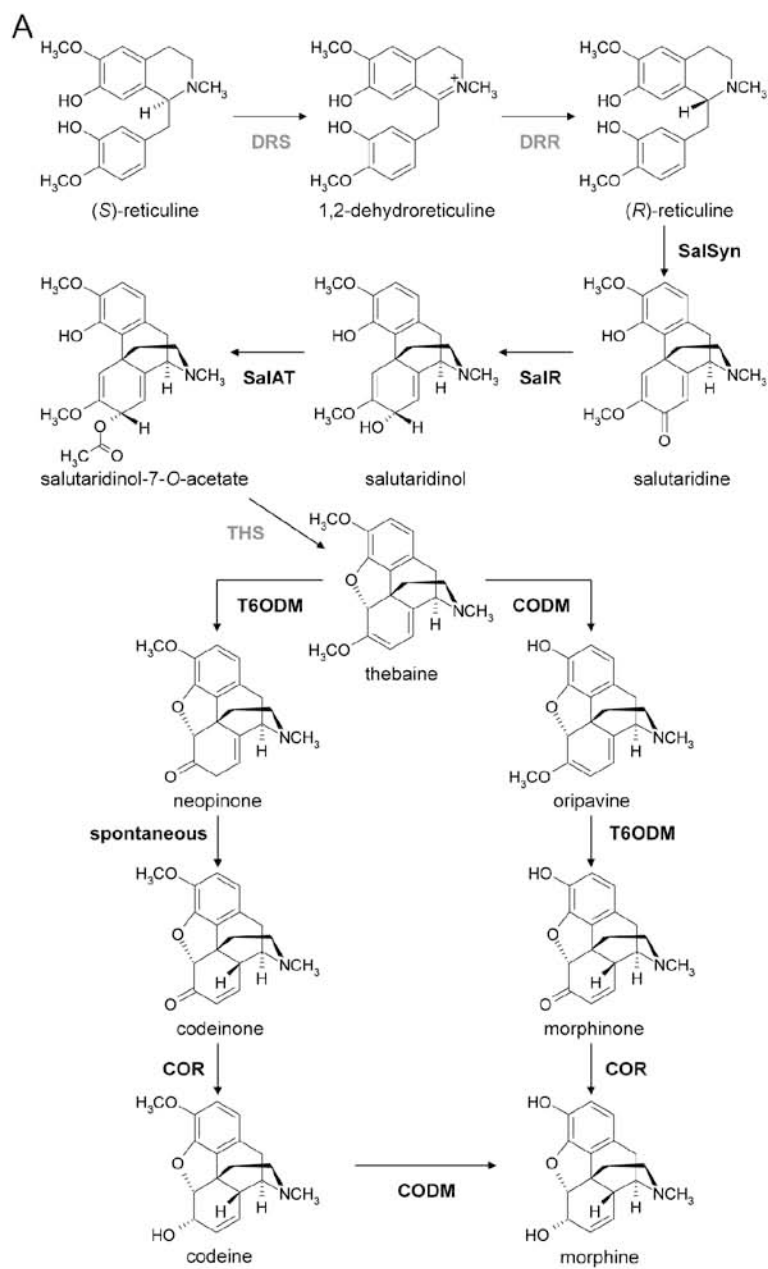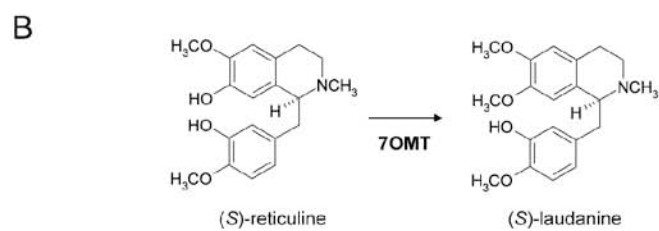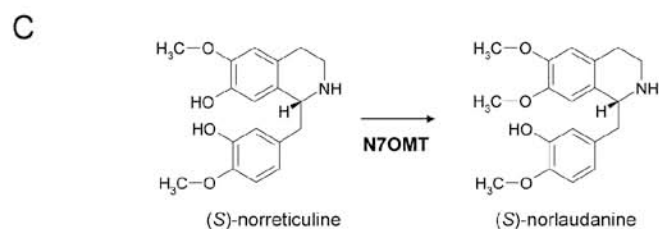

Supplement: Additional file 1 — Biosynthetic pathways leading to morphine (A), laudanine (B) and norlaudanine (C). Enzymes for which cognate cDNAs have been isolated are shown in black. Abbreviations: DRS, 1,2-dehydroreticuline synthase; DRS, 1,2-dehydroreticuline reductase; SalSyn, salutaridine synthase; SalR, salutaridine reductase; SalAT, salutaridinol 7-O-acetyltransferase; THS, thebaine synthase; T6ODM, thebaine 6-O-demethylase; COR1, codeinone reductase 1; CODM, codeine O-demethylase; 7OMT, (R,S)-reticuline 7-O-methyltransferase; N7OMT, (R,S)-norreticuline 7-O-methyltransferase. [file 1471-2229-10-252-S1.PDF]

## Additional File 2

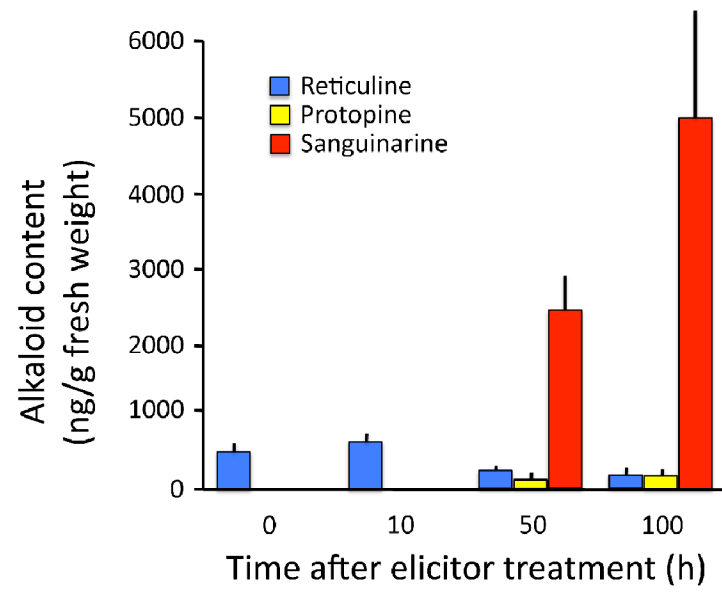

Supplement: Additional file 2 — Alkaloid content of opium poppy cells after elicitor treatment. Reticuline (blue), protopine (yellow) and sanguinarine (red) levels in opium poppy cell cultures at various times after elicitor treatment. [file 1471-2229-10-252-S2.PDF]

### Additional File 3

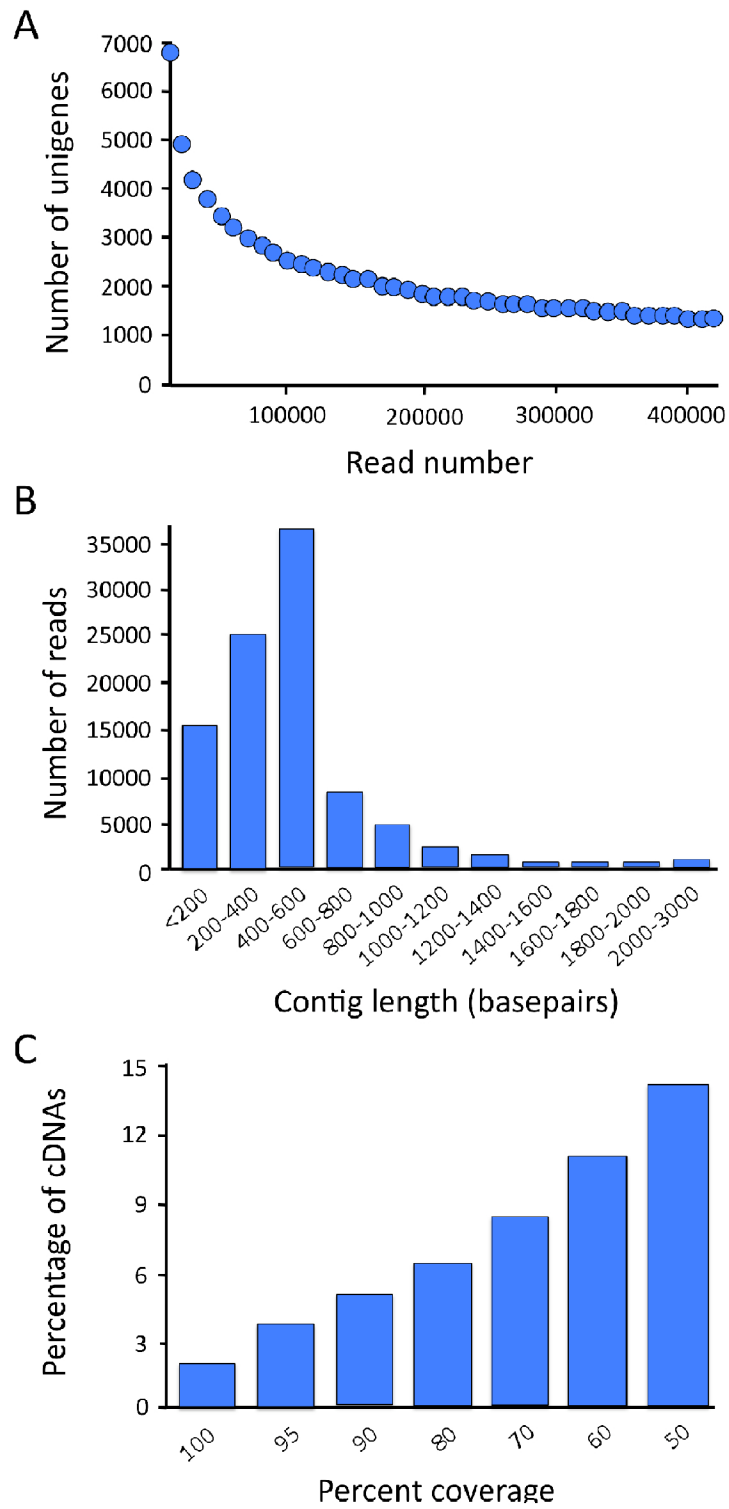

Supplement: Additional file 3 — Summary of characteristics for the 454 pyrosequencing database. (A) Number of new unigenes discovered per 10,000 sequences. (B) Frequency distribution of unigene length after sequence assembly. (C) Frequency distribution of the percentage of full-length open reading frame coverage among unigenes with > 50% or higher amino acid identity. [file 1471-2229-10-252-S3.PDF]
