# Supplementary material for: Integration of deep transcriptome and proteome analyses reveals the components of alkaloid metabolism in opium poppy cell cultures
Source: BMC Plant Biol. 2010 Nov 18;10:252. doi: 10.1186/1471-2229-10-252 (PMC3095332; doi:10.1186/1471-2229-10-252)
Supplement: Additional file 4 — Unigenes that annotate as TNMT in the 454 pyrosequencing database. [file 1471-2229-10-252-S4.PDF]

Additional File 4: List of assembled unigenes with annotations to opium poppy TNMT (accession number Q108P1_PAPSO) in the 454 elicitor-treated opium poppy cell culture library.

Unigene name	Number of ESTs	Amino acid conserved (%)*	Amino acid coverage	TNMT protein score**
Contig1	534	95.5	1-358	1780
Contig2	422	100	93-358	1413
Contig3	23	100	167-293	672
Contig4	16	98.8	278-358	432
Contig5	9	99.3	129-273	750
Contig6	5	94.3	306-358	266
Contig7	4	96.9	294-358	328
Contig8	3	79.4	1-102	371
Contig9	2	99	256-358	543
Contig10	2	86.3	131-198	288
Contig11	2	97.8	296-340	238
Singleton1	1	55.1	243-320	164
Singleton2	1	66.7	104-151	146
Singleton3	1	98.1	307-358	271
Singleton4	1	95	60-159	462
Singleton5	1	90.7	305-358	250
Singleton6	1	100	169-242	383
Singleton7	1	76.9	71-148	295
Singleton8	1	83.7	48-90	166

* Refers to the percentage of the TNMT protein represented by a conserved amino acid sequence translated from the contig.

** Refers to a measure of the similarity between the TNMT protein and an amino acid sequence translated from the contig. A high score means reflects substantial amino acid identity between the TNMT protein and a translation product of the contig.
