# Supplementary material for: Integration of deep transcriptome and proteome analyses reveals the components of alkaloid metabolism in opium poppy cell cultures
Source: BMC Plant Biol. 2010 Nov 18;10:252. doi: 10.1186/1471-2229-10-252 (PMC3095332; doi:10.1186/1471-2229-10-252)
Supplement: Additional file 5 — List of peptides and polypeptides identified by LC-MS/MS analysis. Color coding: known enzymes involved in sanguinarine biosynthesis (yellow); enzymes involved in the primary metabolism relevant to sanguinarine biosynthesis (green); candidate enzymes potentially involved in benzylisoquinoline alkaloid metabolism. [file 1471-2229-10-252-S5.PDF]

Additional File 5: Proteins from elicitor-treated opium poppy cell cultures identified by LC-MS/MS.

Rank	Mass (Da)	Score	Number of peptides	454 sequence hit	Total ESTs	Annotation	Accession number
1	96186	564	18	CL104Contig2	75	Heat shock protein	Q8VZ83_ARATH
2	87062	503	19	CL1Contig4396	19	Heat shock cognate protein 70	Q8GSN3_CUCMA
3	17187	478	15	FQEH0BT01EJ24M	1	Putative heat shock protein 82	Q5QLP0_ORYSJ
4	88365	437	14	CL1Contig6019	88	Chaperonin CPN60-2	CH62_CUCMA
5	30614	408	16	CL1Contig9416	3	Heat shock cognate 70 kDa protein	HSP7C_PETHY
6	107255	398	18	CL146Contig4	46	Phosphoenolpyruvate carboxylase	A9QED9_GOSHI
7	56922	379	14	CL1Contig5215	43	Luminal-binding protein	Q0ZUG6_ISATI
8	39481	367	14	CL1Contig3472	32	Uncharacterized protein	Q307X4_SOLTU
9	16840	360	15	FQEH0BT01BCX9E	1	Heat shock protein 90	A9QVI8_9ASTR
10	56923	330	13	CL32Contig8	80	Beta-tubulin	A7KQH1_EUCGR
11	76980	326	9	CL1Contig6811	46	Ubiquitin activating enzyme E1	O75VJ8_TOBAC
12	39106	326	8	CL95Contig10	32	Uncharacterized protein	A9P8T1_POPTR
13	49199	316	10	CL1Contig4953	39	Elongation Factor EF-2	Q6H4L2_ORYSJ
14	87537	310	9	CL133Contig2	108	Pyruvate kinase	Q94KE3_ARATH
15	30643	302	10	CL32Contig6	7	Beta-tubulin	B5U1R1_9ROSA
16	65624	302	15	CL1Contig5364	81	Heat shock protein 70	Q6L509_ORYSJ
17	47913	302	8	CL1Contig8870	19	Phosphoglycerate kinase	PGKH_TOBAC
18	17582	301	11	FQEH0BT01B6A62	1	Heat Shock protein 82	HSP81_ORYSJ
19	72775	300	9	CL124Contig7	28	Clathrin heavy chain	Q2RBN7_ORYSJ
20	40554	296	9	CL1Contig7394	49	40S ribosomal protein S4	B3TM21_ELAGV
21	25014	291	13	CL1Contig6847	2	Heat shock protein 70	Q6L509_ORYSJ
22	16598	287	8	FQEH0BT01CGK11	1	Uncharacterized protein	A9PBU6_POPTR
23	75603	287	11	CL471Contig2	29	Aminopeptidase-like protein	Q8VZH2_ARATH
24	17185	283	7	CL1Contig7139	3	Fructose bisphosphate aldolase	Q38JJ4_SOLTU
25	22267	276	12	CL1Contig8666	2	Heat shock protein 70	Q40151_SOLLC
26	44843	275	8	CL1Contig332	334	*S*-adenosylmethionine synthetase 2	B5LAW6_CAPAN
27	68350	270	6	CL25Contig3	272	Monodehydroascorbate reductase	B5TV62_CAMSI
28	14341	265	8	FQEH0BT01AESBI	1	Heat shock cognate 70kDa protein	HSP7C_PETHY
29	172455	264	11	CL38Contig1	130	Glutamate synthase	Q93WZ7_PHAVU
30	82828	263	8	CL1Contig8540	227	Elongation Factor EF-2	O9ASR1_ARATH
31	107319	261	10	CL142Contig2	72	Heat shock 70kDa protein	HSP7M_PHAVU
32	26346	260	10	CL1Contig1990	3	Heat shock protein	Q71EE1_HEVBR
33	13388	252	7	FQEH0BT01DSZ1Y	1	ATP synthase beta subunit	A9NUR7_PICSI
34	13805	252	7	FQEH0BT01BPNM2	1	Uncharacterized protein	A2WUX6_ORYSI
35	111873	246	11	CL1Contig9722	62	Actin bundling protein ABP135	Q9SQH4_LILLO
36	17666	244	10	CL1Contig2335	13	Histone H4	Q7XUC9_ORYSJ
37	14838	241	8	FQEH0BT01DGKHF	1	Adenylate translocator	Q0WVD8_ARATH
38	68528	238	7	CL1Contig10324	102	Initiation factor eIF4A-15	Q6T8C6_HELAN
39	67368	235	8	CL124Contig4	18	Clathrin heavy chain	Q39834_SOYBN
40	16641	233	7	FQEH0BT01DADBX	1	Monodehydroascorbate reductase	B5TV62_CAMSI
41	28471	233	7	CL1Contig1732	17	Histone H2B	Q2XPW1_SOLTU
42	32232	233	5	CL1Contig5976	8	Phosphosglycerate kinase	A9P828_POPTR
43	103395	230	9	CL1Contig4019	88	Heat shock protein 83	HSP83_IPONI
44	26483	229	9	CL1Contig237	14	Elongation factor 1-delta	EF1D_PIMBR
45	84313	228	8	CL1Contig6559	52	Heat shock protein	Q0WRS4_ARATH
46	26061	228	6	CL1Contig10545	5	Fructose bisphosphate aldolase	Q2WFK8_9ASTR
47	23423	224	6	CL1Contig3499	16	Luminal-binding protein 5	BIP5_TOBAC
48	16336	222	7	FQEH0BT01DAJBT	1	Ascorbate persoxidase	B6TM55_MAIZE
49	42088	222	7	CL1Contig4847	31	Elongation Factor 2	Q6H4L2_ORYSJ
50	20622	220	8	CL1Contig1807	4	Heat shock protein 82	HSP82_MAIZE
51	52186	219	6	CL1Contig5811	49	*S*-Adenosylmethionine synthetase 2	METK1_VITVI
52	57338	214	9	CL32Contig1	25	Beta-tubulin	TBB1_ARATH
53	43559	214	6	CL1Contig4488	8	ADP ribosylation factor 002	Q38JU3_DAUCA
54	107102	213	11	CL1Contig2799	213	Sucrose synthase	SUSY_SOYBN

55	15534	210	5	FQEH0BT01D55DP	1	Malate dehydrogenase	B2ZAF9_MAIZE
56	29079	210	6	CL1Contig3699	2	*S*-Adenosylmethionine synthetase 5	METK5_VITVI
57	66880	209	7	CL1Contig10068	208	ADP/ATP translocator-like	Q307Y6_SOLTU
58	83519	208	11	CL1Contig7678	125	Uncharacterized protein	A9NUH2_PICSI
59	16886	206	6	CL1Contig4699	3	Methionine synthase	A6YGE7_CARPA
60	24434	203	4	CL1Contig8997	9	*S*-Adenosymethionine synthetase 3	METK3_VITVI
61	66426	201	7	CL232Contig1	83	Elongation factor tu	Q0WUV8_ARATH
62	18094	200	4	FQEH0BT01AK4GT	1	Heat shock protein 70-3	Q67BD0_TOBAC
63	56206	199	6	CL1Contig4601	40	Alpha-tubulin 1	Q84TK6_POPTM
64	12100	199	9	CL1Contig3614	2	Heat shock cognate protein 70	Q8GSN4_CUCMA
65	23789	197	6	CL1Contig9855	2	Uncharacterized protein	A9P9X7_POPTR
66	13590	189	3	FQEH0BT01BTSPM	1	Isocitrate dehydrogenase	Q2WFI2_9ASTR
67	13437	189	2	CL1Contig8463	2	26S proteasome subunit RPN7	A0MKC7_CAPAN
68	70471	188	4	CL396Contig1	62	Uncharacterized protein	Q1KUM7_9ROSI
69	29468	188	7	CL1Contig4249	3	Sucrose synthase	Q9SLY1_CITUN
70	81000	186	6	CL305Contig3	48	Phosphoglucomutase	PGMC_SOLTU
71	51823	185	9	CL4051Contig1	9	Clathrin heavy chain	Q0WNJ6_ARATH
72	25953	182	6	CL1Contig6224	4	Uncharacterized protein	A9PBU6_POPTR
73	113003	179	8	CL1Contig9434	74	Sucrose synthase	B3F8H6_NICLS
74	23386	179	5	CL1Contig7201	2	Uncharacterized protein	A9PBU6_POPTR
75	37116	177	6	CL1Contig4611	17	Uncharacterized protein	A9PF58_POPTR
76	14960	175	8	FQEH0BT01B8VNA	1	Heat shock protein	Q2VU87_9ASPA
77	42537	174	6	CL1Contig8231	8	FAD-dependent oxidoreductase	Q8SA59_HELAN
78	98282	174	6	CL115Contig1	89	Coatomer alpha subunit	Q70I39_LOTJA
79	64714	174	4	CL1Contig5128	50	TCP-1/cpn60 chaperonin	Q75HJ3_ORYSJ
80	54591	173	8	CL1Contig1217	11	Heat shock protein	Q71EE1_HEVBR
81	33291	173	5	CL1Contig3924	9	Heat shock protein 70	Q39641_CUCSA
82	31951	173	5	CL1Contig2039	4	Glyceraldehyde-3 phosphate dehydrogenase	Q06AM6_9CARY
83	97879	172	5	CL1Contig10122	35	Alanyl-tRNA synthetase, mitochondrial	SYA_ARATH
84	38792	172	2	CL591Contig1	49	Uncharacterized protein	A9NXH0_PICSI
85	78915	171	7	CL184Contig2	82	Phosphoglycerate mutase	PMGI_MAIZE
86	76919	171	3	CL2917Contig1	14	Cell division cycle protein 48	Q10RP0_ORYSJ
87	15828	171	5	FQEH0BT01AP04Y	1	Class IV chitinase	Q6RV28_MEDTR
88	46839	170	5	CL1Contig231	68	Proteasome alpha subunit	A9P9A3_POPTR
89	76213	169	10	CL1Contig6978	10	Uncharacterized protein	Q9LTT8_ARATH
90	15934	169	6	FQEH0BT01EPB7M	1	Enolase	Q1X8N5_PRUAR
91	82974	168	6	CL136Contig1	50	Uncharacterized protein	A2Y3K8_ORYSI
92	19744	168	3	CL1Contig7383	15	Glyceraldehyde-3 phosphate dehydrogenase	Q06AM6_9CARY
93	59987	167	5	CL1Contig2918	42	Uncharacterized protein	A2XWD5_ORYSI
94	14408	165	4	FQEH0BT01DP88G	1	Exoglucanase	Q8W2Z8_ORYSA
95	25071	164	5	CL13Contig7	40	Predicted protein	A9SF68_PHYPA
96	25504	163	7	CL181Contig5	21	14-3-3 protein	Q9SEC9_PIMBR
97	85588	162	4	CL1Contig1118	60	Importin alpha 2	A1YUL9_NICBE
98	23919	161	10	CL33Contig4	25	Profilin	PROF_CHEAL
99	62758	161	5	CL420Contig2	17	Uncharacterized protein	Q0WM35_ARATH
100	29986	161	6	CL1Contig1178	8	Peptidyl-prolyl cis-trans isomerase	Q96416_DIGLA
101	36052	159	9	CL32Contig7	34	Beta-tubulin	B5U1R1_9ROSA
102	14071	159	7	FQEH0BT01B8N1Q	1	Malate dehydrogenase	Q9FSF0_TOBAC
103	27162	158	4	CL3Contig8	2	Uncharacterized protein	A9PJ12_POPJC
104	19075	158	8	CL1Contig923	11	Polyubiquitin	Q9M5X0_EUPES
105	27843	157	3	CL9Contig4	114	40S ribosomal S8-like protein	B6DMH1_9LAMI
106	46356	157	7	CL181Contig1	28	14-3-3 protein	14336_ARATH
107	66083	157	4	CL1Contig4799	32	DNA topoisomerase 2	Q8GSC4_TOBAC
108	53442	156	6	CL146Contig7	14	Phosphoenolpyruvate carboxylase	Q8S917_NICSY
109	15306	156	6	FQEH0BT01AP1AD	1	Uncharacterized protein	A9PDI4_POPTR
110	67172	155	5	CL83Contig3	41	Uncharacterized protein	Q9C574_ARATH
111	79404	154	3	CL323Contig1	67	Elongation factor protein	Q9LNC5_ARATH
112	25551	154	6	CL1Contig3966	46	14-3-3 protein	Q75ZD6_TOBAC

113	75372	153	6	CL1Contig2153	127	Uncharacterized protein	A9PH77_POPTR
114	25491	152	4	CL1Contig7071	5	Heat shock protein 70, chloroplastic	A2TJV6_CUCSA
115	33411	152	5	CL566Contig3	10	Trehalose synthase	B2ZAS1_9ROSI
116	41576	151	10	CL1Contig17	48	Elongation factor 1-alpha	Q9SPA1_LILLO
117	18057	151	4	CL13115Contig1	2	Cysteine synthase	Q2I306_9MAGN
118	14347	148	7	FQEH0BT01A9NIG	1	Uncharacterized protein	A9NV08_PICSI
119	17886	147	5	CL1Contig5345	2	FAD-dependent oxidoreductase	Q2HTY5_MEDTR
120	39181	147	4	CL44Contig3	66	ATP synthase beta subunit, mitochondrial	ATPBM_HEVBR
121	88924	147	8	CL1121Contig1	32	Translocase, chloroplastic	TC132_ARATH
122	26853	146	3	CL5058Contig1	8	Uncharacterized protein	Y3478_ARATH
123	43373	145	5	CL1Contig8239	61	Homocysteine methyltransferase	METE_CATRO
124	29070	145	4	CL216Contig4	7	Isocitrate dehydrogenase	Q6R6M7_PEA
125	38504	145	3	CL471Contig1	11	Aminopeptidase-like protein	Q8VZH2_ARATH
126	89233	145	4	CL57Contig1	151	Uncharacterized protein	Q0DZG1_ORYSJ
127	15491	142	3	FQEH0BT01DJQIH	1	Triosephosphate isomerase	Q6T379_SOLCH
128	18951	142	3	FQEH0BT01DYR9U	1	Alternative oxidase 2, mitochondrial	AOX2_TOBAC
129	12766	142	4	FQEH0BT01CVK38	1	Heat shock 70kDa protein	HSP7M_PHAVU
130	43750	140	3	CL1181Contig1	12	Salt tolerance protein 5	Q67X37_ORYSJ
131	82378	140	4	CL337Contig1	55	Cytosolic factor	Q2PF01_TRIPR
132	40577	139	4	CL1863Contig1	14	6-Phosphogluconolactonase	B6UAK0_MAIZE
133	15629	138	10	FQEH0BT01BELLD	1	Elongation factor 1-alpha	B6V864_PRUPE
134	48282	137	3	CL172Contig4	55	Proteasome alpha subunit	A9PCM5_POPTR
135	18226	137	4	FQEH0BT01AWC0R	1	Chitinase	Q7Y1Z0_ORYSJ
136	19521	136	3	CL1Contig7641	4	Proteasome alpha subunit	Q3HVM9_SOLTU
137	90678	135	4	CL545Contig1	36	Uncharacterized protein	Q9SYP1_ARATH
138	15454	135	5	FQEH0BT01CT8E2	1	Uncharacterized protein	A9PD55_POPTR
139	82175	135	5	CL322Contig3	62	Pyruvate kinase	Q8L7J4_SOYBN
140	16561	134	4	FQEH0BT01C13H5	1	Methionine synthase	A6YGE7_CARPA
141	13753	134	3	FQEH0BT01AVJSI	1	Enolase	Q6Q4Z3_CAPBU
142	54547	133	3	CL3Contig6	269	Protein disulfide-isomerase	PDI_RICCO
143	13942	133	3	FQEH0BT01DKXIJ	1	Importin alpha	Q2PEZ3_TRIPR
144	29960	133	3	CL1Contig1810	38	Cell division cycle protein 48 homolog A	CD48A_ARATH
145	105952	133	3	CL1Contig4420	83	Vacuolar proton-inorganic pyrophosphatase	Q8GT22_PYRCO
146	17779	132	3	FQEH0BT01BEROG	1	Cell wall invertase	Q9ZU02_FRAAN
147	67774	132	4	CL913Contig1	28	Pyruvate kinase	KPYC_TOBAC
148	18253	132	4	FQEH0BT01CYATM	1	Uncharacterized protein	B4FRN3_MAIZE
149	29569	132	3	CL1Contig8939	10	26S proteasome regulatory subunit S2	Q9SIV2_ARATH
150	45074	131	2	CL1Contig201	31	Uncharacterized protein	Q9LTT8_ARATH
151	50087	131	5	CL1Contig6704	49	Uncharacterized protein	A9P8T6_POPTR
152	32655	131	3	CL7Contig6	26	Elongation factor 1-gamma	B2LT58_TOBAC
153	35634	130	2	CL532Contig1	37	Proteasome beta subunit	Q0DAN3_ORYSJ
154	92367	130	4	CL1Contig4958	313	Calnexin	B6TNF1_MAIZE
155	13229	129	4	FQEH0BT01BSA1T	1	ADP-ribosylation factor	B3TLZ8_ELAGV
156	73900	129	4	CL1Contig6489	20	Uncharacterized protein	Q8W4F3_ARATH
157	28958	129	2	CL1681Contig2	19	Peptidyl-prolyl cis-trans isomerase	A9P2H9_PICSI
158	23216	128	3	CL1Contig9534	3	Methionyl-tRNA synthetase	SYM_ORYSJ
159	14171	128	3	FQEH0BT01AEJ5E	1	Uncharacterized protein	B7FJR8_MEDTR
160	14129	128	4	CL1Contig4179	3	Glycine-rich RNA-binding protein 3	Q9M6A0_CATRO
161	47936	128	4	CL1Contig4704	46	Actin	ACT7_ARATH
162	81608	128	5	CL98Contig5	128	Importin alpha 2	Q94KA9_CAPAN
163	31740	127	3	CL96Contig1	16	Coatomer gamma subunit	B6SV32_MAIZE
164	50011	127	4	CL1Contig8809	50	Elongation Factor EF-2	Q9ASR1_ARATH
165	46712	127	5	CL1294Contig1	11	Carbamoyl phosphate synthase large subunit	Q8L6J9_TOBAC
166	13090	126	3	FQEH0BT01DK5SE	1	Aldehyde dehydrogenase, mitochondrial	Q93XI6_HORVU
167	12934	126	2	FQEH0BT01C8KMI	1	Uncharacterized protein	B4FWI0_MAIZE
168	12092	125	3	FQEH0BT01CDWFI	1	Peroxiredoxin	Q5YJK8_HYAOR
169	12794	125	4	CL1Contig9604	2	TO23-1	Q3LVQ4_TAROF
170	17648	125	3	FQEH0BT01CRKL5	1	Uncharacterized protein	A9PF62_POPTR

171	34818	124	3	CL208Contig1	23	UDP-glucose:glycoprotein glucosyltransferase	Q0WL80_ARATH
172	26775	124	3	CL2Contig7	4	Cell wall invertase	Q9ZU02_FRAAN
173	38997	124	4	CL266Contig4	29	Uncharacterized protein	A9PCE2_POPTR
174	64860	124	2	CL420Contig5	27	Sec31p	Q94LY4_ORYSA
175	41244	123	5	CL1Contig6462	30	Methionyl-tRNA synthetase	SYM_ORYSJ
176	57394	123	3	CL1Contig2049	28	Uncharacterized protein	A9P8T6_POPTR
177	55655	123	4	CL1Contig1755	15	Heat shock 70 protein	O22664_SPIOL
178	16402	123	4	CL1Contig10402	16	Hairpin-inducing protein	Q9M663_TOBAC
179	64197	122	3	CL38Contig2	96	Glutamate synthase	GLSN_MEDSA
180	11685	121	6	FQEH0BT01CDCOB	1	ADP/ATP translocator-like	Q307Y6_SOLTU
181	39825	121	3	CL1Contig1845	54	Serine hydroxymethyltransferase	A9PL09_POPTM
182	37853	121	3	CL1298Contig1	14	Uncharacterized protein	A9PAU0_POPTR
183	51976	121	5	CL92Contig2	22	Uncharacterized protein	A9PDC7_POPTR
184	118254	121	3	CL1Contig3815	48	Dynamin-related protein 3A	DRP3A_ARATH
185	146262	120	4	CL1Contig1621	53	Expostin (XPO1) protein	Q9SMV6_ARATH
186	13632	120	3	FQEH0BT01DCU8X	1	Sal m 1 allergen	A8CVH3_SALMI
187	52873	120	5	CL1Contig4717	18	Cp10-like protein	Q8LK52_GOSHI
188	51094	119	4	CL1Contig2942	34	RuBisCo beta subunit binding-protein	Q6B7Q9_MAIZE
189	93350	119	6	CL560Contig3	31	Uncharacterized protein	Q6H4V4_ORYSJ
190	18379	119	4	FQEH0BT01DNKVD	1	Uncharacterized protein	Q0J0V1_ORYSJ
191	47039	118	4	CL1Contig1541	32	Guanine nucleotide-binding protein beta subunit	GBLP_MEDSA
192	22097	118	3	CL948Contig2	7	Uncharacterized protein	A9PDF0_POPTR
193	63469	117	3	CL289Contig2	57	Uncharacterized protein	B7FIW1_MEDTR
194	19525	117	3	CL68Contig1	23	Ribosomal protein L15	A9PC51_POPTR
195	26507	117	2	CL1Contig5222	25	Stylopine synthase	Q50LH3_ESCCA
196	35225	117	4	CL1Contig4940	10	Asparaginyl-tRNA synthetase	SYNC1_ARATH
197	73902	117	4	CL326Contig1	75	Rubisco alpha subunit binding-protein	Q2PEP1_TRIPR
198	74435	117	1	CL1Contig3008	177	RNA binding protein 45	Q9LEB4_NICPL
199	29214	116	2	CL1Contig4218	10	Small heat shock protein, mitochondrial	O80432_SOLLC
200	38156	116	3	CL556Contig4	21	Uncharacterized protein	Q9SFF5_ARATH
201	11219	116	2	FQEH0BT01ETAZY	1	Uncharacterized protein	B3H477_ARATH
202	14631	115	4	FQEH0BT01AJ0JH	1	Glutamate synthase	Q9ZNX7_ORYSA
203	16713	115	4	CL1Contig5790	2	Uncharacterized protein	O04428_CITPA
204	44174	115	4	CL58Contig2	95	Uncharacterized protein	B7FGT0_MEDTR
205	17393	115	6	FQEH0BT01BID2W	1	Sucrose synthase	Q00P15_EUCGR
206	15942	115	3	CL362Contig1	2	Csf-1 protein	Q9SXL9_CUCSA
207	95715	114	6	CL1Contig6764	37	Valyl-tRNA synthetase	SYV_ARATH
208	15885	114	2	FQEH0BT01COFWZ	1	Luminal-binding protein	Q9FSY7_CORAV
209	27061	114	3	CL1Contig8273	4	Uncharacterized protein	A9NKY2_PICSI
210	31588	114	3	CL1Contig3030	64	Ribosomal protein S25	B0FSK2_9MAGN
211	70742	114	3	CL149Contig4	71	Uncharacterized protein	A9PCK1_POPTR
212	24990	113	4	CL357Contig2	7	Pathogenesis-related protein PR10A	Q7Y083_DATGL
213	28145	113	2	CL3951Contig1	7	Uncharacterized protein	A9PBT3_POPTR
214	18671	113	3	CL1Contig9390	2	Uncharacterized protein	A9PAK3_POPTR
215	13704	113	2	FQEH0BT01ENIU3	1	Heat shock 22 kDa protein, mitochondrial	HS22M_SOYBN
216	28349	113	3	CL1Contig4444	14	Pathogenesis-related protein STH-2	Q53U35_SOLLC
217	24218	112	3	CL13Contig5	2	Disease resistance-responsive protein	Q45W75_ARAHY
218	30070	112	3	CL1Contig7503	6	Uncharacterized protein	A9PCY9_POPTR
219	37028	112	6	CL1Contig5327	34	Uncharacterized protein	A9PD55_POPTR
220	24977	111	3	CL3622Contig1	6	Heat shock protein	Q9SIF2_ARATH
221	38583	111	2	CL1Contig7300	48	Ribosomal protein S3	A0N0S0_9ASPA
222	40550	111	3	CL3622Contig2	5	Uncharacterized protein	Q0JZ5_ORYSJ
223	10468	111	3	FQEH0BT01B5W7L	1	Uncharacterized protein	Q67W57_ORYSJ
224	15117	111	3	FQEH0BT01A9YN8	1	Peroxidase 52	PER52_ARATH
225	80934	111	2	CL472Contig1	58	Uncharacterized protein	A9PHT1_POPTR
226	40113	111	3	CL1685Contig1	14	Uncharacterized protein	B7FN18_MEDTR
227	81623	110	4	CL412Contig6	32	Karyopherin-beta 3 variant	Q6ZL37_ORYSJ
228	83820	110	5	CL385Contig1	62	Monocopper oxidase	SKU5_ARATH

229	29677	110	2	CL471Contig3	18	Aminopeptidase-like protein	Q8VZH2_ARATH
230	40623	110	2	CL493Contig2	20	Uncharacterized protein	B7FIU2_MEDTR
231	27146	109	3	CL1Contig1775	15	Glutathione *S*-transferase 3	Q9SEK1_PAPSO
232	46685	109	2	CL1Contig647	25	Signal peptidase S26A	A2Q5F8_MEDTR
233	16867	109	3	FQEH0BT01C3K4M	1	Plasma membrane-associated AAA-ATPase	Q2HZ34_SOYBN
234	66807	109	2	CL1455Contig1	18	Transportin-like protein	Q8GTE6_CICAR
235	57703	109	4	CL1Contig1766	30	Uncharacterized protein	O22957_ARATH
236	64568	109	4	CL1Contig9641	32	Plasma membrane proton ATPase	Q9ARG5_LILLO
237	12048	109	1	FQEH0BT01CVCHH	1	Uncharacterized protein	A9PIJ1_POPTR
238	16259	108	2	FQEH0BT01C0I0G	1	40S ribosomal protein S19	B3TLL5_ELAGV
239	70930	108	2	CL1Contig640	185	Homocysteine methyltransferase	METE_CATRO
240	65726	108	3	CL208Contig4	40	UDP-glucose:glycoprotein glucosyltransferase	Q0WL80_ARATH
241	74943	108	2	CL1Contig5993	16	Calcium-transporting ATPase 1	ECA1_ARATH
242	22157	108	1	CL83Contig9	9	Uncharacterized protein	Q9C574_ARATH
243	38702	107	4	CL283Contig2	20	Proteasome alpha subunit	PSA6_SOYBN
244	18112	107	5	CL1Contig7807	2	Heat shock protein 70	Q9M4E6_CUCSA
245	18095	107	2	FQEH0BT01B33ZY	1	Glutathione *S*-transferase	Q84VH3_MALPU
246	21106	107	2	CL1Contig9591	9	Uncharacterized protein	B7FN58_MEDTR
247	109340	107	1	CL114Contig5	70	Uncharacterized protein	A9P896_POPTR
248	17354	106	3	CL2Contig6	6	Cell wall invertase	Q9ZP42_FRAAN
249	17871	106	3	CL1Contig5551	4	Nectarin IV	Q3KU27_NICLS
250	50154	106	5	CL1Contig2433	67	14-3-3 protein	Q9LKK9_9ROSI
251	19644	106	2	CL1Contig6194	6	Methionine synthase	A6YGE7_CARPA
252	82303	105	2	CL309Contig2	68	Formate-tetrahydrofolate ligase	FTHS_ARATH
253	18435	105	3	CL1Contig5037	2	Elongation Factor EF-2	Q9SGT4_ARATH
254	79748	105	2	CL44Contig1	168	ATP synthase beta subunit	Q0DG48_ORYSJ
255	44399	104	2	CL1Contig2874	24	26S proteasome regulatory subunit 6	B4FVL1_MAIZE
256	39589	104	6	CL238Contig3	29	14-3-3-like protein	A1XJ43_GOSHI
257	18810	104	2	CL1Contig6314	2	Ribosomal protein S3	A0N0S0_9ASPA
258	18926	104	2	CL105Contig1	18	60S ribosomal protein L38	RL38_ARATH
259	28643	104	2	CL6447Contig1	6	Nuclear-pore anchor	A4GSN8_ARATH
260	91298	104	3	CL410Contig1	61	Methionine *S*-methyltransferase	MMT1_WOLBI
261	14389	104	3	FQEH0BT01EPR9H	1	60S acidic ribosomal protein	Q8H2B9_PRUDU
262	19742	103	1	CL6114Contig1	5	Uncharacterized protein	A9PFW2_POPTR
263	89122	103	3	CL59Contig6	47	Uncharacterized protein	Q9SUZ1_ARATH
264	26258	103	2	CL23Contig7	30	Uncharacterized protein	Q38HT5_SOLTU
265	17185	103	3	FQEH0BT01DYNYD	1	Luminal-binding protein	Q9FSY7_CORAV
266	27875	103	4	CL1Contig4447	3	Polyphenol oxidase	Q6YHK5_ANACO
267	102730	103	5	CL249Contig3	47	Tudor domain protein	Q8L5C2_PEA
268	53534	103	4	CL1Contig7048	42	Thioredoxin reductase	Q0WW76_ARATH
269	43439	103	2	CL1Contig5141	42	Fructose bisphosphate aldolase	A9PEU6_POPTR
270	62645	103	2	CL584Contig3	46	UTP-glucose 1 phosphate uridylyltransferase	B3VDY8_EUCGR
271	13561	102	2	FQEH0BT01BR4N6	1	RNA polymerase	B3TLS5_ELAGV
272	44400	102	1	CL2091Contig2	14	Proliferating cell nuclear antigen	PCNA_TOBAC
273	14504	102	3	FQEH0BT01DZAS2	1	Ribosomal protein L30e	A7X6P5_PEA
274	17339	102	3	FQEH0BT01AUUUC	1	GTP-binding nuclear protein RAN	Q6J9G2_NICSY
275	93916	102	2	CL358Contig3	45	CM0545.290.nc protein	B0BLB2_LOTJA
276	58324	101	1	CL539Contig3	18	Phenylalanyl-tRNA synthetase beta chain	SYFB_ARATH
277	14631	101	2	CL44Contig2	2	ATP synthase beta subunit	O04275_PEA
278	16631	101	2	CL1Contig249	6	60S ribosomal protein L37a	Q5GMM4_CAPCH
279	88298	101	4	CL147Contig1	113	AAA ATPase; 26S proteasome subunit P45	Q2HTA3_MEDTR
280	65982	100	3	CL1Contig5526	37	Glutamate decarboxylase	O81102_TOBAC
281	26831	100	4	CL1Contig5388	3	60S acidic ribosomal protein	RLA0_SOYBN
282	14195	100	4	FQEH0BT01A3EWI	1	Uncharacterized protein	A2XX38_ORYSI
283	31468	100	3	CL603Contig3	6	E3 ubiquitin-protein ligase UPL3	UPL3_ARATH
284	33947	100	5	CL1Contig10188	5	Bacterial-induced peroxidase	Q9XGV6_GOSHI
285	86951	100	3	CL331Contig2	66	V-type proton ATPase catalytic subunit A	VATA_DAUCA
286	13997	99	4	FQEH0BT01CJ5XD	1	FAD-dependent oxidoreductase	O64743_ARATH

287	30104	99	1	CL1Contig7274	18	Isocitrate dehydrogenase subunit 5, mitochondrial	IDH5_ARATH
288	59513	99	2	CL200Contig3	40	26S proteasome regulatory subunit 3	PSMD3_DAUCA
289	15354	99	3	CL1Contig9845	4	Malate dehydrogenase	Q9FT00_CICAR
290	13715	99	3	CL71Contig7	2	Uncharacterized protein	A9P8Z9_POPTR
291	39248	99	1	CL1Contig1294	106	Phosphoglycerate kinase	Q9SAJ4_ARATH
292	15611	98	2	FQEH0BT01DOAO4	1	Malic enzyme	Q9FRT2_ORYSJ
293	97995	98	2	CL815Contig1	39	Uncharacterized protein	A3A338_ORYSJ
294	15741	98	5	CL1Contig122	2	14-3-3 protein	Q9LKL0_9ROSI
295	78472	98	4	CL252Contig1	29	Uncharacterized protein	A3BE19_ORYSJ
296	48806	97	5	CL1Contig4729	31	Guanine nucleotide-binding protein beta subunit	GBLP_MEDSA
297	28838	97	3	CL606Contig2	30	Nucleoside diphosphate kinase 1	NDK1_ARATH
298	16672	97	4	FQEH0BT01EZM0L	1	Ribosomal L11-like protein	Q307Y8_SOLTU
299	13715	97	2	FQEH0BT01B5VAR	1	Fructose bisphosphate aldolase	Q9SJQ9_ARATH
300	20541	97	2	CL1Contig3665	26	Proteasome alpha subunit	A9PAG0_POPTR
301	30559	97	3	CL1Contig3270	5	RNA-binding gricine-rich protein-1c	Q40425_NICSY
302	39289	97	3	CL3050Contig1	13	GTP-binding protein	Q6DXU0_GOSHI
303	32353	97	3	CL2925Contig2	6	MCM-like protein	Q8H0G9_TOBAC
304	14757	97	3	FQEH0BT01C7SQB	1	Casein kinase II regulatory subunit	B3VIM8_POPTN
305	20797	96	2	CL118Contig3	4	Diaminopimelate decarboxylase 2	DCDA2_ARATH
306	41184	96	3	CL1Contig1496	16	Uncharacterized protein	B7FH81_MEDTR
307	20992	96	3	CL639Contig6	5	Uncharacterized protein	Q8L640_ARATH
308	34025	96	4	CL146Contig1	27	Phosphoenolpyruvate carboxylase	A6YM34_RICCO
309	51151	95	2	CL187Contig2	21	T-complex protein 1, alpha subunit	Q8H9B2_9ROSI
310	24869	95	3	CL1Contig7961	7	40S ribosomal protein S15a	RS15A_DAUCA
311	22787	95	4	CL1Contig9378	11	Citrate synthase	Q946X8_PRUPE
312	76796	95	2	CL592Contig2	35	Glutathione reductase	GSHRP_TOBAC
313	14717	95	3	FQEH0BT01CMJ33	1	Malate dehydrogenase	Q5DNZ8_TOBAC
314	31091	95	3	CL1Contig4949	49	Uncharacterized protein	B7FN54_MEDTR
315	18860	95	1	CL3936Contig2	3	dTDP-D-glucose 4,6-dehydratase-like	Q6EPQ1_ORYSJ
316	68904	95	3	CL1Contig7747	62	Asparaginyl-tRNA synthetase 1	SYNC1_ARATH
317	16083	94	1	CL155Contig3	2	Fructokinase	Q42645_BETVU
318	86980	94	3	CL1Contig2410	28	2-Oxoglutarate dehydrogenase E1 subunit	Q84VW8_ARATH
319	19805	94	2	CL98Contig6	2	Uncharacterized protein	Q0JP03_ORYSJ
320	81757	94	2	CL1Contig3176	297	ADP/ATP translocator-like protein	Q307Y6_SOLTU
321	41580	94	1	CL1Contig4193	7	Uncharacterized protein	Q8L7N9_ARATH
322	25028	94	1	CL1932Contig2	8	Pyruvate kinase isozyme G	KPYG_RICCO
323	30627	94	2	CL940Contig2	5	Uncharacterized protein	A9PJK5_POPJC
324	41263	94	2	CL318Contig8	6	Aldehyde oxidase	Q9FV23_SOLLC
325	27529	94	1	CL200Contig1	29	Cysteine synthase	Q8W1A0_SOYBN
326	73268	94	2	CL1Contig10323	88	Polyphenol oxidase	PPO_MALDO
327	57746	93	2	CL1Contig6962	58	Glycyl-tRNA synthetase 1	SYGM1_ARATH
328	35481	93	1	CL3035Contig1	7	Putative importin 7	Q6K6G8_ORYSJ
329	70590	93	3	CL1Contig8423	35	26S proteasome regulatory subunit S2	Q9SIV2_ARATH
330	16619	93	3	FQEH0BT01D694C	1	Protein disulfide isomerase	PDI_DATGL
331	17164	93	3	CL67Contig4	2	6-Phosphogluconate dehydrogenase	Q81238_MAIZE
332	10493	93	2	FQEH0BT01CBHQQ	1	Small GTPase Rab2	Q946G3_TOBAC
333	15306	93	3	CL1Contig3821	2	60S ribosomal protein L18-3	RL183_ARATH
334	23058	93	2	CL1Contig9780	2	Uncharacterized protein	A9P8J6_POPTR
335	55442	92	4	CL1Contig2504	241	Guanine nucleotide-binding protein beta subunit	GBLP_MEDSA
336	20350	92	3	CL9172Contig1	4	Malate dehydrogenase	Q5QLS8_ORYSJ
337	99210	92	3	CL1365Contig1	28	Glutamate synthase	Q7M242_TOBAC
338	22048	92	2	CL331Contig1	7	V-type proton ATPase catalytic subunit A	VATA_BRANA
339	37558	92	4	CL1Contig2679	43	60S ribosomal protein L8-1	RL81_ARATH
340	12590	92	1	FQEH0BT01C6218	1	Nuclear pore complex protein Nup155	Q5Z4E0_ORYSJ
341	16247	92	1	CL1Contig1739	3	Fructose 6-phosphate 1-phosphotransferase beta	PFPB_SOLTU
342	11518	91	1	CL13970Contig1	2	Malonyltransferase	Q2VA66_SOYBN
343	49068	91	2	CL360Contig4	34	Uncharacterized protein	A9NT13_PICSI
344	14928	91	2	FQEH0BT01CMS3W	1	Uncharacterized protein	A9PF62_POPTR

345	44427	91	2	CL1Contig2904	12	DNA topoisomerase 2	Q2L363_MALDO
346	32230	90	2	CL302Contig4	20	Plastid acyl carrier protein	B3GQB9_9ERIC
347	62451	90	1	CL999Contig1	35	Serine carboxypeptidase-like 27	SCP27_ARATH
348	104768	90	3	CL1Contig8761	37	Glyoxysomal beta-oxidation multifunctional protein	B1Q485_CAPCH
349	90424	90	2	CL2Contig10	60	ATP-dependent RNA helicase	DHX15_ARATH
350	120033	90	4	CL426Contig4	42	Uncharacterized protein	A2Z0N8_ORYSI
351	17445	90	2	FQEH0BT01DG931	1	Cell division control protein 48 homolog D	CD48D_ARATH
352	29475	89	2	CL2212Contig2	11	GTP-binding nuclear protein ras-like	Q9FJH0_ARATH
353	31657	89	4	CL1Contig5393	59	ATP synthase alpha subunit	Q7HJM1_BETVU
354	33992	89	3	CL1Contig536	92	Ran binding protein-1	Q94K24_SOLLC
355	38018	89	2	CL1172Contig1	32	Translocon-associated protein beta	Q2VCI0_SOLTU
356	25580	89	1	CL1Contig5311	3	Poly(A)-binding protein	Q9M549_CUCSA
357	5749	89	2	CL1Contig1374	6	Alpha-tubulin	Q39719_EUCGL
358	54007	89	1	CL194Contig3	53	TGF-beta receptor-interacting protein 1	Q94KS2_PHAVU
359	50713	89	2	CL253Contig1	34	NADPH:quinone oxidoreductase	NQR_ARATH
360	17054	88	2	CL1131Contig1	9	Uncharacterized protein	B7FNA2_MEDTR
361	21003	88	2	CL183Contig6	2	Uncharacterized protein	Q5NBT9_ORYSJ
362	45646	88	3	CL1Contig4314	53	Uncharacterized protein	A9NMF2_PICSI
363	13594	88	2	FQEH0BT01EBRWQ	1	Enolase	ENO_SOLLC
364	29120	88	1	CL3477Contig1	5	ADP-ribosylation factor-like protein	Q93Y31_ARATH
365	44667	88	1	CL52Contig3	40	Hypersensitive-induced response protein	Q9FM19_ARATH
366	53531	88	3	CL1007Contig2	28	Alpha-1,4-glucan protein synthase (UDP-forming)	Q2HV87_MEDTR
367	35897	88	1	CL1Contig10307	21	Substrate carrier, mitochondrial	Q2HUK6_MEDTR
368	33005	88	3	CL4749Contig1	8	Glutamate synthase	Q9LV03_ARATH
369	21005	87	3	CL1Contig9151	4	Uncharacterized protein	A9P9J0_POPTR
370	69798	87	2	CL64Contig1	141	Fumarate hydratase 1, mitochondrial	Q10LR5_ORYSJ
371	19343	87	3	CL8420Contig1	4	Phosphoenolpyruvate carboxylase	Q9FV65_FLATR
372	39225	87	2	CL807Contig2	18	Uncharacterized protein	A9PHH1_POPTR
373	11300	87	1	FQEH0BT01A3IF4	1	Enolase	ENO_ALNGL
374	56185	87	2	CL151Contig4	56	26S proteasome AAA-ATPase subunit RPT4a	Q8H9D4_SOLTU
375	87368	87	2	CL457Contig2	51	Leucine zipper transmembrane protein 1	Q6K1Y0_ORYSJ
376	44274	87	3	CL115Contig3	11	Uncharacterized protein	B7EQ85_ORYSJ
377	44556	87	1	CL381Contig1	51	Acyl-coenzyme A oxidase	ACO32_ARATH
378	37314	87	2	CL4487Contig1	9	Peroxidase 2b	B2G335_CATRO
379	15761	86	1	CL1Contig1386	2	Fungal endoglucanase inhibitor	Q8GT67_SOLLC
380	64575	86	1	CL2008Contig1	18	Uncharacterized protein	Q9AYJ9_ORYSA
381	20485	86	2	CL2247Contig2	4	Uncharacterized protein	A3B6T9_ORYSJ
382	43513	86	2	CL1Contig7799	11	Glutamate decarboxylase	O81102_TOBAC
383	33447	86	2	CL9Contig7	22	40S ribosomal protein S8	B3TM36_ELAGV
384	26922	86	1	CL1206Contig2	6	AP-1 complex gamma subunit 1	B6SV75_MAIZE
385	17375	86	2	CL590Contig2	2	*N*-acetyl-gamma-glutamyl-phosphate reductase	A9SPM8_PHYPA
386	76841	86	2	CL1285Contig1	25	Mini-chromosome maintenance protein MCM3	Q8H1A2_PEA
387	16635	86	1	FQEH0BT01AEZ0C	1	Profilin	PROF_CHEAL
388	11629	85	2	FQEH0BT01AS2TK	1	Uncharacterized protein	B7FJ04_MEDTR
389	21353	85	4	CL76Contig1	2	GDP-dissociation inhibitor	A7Y7F0_LUPAL
390	14677	85	2	FQEH0BT01AT3YF	1	Proteasome alpha subunit alpha	A9PJR2_POPJC
391	9483	85	1	FQEH0BT01CKZX1	1	Nectarin IV	Q3KU27_NICLS
392	19803	85	1	CL475Contig4	8	26S proteasome regulatory subunit 11	B6TWL4_MAIZE
393	15964	85	4	FQEH0BT01EI74T	1	Heat shock protein 90	Q9M555_EUPES
394	54488	85	3	CL1Contig7450	38	JPR ORF1 protein	Q9ST36_PYRPY
395	137463	85	2	CL1Contig8422	75	AGO1-1	Q2LFC4_NICBE
396	15025	84	3	FQEH0BT01B52FH	1	Putative aconitate hydratase	Q0DCP8_ORYSJ
397	14432	84	2	FQEH0BT01B5WFH	1	Tetrahydroprotoberberine-*N*-methyltransferase	Q108P1_PAPSO
398	23143	84	3	CL1Contig10558	5	Xyloglucanase inhibitor	Q7XJE7_SOLTU
399	14704	84	3	FQEH0BT01C4S8K	1	Csf-1 protein	Q9SXL9_CUCSA
400	40650	84	3	CL1Contig6759	8	Codeinone reductase	Q9SQ67_PAPSO
401	16607	84	2	FQEH0BT01D7VMY	1	Polyphenol oxidase	B2BX66_TAROF
402	39050	84	1	CL230Contig2	7	26S proteasome regulatory particle subunit 12	Q0GEA1_CAMSI

403	121057	84	4	CL1Contig683	134	Aconitase	B1Q486_CAPCH
404	51800	84	2	CL1031Contig2	21	L-ascorbate oxidase	ASO_TOBAC
405	71120	84	2	CL2028Contig1	19	Seed imbibition protein-like	Q8RX87_ARATH
406	34925	83	3	CL250Contig3	18	Uncharacterized protein	B4FJM2_MAIZE
407	30030	83	3	CL217Contig4	20	Nucleoside diphosphate kinase 3	B7FR80_PHATR
408	16419	83	2	FQEH0BT01AJ5I6	1	Nectarin IV	Q3KU27_NICLS
409	64107	83	2	CL425Contig2	39	Glyceraldehyde-3 phosphate dehydrogenase	Q8VWN9_CAPAN
410	25248	83	2	CL92Contig5	50	Uncharacterized protein	A9PDC7_POPTR
411	10448	83	1	FQEH0BT01B173T	1	Uncharacterized protein	A9PBF5_POPTR
412	16327	83	1	CL25Contig1	3	Uncharacterized protein	A9P7V5_POPTR
413	15607	83	1	FQEH0BT01D4UED	1	Adenosine kinase	Q9XGC6_MAIZE
414	62357	83	2	CL1040Contig1	35	Uncharacterized protein	Q94F09_ARATH
415	15461	83	2	FQEH0BT01DT06M	1	Uncharacterized protein	B7FF72_MEDTR
416	53852	83	1	CL155Contig1	50	Fructokinase	Q42645_BETVU
417	38797	83	2	CL280Contig2	22	Peptidyl-prolyl cis-trans isomerase	Q75M32_ORYSJ
418	16668	83	1	CL17687Contig1	2	RNA helicase	Q48534_ARATH
419	130899	82	1	CL1Contig2336	213	Trans-cinnamate 4-hydroxylase	Q0Q5Z5_POPTM
420	54253	82	1	CL449Contig1	59	Dihydrolipoyl dehydrogenase	Q9FEN7_SOLTU
421	36227	82	2	CL556Contig6	10	Hypothetical salt-inducible protein	Q2Z1Z1_PRUMU
422	37727	82	1	CL1651Contig1	5	Microtubule bundling polypeptide TMBP200	Q8S912_TOBAC
423	35381	82	2	CL5059Contig1	8	Uncharacterized protein	A3A6T0_ORYSJ
424	13531	82	1	FQEH0BT01BGJAY	1	Homocysteine methyltransferase	METE_CATRO
425	28255	82	2	CL1Contig9893	3	40S ribosomal protein S15	B3TLS1_ELAGV
426	40868	82	1	CL1Contig5287	43	Ribosomal protein S6-like protein	Q3HVK4_SOLTU
427	14684	81	2	CL1Contig9421	6	Uncharacterized protein	A9NJY4_PICSI
428	16406	81	2	CL1Contig7691	5	Sucrose synthase 1	SUS1_TULGE
429	62245	81	1	CL1401Contig1	27	Glutamate dehydrogenase 2	A7YVW4_ACTCH
430	14059	81	2	CL1Contig8254	2	Uncharacterized protein	A9PDF8_POPTR
431	16567	81	2	CL1Contig4805	2	Uncharacterized protein	B7FKR5_MEDTR
432	12677	81	2	CL704Contig1	2	Peptidyl-prolyl cis-trans isomerase	PIN1_MALDO
433	26715	81	2	CL1Contig5158	6	Aspartate aminotransferase	Q2V3D0_ARATH
434	54053	81	2	CL1Contig6995	12	Serine/threonine-protein phosphatase BSL1	BSL1_ARATH
435	65833	80	1	CL1Contig2382	53	Glutamine synthase	Q3L0T9_CUCME
436	33407	80	3	CL947Contig3	7	Phosphoserine aminotransferase	Q0WPU8_ARATH
437	51955	80	3	CL1551Contig1	24	Uncharacterized protein	A9PCS2_POPTR
438	107982	80	2	CL247Contig2	7	Phosphoserine aminotransferase	Q0WPU8_ARATH
439	112598	80	5	CL1Contig9332	293	SHEPHERD	Q8RVG8_ARATH
440	24585	80	1	CL1Contig1559	3	Pre-mRNA splicing factor	Q8W3F7_ORYSJ
441	78227	80	2	CL353Contig2	67	Uncharacterized protein	A9NX07_PICSI
442	34978	80	1	CL806Contig2	30	Uncharacterized protein	A9P023_PICSI
443	12909	80	1	CL7145Contig1	2	Signal recognition particle 9kDa protein	Q652F6_ORYSJ
444	19225	80	1	FQEH0BT01AV8M1	1	Norcoclaurine synthase 2	Q4QTJ1_PAPSO
445	16941	80	2	FQEH0BT01A9V7L	1	Uncharacterized protein	B7FLX9_MEDTR
446	46608	80	1	CL595Contig1	40	14-3-3-like protein	Q9T0N0_PEA
447	46596	80	3	CL1Contig8538	45	Glutathion *S*-transferase	Q9SEK1_PAPSO
448	34849	80	1	CL1Contig1552	55	QM-like protein	Q9FUN3_ELAGV
449	35327	80	3	CL1Contig6612	31	Elongation factor 1-delta	EF1D_PIMBR
450	12585	80	1	FQEH0BT01C9FOX	1	DnaJ-like protein	Q9M7M2_SOLLC
451	90389	79	3	CL424Contig1	34	Spliceosomal-like protein	Q9LD60_ARATH
452	26055	79	1	CL1Contig2375	24	Uncharacterized protein	A9P9S1_POPTR
453	55322	79	2	CL1Contig2807	25	Uncharacterized protein	A9NN70_PICSI
454	33145	79	3	CL13Contig4	88	Dirigent-like protein pDIR12	Q27JA0_PICGL
455	45282	79	1	CL132Contig8	18	Chromosomal structural maintenance protein 4	SMC4_ARATH
456	25184	79	2	CL9653Contig1	3	Pectinesterase	Q9FY03_9ROSI
457	17399	79	3	CL1Contig1697	2	Glutathione *S*-transferase	Q5CCP3_9ROSI
458	37795	79	3	CL1Contig5806	8	Pyruvate kinase	A9P7U5_POPTR
459	33776	79	1	CL1335Contig1	8	THO complex 2	Q5JLC8_ORYSJ
460	97998	79	2	CL157Contig4	48	Os08g0162100 protein	Q0J7U6_ORYSJ

461	45337	79	3	CL932Contig1	38	Eugenol synthase 2	B2WSN0_CLABR
462	64493	79	2	CL1Contig10255	72	Peptidyl-prolyl isomerase	B6TI78_MAIZE
463	44482	78	1	CL132Contig7	39	6-phosphogluconate dehydrogenase	Q9FFR3_ARATH
464	15558	78	2	CL4951Contig1	3	125 kDa kinesin-related protein	K125_TOBAC
465	20971	78	2	CL3881Contig1	6	Uncharacterized protein	B4F8G4_MAIZE
466	16814	78	1	CL1497Contig2	4	Ferritin-3	FRI3_SOYBN
467	55983	78	1	CL136Contig3	54	Uncharacterized protein	Q0WML1_ARATH
468	19666	78	2	CL471Contig4	2	Aminopeptidase-like protein	Q8VZH2_ARATH
469	58580	78	1	CL658Contig3	25	Inner envelope protein, chloroplastic	Q8LPG5_ARATH
470	51643	78	2	CL154Contig2	18	UDP-glucose dehydrogenase	Q6RK07_9MAGN
471	38202	78	2	CL1Contig3696	43	Ribosomal protein S6-like protein	Q3HVK4_SOLTU
472	9256	78	1	FQEH0BT01B4ZV8	1	Phosphoribosylformylglycinamidine synthase	A9Y5J1_ARATH
473	38516	78	1	CL420Contig4	6	Uncharacterized protein	Q8L611_ARATH
474	48944	78	2	CL1Contig6360	133	Proteasome alpha subunit	Q93X34_TOBAC
475	17885	78	3	FQEH0BT01BWYOB	1	Grp94	Q8H6B6_9LILI
476	15604	77	2	CL1Contig10181	4	Serine/threonine protein phosphatase	A2Q340_MEDTR
477	26978	77	2	CL1Contig5121	6	Heat shock cognate protein 70	Q8GSN3_CUCMA
478	20747	77	2	CL172Contig3	23	Proteasome alpha subunit	A9PCM5_POPTR
479	53914	77	2	CL1294Contig2	19	Carbamoyl phosphate synthetase large chain	Q42601_ARATH
480	15053	77	3	FQEH0BT01AJN0O	1	Thioredoxin h	Q93X24_PEA
481	68159	77	3	CL1Contig4264	96	Ubiquitin-activating enzyme E1	P93393_TOBAC
482	40760	77	3	CL8697Contig1	4	Uncharacterized protein	A3A293_ORYSJ
483	48371	77	2	CL1Contig924	33	Proteasome alpha subunit 5	PSA5_SOYBN
484	36704	77	1	CL1Contig9118	5	Uncharacterized protein	A3BYP7_ORYSJ
485	17910	76	3	FQEH0BT01BNPZ1	1	Uncharacterized protein	A9PEN9_POPTR
486	20552	76	2	CL1Contig4626	3	Adenosylhomocysteinase	A9PIA2_POPTR
487	14888	76	1	FQEH0BT01DI2NL	1	Uncharacterized protein	A3A343_ORYSJ
488	17532	76	1	FQEH0BT01CD0GX	1	Uncharacterized protein	O64720_ARATH
489	16778	76	1	CL1Contig2077	7	ATP synthase alpha subunit	Q7HJM1_BETVU
490	72678	76	1	CL121Contig2	123	Glutamine synthase	GLNA1_MEDSA
491	42139	76	1	CL1932Contig1	10	Pyruvate kinase	O53WS7_TOBAC
492	39944	76	1	CL4027Contig1	10	Chlroplastic small heat shock protein	HS23C_CHERU
493	111896	76	3	CL10Contig10	81	Pleiotropic drug resistance protein 1	PDR1_TOBAC
494	18752	76	2	CL819Contig2	5	Uncharacterized protein	A9PCQ2_POPTR
495	14693	75	1	FQEH0BT01AP6UZ	1	Vacuolar ATP synthase subunit E	B3TLU2_ELAGV
496	30765	75	1	CL1Contig8072	40	Uncharacterized protein	A9PAJ5_POPTR
497	17844	75	1	CL1554Contig1	4	Uncharacterized protein	B7S4G1_PHATR
498	56307	75	1	CL1366Contig1	27	Beta-ketoacyl-ACP synthase, chloroplastic	Q41135_RICCO
499	19143	75	1	CL1Contig5799	2	Serine hydroxymethyltransferase	A9PL11_POPTM
500	54449	75	2	CL1Contig9621	116	Malate dehydrogenase	Q9SPB8_SOYBN
501	140845	75	3	CL1Contig571	55	Uncharacterized protein	A2XXG5_ORYSI
502	27314	75	2	CL631Contig1	20	Small nuclear ribonucleoprotein Sm D1	B6TXH2_MAIZE
503	84803	75	1	CL1Contig4067	94	Malic enzyme	Q9ZWJ4_ALOAR
504	15749	75	2	FQEH0BT01A3UR3	1	3,5-Epimerase/4-reductase	Q9LQ04_ARATH
505	21577	74	5	CL1Contig7378	3	Uncharacterized protein	B7FH64_MEDTR
506	15967	74	1	CL1Contig8366	2	Triosephosphate isomerase	TPIS_ARATH
507	12974	74	2	CL1Contig1864	6	Coclaurine *N*-methyltransferase	Q7XB08_PAPSO
508	11000	74	2	FQEH0BT01BUSC2	1	Actin	A4UTT6_9POAL
509	41370	74	1	CL483Contig3	14	Uncharacterized protein	A9P7U1_POPTR
510	52591	74	3	CL844Contig4	13	Arginyl-tRNA synthetase	Q7DLG4_ARATH
511	55163	74	2	CL658Contig2	15	Inner envelope protein, chloroplastic	Q24293_PEA
512	18316	74	1	CL1Contig6603	18	Triosephosphate isomerase	TPIC_SPIOL
513	22396	74	2	CL1Contig8289	2	Peroxidase 1	B5U1R3_LITCN
514	65658	74	1	CL404Contig2	31	Mitochondrial-processing peptidase alpha subunit 2	MPPA2_ARATH
515	2157	74	2	FQEH0BT01DUI71	1	ATP/ADP translocator, chloroplastic	A4UTR8_PEA
516	72854	73	1	CL156Contig3	97	Mitochondrial-processing peptidase beta subunit	Q9AXQ2_CUCME
517	45416	73	1	CL12807Contig1	3	ATPase 8, plasma membrane-type	PMA8_ARATH
518	18445	73	2	CL1Contig8142	9	60S ribosomal protein L23A	RL23A_FRIAG

519	61332	73	3	CL1Contig6135	42	Eukaryotic translation initiation factor 3 subunit A	EIF3A_ARATH
520	53803	73	1	CL696Contig1	33	Uncharacterized protein	A9PGY1_POPTR
521	42083	73	1	CL103Contig3	22	Uncharacterized protein	Q94K62_ARATH
522	18948	73	4	FQEH0BT01A9SU6	1	Uncharacterized protein	A9TT23_PHYPA
523	114341	73	2	CL1Contig7502	46	Eukaryotic translation initiation factor 3 subunit A	EIF3A_TOBAC
524	71999	73	1	CL154Contig1	57	UDP-glucose dehydrogenase 1	Q6IVK7_TOBAC
525	30583	73	1	CL1Contig4312	7	Argonaute 1	A1E5M3_PEA
526	15823	73	3	CL238Contig2	4	Uncharacterized protein	A9PBC6_POPTR
527	17226	73	1	FQEH0BT01DJOT4	1	Adenosylhomocysteinase	Q5D6C5_ARATH
528	18166	73	2	CL9873Contig1	3	Peroxidase 2b	B2G335_CATRO
529	27178	73	2	CL3Contig7	3	Patellin-5	PATL5_ARATH
530	29704	73	2	CL1Contig762	31	Heat shock cognate protein 70	Q8GSN4_CUCMA
531	15443	73	1	CL1Contig5615	3	Adenosylhomocysteinase	Q84VE1_ORYSJ
532	13742	73	3	FQEH0BT01CRWCX	1	Karyopherin-beta 3 variant	Q6ZL37_ORYSJ
533	17717	72	2	CL8688Contig1	4	No Hit	
534	37399	72	2	CL2627Contig2	14	PII protein	Q9ZST5_RICCO
535	45213	72	2	CL287Contig4	36	Uncharacterized protein	A9PA39_POPTR
536	59662	72	1	CL1Contig6383	25	Adapter-related protein complex 1 beta subunit	Q10LA0_ORYSJ
537	16250	72	1	FQEH0BT01BSY2Z	1	Seed calcium dependent protein kinase a	Q84P29_SOYBN
538	16697	72	2	CL1Contig1576	5	Uncharacterized protein	Q0JDN5_ORYSJ
539	17275	72	2	FQEH0BT01AP0KV	1	Uncharacterized protein	A9PGV8_POPTR
540	16918	72	2	FQEH0BT01CJXN2	1	Uncharacterized protein	A9PGQ6_POPTR
541	22851	72	1	CL1207Contig1	10	Auxin resistant 1	A8IXR9_BRACM
542	19774	72	1	CL1Contig5090	2	3'-Hydroxy-*N*-methylcoclaurine 4'-*O*-methyltransfera	Q7XB10_PAPSO
543	36971	72	2	CL1Contig4201	10	Dehydroascorbate reductase	Q0ZNW5_MALDO
544	13315	71	2	FQEH0BT01AH1YB	1	Uncharacterized protein	A9PGQ6_POPTR
545	56865	71	1	CL1Contig7705	17	H/ACA ribonucleoprotein complex subunit 4	DKC1_ARATH
546	17370	71	2	FQEH0BT01DOFAF	1	Adenosylhomocysteinase	Q84RD8_MEDTR
547	19775	71	1	CL1Contig1370	14	Peroxisome-type ascorbate peroxidase	B3TLT1_ELAGV
548	66908	71	2	CL713Contig1	23	Splicing factor 3 subunit 1	SF3A1_ARATH
549	12061	71	2	FQEH0BT01AYGAB	1	Cytochrome P450	A9ZT62_COPJA
550	11263	71	2	FQEH0BT01CHHD1	1	Ribosomal protein S10	A4PUT2_MEDTR
551	62742	71	2	CL515Contig2	34	Protein transport protein Sec24	Q2R4A0_ORYSJ
552	41066	71	1	CL639Contig1	9	Tripeptidyl peptidase II	Q6ESI7_ORYSJ
553	22550	71	1	CL7492Contig1	2	Mannose 6-phosphate reductase	Q9FVN7_ORORA
554	48115	71	1	CL1859Contig2	9	Adapter-related protein complex 4 epsilon 1 subunit	Q8RZX0_ORYSJ
555	66072	71	2	CL705Contig2	30	Uncharacterized protein	Q56XB4_ARATH
556	26427	71	1	CL1Contig10471	5	Predicted protein	A9RHR6_PHYPA
557	29555	71	3	CL133Contig1	18	Pyruvate kinase	Q2QXR8_ORYSJ
558	15396	71	2	FQEH0BT01DOI88	1	Uncharacterized protein	A9SVW7_PHYPA
559	58230	71	1	CL162Contig3	50	Growth regulator	Q6ZFX8_ORYSJ
560	42809	71	3	CL1Contig10386	85	Uncharacterized protein	B7FH64_MEDTR
561	23289	71	2	CL5164Contig1	7	Ubiquitin specific protease 12	B6V765_TOBAC
562	22622	70	1	CL141Contig2	2	Coatomer beta subunit 1	COPB1_ARATH
563	12699	70	3	FQEH0BT01ET2J6	1	Protein disulfide-isomerase	PDI_DATGL
564	27571	70	2	CL1Contig6658	4	Glutathione *S*-transferase	Q5GMM5_CAPCH
565	40171	70	2	CL844Contig2	10	Arginyl-tRNA synthetase	O23247_ARATH
566	47789	70	2	CL58Contig3	59	Uncharacterized protein	B7FGT0_MEDTR
567	19399	70	1	CL1Contig10397	4	Uncharacterized protein	B7FJH0_MEDTR
568	37035	70	3	CL2781Contig2	8	RAB7D	Q40214_LOTJA
569	11623	70	2	FQEH0BT01C4OL5	1	Phosphoglucomutase	Q8L893_9ROSI
570	14788	70	2	FQEH0BT01CCPT4	1	Actin-7	B6SZ83_MAIZE
571	12510	70	1	FQEH0BT01BH0OW	1	26S proteasome regulatory subunit 3	PSMD3_TOBAC
572	40278	70	2	CL1Contig2685	32	Uncharacterized protein	Q852A3_ORYSJ
573	82637	70	1	CL1953Contig1	20	Adapitin protein	Q6JJ39_IPOTF
574	14329	70	3	CL1Contig10403	3	Uncharacterized protein	A9P9L2_POPTR
575	28123	70	1	CL4851Contig1	6	Acetyl co-enzyme A carboxylase subunit	B5LAS7_CAPAN
576	20457	70	1	CL3881Contig2	3	Uncharacterized protein	A9TM50_PHYPA

577	44201	70	1	CL1Contig2244	38	Nectarin IV	Q3KU27_NICLS
578	13058	70	2	FQEH0BT01DPLUT	1	Enoyl-acyl carrier protein reductase	Q93X63_BRANA
579	60016	70	3	CL132Contig1	31	Chromosome maintenance protein 4	SMC4_ARATH
580	108440	70	1	CL188Contig3	59	ATP-dependent Clp protease subunit	CLPAB_SOLLC
581	15452	70	1	CL1Contig8836	5	No Hit	
582	33202	70	2	CL1Contig6128	38	Calcium-binding allergen Ole e 8	ALL8_OLEEU
583	20703	70	1	CL1Contig7290	41	40S ribosomal protein S5	Q6RH11_CAPAN
584	23553	70	1	CL1Contig6010	7	Uncharacterized protein	Q9ASV5_ARATH
585	15438	70	1	CL590Contig4	3	*N*-acetyl-gamma-glutamyl-phosphate reductase	A9NUH1_PICSI
586	12558	69	2	FQEH0BT01CC05N	1	Uncharacterized protein	B7FM80_MEDTR
587	12305	69	1	FQEH0BT01B1ROK	1	Uncharacterized protein	A9P7W5_POPTR
588	18830	69	5	FQEH0BT01CEL8G	1	40S ribosomal protein S9	Q60CZ2_SOLDE
589	10774	69	1	FQEH0BT01A1RPF	1	Prohibitin 1-like protein	Q9AXM0_BRANA
590	23765	69	2	CL1Contig2899	7	Superoxide dismutase	A4ZYP8_PENAM
591	84173	69	1	CL1Contig1878	26	Uncharacterized protein	Q0WL65_ARATH
592	25350	69	3	CL305Contig1	27	Phosphoglucomutase	PGMC_PEA
593	67555	69	1	CL259Contig1	85	Uncharacterized protein	B7FIU4_MEDTR
594	76035	69	2	CL124Contig2	24	Clathrin heavy chain	Q2RBN7_ORYSJ
595	83448	69	1	CL960Contig2	24	Protein prenyltransferase	Q2HVW5_MEDTR
596	22747	69	1	CL3040Contig2	11	Uncharacterized protein	Q84QT0_ORYSJ
597	43522	68	1	CL1539Contig1	24	Inorganic pyrophosphatase	Q2P9V0_9MAGN
598	18104	68	2	FQEH0BT01AFS4A	1	Heat shock protein 101 kDa	Q9ZT13_TOBAC
599	17086	68	1	FQEH0BT01DL9C2	1	60S ribosomal protein L13	Q6TKT4_SOLBR
600	12991	68	1	FQEH0BT01EZ3LF	1	Aspartokinase	A9NUI1_PICSI
601	101765	68	3	CL886Contig3	34	Insulin degrading enzyme	Q93YG9_SOLLC
602	48603	68	2	CL258Contig4	13	Ubiquitin specific protease 12	B6V765_TOBAC
603	21322	68	1	CL3681Contig1	6	Initiation factor 2 subunit family protein	Q2R8T8_ORYSJ
604	19622	68	1	CL287Contig2	5	Annexin 1	Q69DC2_GOSHI
605	48745	68	1	CL1234Contig1	14	Sucrose-phosphate synthase 1	SPS1_CITUN
606	19625	68	1	CL4183Contig2	2	Aluminum-induced protein	B3TM05_ELAGV
607	19934	68	1	CL1Contig8351	5	Nuclear cap-binding protein CBP80	Q9AWB3_ORYSJ
608	49430	68	2	CL1308Contig1	15	Hsp70-interating protein 1	B1PDS1_9MAGN
609	75408	68	1	CL1Contig1073	40	Dihydroflavonol reductase	Q9SGE0_ARATH
610	42571	67	2	CL1Contig7560	20	Enoyl-acyl carrier protein reductase	Q93X66_BRANA
611	22110	67	1	CL1Contig7735	2	Uncharacterized protein	A3AA09_ORYSJ
612	28127	67	1	CL1Contig6225	22	Inosine-5'-monophosphate dehydrogenase	Q8GXQ8_ARATH
613	24209	67	1	CL1Contig6221	18	Uncharacterized protein	A9TXE7_PHYPA
614	17152	67	1	CL1Contig1281	2	Ubiquitin-activating enzyme E1	Q8RX82_ARATH
615	73673	67	3	CL47Contig4	183	Isocitrate dehydrogenase	A0EJ87_9ROSI
616	12767	67	2	FQEH0BT01C8GSW	1	Sal m 1 allergen	A8CVH3_SALMI
617	17928	66	1	FQEH0BT01C686D	1	Uncharacterized protein	A9U2F5_PHYPA
618	33026	66	1	CL1Contig7818	69	GF14 protein	Q49082_FRIAG
619	69900	66	1	CL1Contig1130	187	60S ribosomal protein L4	RL4_PRUAR
620	16250	66	1	FQEH0BT01EV6CY	1	Monodehydroascorbate reductase	Q0WUJ1_ARATH
621	28736	66	1	CL5663Contig1	7	HUELLENLOS-like protein	Q676X9_HYAOR
622	41433	66	1	CL2670Contig1	9	Ribose-phosphate pyrophosphokinase 4	KPRS4_SPIOL
623	48018	66	1	CL1Contig7587	11	Methionyl-tRNA synthetase	SYM_ARATH
624	29565	66	3	CL1440Contig1	16	Small nuclear ribonucleoprotein E	B6SK95_MAIZE
625	20015	66	2	CL1Contig1074	23	Calmodulin	Q9ZTV2_PHAVU
626	21110	66	2	CL1Contig5036	3	Valyl-tRNA synthetase	SYV_ARATH
627	88921	66	1	CL1588Contig1	25	Ubiquitin carboxyl-terminal hydrolase 14	UBP14_ARATH
628	57511	65	2	CL569Contig4	23	Uncharacterized protein	A9PFZ9_POPTR
629	12663	65	2	FQEH0BT01A0PFA	1	Splicing factor SR1C	Q9SPI5_ARATH
630	31521	65	1	CL1Contig7759	7	Small nuclear ribonucleoprotein D2	Q8RUH0_ARATH
631	12202	65	6	FQEH0BT01AE6R8	1	Uncharacterized protein	Q9ARZ9_ORYSJ
632	29686	65	1	CL499Contig1	44	Uncharacterized protein	A9NPK8_PICSI
633	115783	65	2	CL1Contig3294	863	Methionine synthase	A6YGE7_CARPA
634	19240	65	1	CL601Contig3	6	Uncharacterized protein	A9PF72_POPTR

635	12383	65	4	FQEH0BT01D6WBW	1	Carboxypeptidase type III	Q8L6A7_THECC
636	18571	65	1	CL1805Contig2	4	Uncharacterized protein	A9PAA7_POPTR
637	14952	65	2	FQEH0BT01DDPW6	1	Glutathione *S*-transferase 18	Q9FQE0_SOYBN
638	14237	65	2	FQEH0BT01EQ2LD	1	Elongation factor 1-alpha	Q9LN13_ARATH
639	63389	65	2	CL825Contig2	22	Acetyltransferase-like protein	Q5ZDJ3_ORYSJ
640	15436	65	1	FQEH0BT01EMIBC	1	Benzoquinone reductase	A3F7Q3_GOSHI
641	48591	64	1	CL1Contig4253	26	ATP-dependent Clp protease proteolytic subunit	A9PA38_POPTR
642	63876	64	2	CL531Contig1	27	Inositol polyphosphate kinase	A4H2J0_SOLTU
643	16876	64	1	FQEH0BT01EV4UI	1	Glutathione *S*-transferase	Q93WY5_ORYSJ
644	12988	64	1	CL1003Contig2	4	Uncharacterized protein	Q0WMN4_ARATH
645	26863	64	1	CL16918Contig1	2	Uncharacterized protein	A2X7Z3_ORYSI
646	25485	64	1	CL67Contig2	2	6-phosphogluconate dehydrogenase	Q9FWA3_ARATH
647	40215	64	1	CL2271Contig1	18	Uncharacterized protein	A9P9B6_POPTR
648	61765	64	1	CL469Contig3	36	Uncharacterized protein	A2Z6H7_ORYSI
649	16250	64	1	FQEH0BT01BJH38	1	Plasma membrane proton ATPase	Q9M461_PRUPE
650	19441	64	2	CL260Contig2	3	Uncharacterized protein	A9P9G9_POPTR
651	60092	64	1	CL100Contig1	146	Transaldolase	Q9LYR4_ARATH
652	63115	64	1	CL1413Contig1	25	Translation initiation factor IF-2	Q9SRD2_ARATH
653	50289	64	1	CL1Contig1062	31	3-Isopropylmalate dehydrogenase small subunit	B5LAV2_CAPAN
654	19417	64	2	CL449Contig2	2	Dihydrolipoyl dehydrogenase, mitochondrial	DLDH_PEA
655	54934	63	2	CL1Contig6596	42	60S ribosomal protein L7a	B4FPB7_MAIZE
656	120804	63	1	CL1Contig8524	156	Poly(A)-binding protein	Q9AT32_DAUCA
657	14473	63	1	CL7Contig4	2	Gamma-kafirin preprotein	Q41506_SORBI
658	22979	63	1	CL230Contig4	23	Uncharacterized protein	B7FLL3_MEDTR
659	39123	63	1	CL186Contig10	8	26S proteasome subunit RPN2a	B1Q487_CAPCH
660	45605	63	2	CL2038Contig1	20	Uncharacterized protein	A9PEC2_POPTR
661	21711	63	1	CL6594Contig1	5	Uncharacterized protein	B7FK39_MEDTR
662	14805	63	1	FQEH0BT01DQ0IH	1	Rubisco subunit binding-protein alpha subunit	Q2PEW7_TRIPR
663	59011	63	1	CL1Contig2249	91	Dolichyl-di-phosphooligosaccharide glycotransferase	Q944K2_ARATH
664	49549	63	3	CL337Contig3	14	Cytosolic factor	Q2PF01_TRIPR
665	21225	63	1	CL261Contig3	17	Cysteine protease inhibitor cystatin	Q84LB7_MALDO
666	16156	62	1	FQEH0BT01CD06T	1	Glucose-6-phosphate isomerase	G6PI_ARATH
667	59621	62	1	CL293Contig2	47	Uncharacterized protein	A9P841_POPTR
668	13457	62	1	FQEH0BT01CVCO7	1	Translational activator	Q6ASU8_ORYSJ
669	41445	62	2	CL1Contig6210	12	Eukaryotic translation initiation factor 3 subunit A	EIF3A_TOBAC
670	33128	62	1	CL1225Contig4	7	Uncharacterized protein	Q9SX53_ARATH
671	58350	62	1	CL26Contig4	68	Oxidoreductase 2OG-Fe(II) oxygenase	Q9LE86_ARATH
672	38118	62	1	CL1094Contig3	15	Uncharacterized protein	B4F9M6_MAIZE
673	28282	62	1	CL1Contig7003	3	Polyphenol oxidase	PPO_MALDO
674	15475	62	2	FQEH0BT01BFLG1	1	Adenosylhomocysteinase	Q5D6C5_ARATH
675	85654	62	1	CL1Contig4756	320	Citrate synthase	Q6T8H1_9ROSI
676	25748	62	1	CL1Contig5931	6	Calmodulin	CALM_MALDO
677	14433	62	1	FQEH0BT01EVS7I	1	Hexokinase 1	B6V3C0_CUCME
678	11371	62	1	FQEH0BT01DZUJM	1	Early-responsive-to-dehydration protein	B0FSL2_MAIZE
679	56484	62	1	CL590Contig3	44	*N*-acetyl-gamma-glutamyl-phosphate reductase	A9NUH1_PICSI
680	21757	62	1	CL630Contig3	3	Uncharacterized protein	A9PI99_POPTR
681	18873	62	2	FQEH0BT01CA2X7	1	Glutathione *S*-transferase	Q8H9E5_CUCMA
682	54328	62	2	CL1Contig4008	37	Ubiquitin-activating enzyme E1	P93393_TOBAC
683	34372	62	2	CL283Contig3	42	Proteasome alpha subunit type 6	PSA6_SOYBN
684	24264	62	1	CL6795Contig1	4	Uncharacterized protein	Q9LMB3_ARATH
685	48975	62	2	CL1Contig5139	31	Annexin	B3TLL7_ELAGV
686	21644	62	2	CL1Contig5489	16	Uncharacterized protein	A9NNV8_PICSI
687	38426	61	1	CL2987Contig1	10	Inorganic pyrophosphatase	Q2P9V1_9MAGN
688	25747	61	1	CL1Contig4711	37	Fructose-bisphosphate aldolase	Q9SJQ9_ARATH
689	46487	61	2	CL287Contig1	32	Annexin	P93157_GOSHI
690	15163	61	1	CL179Contig1	2	Uncharacterized protein	A9PDH3_POPTR
691	8099	61	1	FQEH0BT01BKNVL	1	No Hit	
692	49838	61	1	CL241Contig2	38	SGT1-2	A5H2U4_SOLLC

693	13444	61	1	FQEH0BT01BHYSN	1	ATP synthase subunit E, vacuolar	B3TLU2_ELAGV
694	28220	61	1	CL1Contig5914	4	Initiation factor eIF4 gamma	Q2HUL1_MEDTR
695	27561	61	1	CL90Contig7	20	ATP synthase subunit O	ATPO_IPOBA
696	9170	61	1	CL19167Contig1	2	Villin	Q541Y5_ARATH
697	75367	61	1	CL70Contig1	170	Uncharacterized protein	A9PD77_POPTR
698	37498	61	2	CL1Contig3433	30	Glycoprotein-like protein	Q3HVK7_SOLTU
699	74536	61	2	CL1Contig10062	30	Uncharacterized protein	A9PEK1_POPTR
700	38820	61	2	CL2513Contig1	16	GTP-binding protein SAR1A	SAR1A_ARATH
701	45332	61	1	CL127Contig1	40	Alpha-soluble NSF attachment protein	B3TM20_ELAGV
702	40993	61	2	CL197Contig1	19	Phospholipase D2	Q8VWE9_PAPSO
703	85799	61	1	CL1Contig5213	41	Oligopeptidase A	Q9LSL3_ARATH
704	15978	61	1	FQEH0BT01DM7UP	1	Glycine-rich RNA-binding protein GRP1A	Q2QLR2_ORYSJ
705	19353	61	1	FQEH0BT01BJE6W	1	Cellulose synthase-like protein E1	CSLE1_ORYSJ
706	54295	61	1	CL1988Contig2	18	Enolase	A9P745_HELAN
707	13028	61	1	FQEH0BT01EVN7O	1	Uncharacterized protein	A9P851_POPTR
708	40003	61	2	CL1291Contig5	6	Exportin-2	XPO2_ARATH
709	39543	61	1	CL417Contig1	47	Proteasome beta subunit type 5	PSB5_SPIOL
710	61373	61	1	CL251Contig1	82	Uncharacterized protein	B7FKW8_MEDTR
711	38243	61	1	CL4610Contig2	6	Translational activator	Q6ASU8_ORYSJ
712	29001	60	1	CL1Contig4755	38	Uncharacterized protein	A9PA31_POPTR
713	22773	60	2	CL121Contig1	10	Glutamine synthetase	Q42625_BRANA
714	47682	60	4	CL1Contig3486	22	Uncharacterized protein	A9PE52_POPTR
715	79535	60	3	CL186Contig4	49	Initiation factor eIF4 gamma	Q6XJG7_ARATH
716	37257	60	1	CL20Contig11	20	3-Phosphoshikimate 1-carboxyvinyltransferase	Q1M157_CAMAC
717	7745	60	2	FQEH0BT01DVVVO	1	Uncharacterized protein	A9P801_POPTR
718	95586	60	1	CL1208Contig1	31	Uncharacterized protein	A2Y0G8_ORYSI
719	24326	60	1	CL1Contig8461	5	(*S*)-*N*-methylcoclaurine 3'-hydroxylase	O64901_ESCCA
720	14504	60	1	FQEH0BT01DAERG	1	Phosphoglucomutase	Q9AUQ4_ORYSA
721	8310	60	2	FQEH0BT01C45J2	1	Calreticulin	CALR_BERST
722	12560	60	2	FQEH0BT01D7N13	1	Nectarin IV	Q3KU27_NICLS
723	8532	60	2	FQEH0BT01DCU1G	1	Translation elongation factor beta chain	B3TLP3_ELAGV
724	29016	60	2	CL912Contig3	4	Uncharacterized protein	Q9M3Z2_CICAR
725	16390	60	1	FQEH0BT01B4BZD	1	L3 Ribosomal protein	Q9SBR8_MEDVA
726	18418	60	3	CL1Contig7999	4	Uncharacterized protein	Q8RWJ0_ARATH
727	72349	60	2	CL587Contig5	18	Uncharacterized protein	A9PF13_POPTR
728	21538	60	2	CL9542Contig1	4	Chaperonin CPN60-like 2, mitochondrial	CH60C_ARATH
729	36275	60	1	CL3033Contig1	10	Glutamate/malate translocator	Q8L7Z8_TOBAC
730	12647	60	1	FQEH0BT01A85VW	1	S28 ribosomal protein	Q7X9K4_WHEAT
731	18084	60	1	FQEH0BT01DTE5C	1	Guanine nucleotide-exchange protein GEP2	Q7XIK7_ORYSJ
732	18755	60	1	FQEH0BT01A830J	1	Uncharacterized protein	A9PJ22_POPJC
733	37998	60	2	CL3654Contig1	11	Dihydrodipicolinate reductase 2	DAPB2_ARATH
734	83182	60	1	CL890Contig3	30	AKIN beta gamma	Q944A6_ARATH
735	30035	60	1	CL1Contig6700	8	Triosephosphate isomerase, chloroplastic	TPIC_ARATH
736	68947	60	2	CL639Contig3	16	Uncharacterized protein	Q8L640_ARATH
737	16527	60	1	FQEH0BT01CTT6I	1	Translational activator	Q6ASU8_ORYSJ
738	22098	60	1	CL3739Contig2	2	L-Galactose-1-phosphate phosphatase	Q5U789_ACTDE
739	14014	59	2	CL1Contig1211	2	Calreticulin	CALR_BETVU
740	48895	59	2	CL16Contig10	29	Endo-1,3-1,4-beta-D-glucanase	Q9LUG8_ARATH
741	55140	59	1	CL1Contig159	184	Malate dehydrogenase	Q9FSF0_TOBAC
742	15772	59	1	FQEH0BT01E3JQX	1	Uncharacterized protein	A9PCC9_POPTR
743	30648	59	1	CL2150Contig2	14	Pathogenesis-related 10 protein PR10-1	Q4QTJ0_PAPSO
744	13397	59	2	FQEH0BT01EN4DS	1	Uncharacterized protein	A9PIQ2_POPJC
745	44253	59	1	CL582Contig2	47	Uncharacterized protein	A9PHG3_POPTR
746	15841	59	1	CL1534Contig3	2	Uncharacterized protein	B4FR08_MAIZE
747	11100	59	1	FQEH0BT01ADD61	1	Uncharacterized protein	A3BPB0_ORYSJ
748	83519	59	2	CL1Contig7659	111	Ketol-acid reductoisomerase	B5LAT1_CAPAN
749	26075	59	1	CL1Contig9749	42	QM-like protein	B3TLM0_ELAGV
750	114233	59	3	CL1Contig9234	52	Uncharacterized protein	B3H5Y0_ARATH

751	15124	59	1	FQEH0BT01AJNWM	1	Spondyloepiphyseal dysplasia	Q6K5Z3_ORYSJ
752	11401	59	1	FQEH0BT01AJ6JW	1	*N*-acetyltransferase	B4UWD7_ARAHY
753	62188	59	2	CL318Contig3	33	Aldehyde oxidase 1	Q2PHF4_LACSA
754	17054	59	1	CL666Contig2	2	Adenylosuccinate synthetase, chloroplastic	PURA_WHEAT
755	35585	59	2	CL8996Contig1	3	Heat shock protein	Q4LDR0_SOLLC
756	105945	59	2	CL540Contig2	38	Eukaryotic initiation factor subunit	Q8S7Q0_ORYSJ
757	25329	59	1	CL14710Contig1	2	Uncharacterized protein	Q84QA4_ORYSJ
758	37843	59	1	CL470Contig2	7	Alanine aminotransferase	Q5GAU1_MAIZE
759	15874	59	1	FQEH0BT01EE7J9	1	Putative uncharacterized protein	Q852A3_ORYSJ
760	16753	58	1	FQEH0BT01ECEA0	1	Putative splicing factor Prp8	Q45NM0_MEDSA
761	15252	58	2	FQEH0BT01BJ8MB	1	Dirigent-like protein pDIR1	Q27JB1_PICGL
762	26204	58	2	CL1Contig8787	3	Berberine bridge enzyme	RETO_PAPSO
763	95555	58	1	CL138Contig4	105	Polyphenol oxidase	PPO_VITVI
764	14743	58	2	FQEH0BT01DMWYY	1	Aspartic proteinase	Q401N7_WHEAT
765	11235	58	2	FQEH0BT01DRCVG	1	60S ribosomal protein L26-2	RL262_ARATH
766	15645	58	1	FQEH0BT01CEMDF	1	Uncharacterized protein	A9NVX1_PICSI
767	64402	58	2	CL26Contig3	31	Oxidoreductase 2OG-Fe(II) oxygenase	Q9LE86_ARATH
768	14166	58	1	FQEH0BT01CCCHH	1	Multiprotein bridging factor 1	Q9LL86_SOLTU
769	10375	58	1	CL165Contig9	2	VDAC2.1	Q6W2J2_LOTJA
770	63520	58	2	CL1Contig9409	112	GDP-mannose 3,5-epimerase 1	B6T588_MAIZE
771	17579	57	1	CL1344Contig3	7	Small molecular heat shock protein 10	A3FPF3_NELNU
772	32752	57	1	CL1Contig653	5	60S ribosomal protein L27A	B6VC54_VERFO
773	20600	57	1	FQEH0BT01BQLZS	1	Uncharacterized protein	B0BLB2_LOTJA
774	73816	57	1	CL1Contig2583	51	Ubiquitin family protein, expressed	Q10S80_ORYSJ
775	82618	57	1	CL703Contig1	34	Protein transport protein Sec24-like	SC24A_ARATH
776	29555	57	1	CL1143Contig1	32	Peptidyl-prolyl cis-trans isomerase	A9NLK9_PICSI
777	14798	57	1	CL825Contig4	2	N-terminal acetyltransferase	Q9M8L0_ARATH
778	14108	57	1	CL1831Contig1	2	Argininosuccinate synthase, chloroplastic	ASSY_ARATH
779	11109	57	1	FQEH0BT01BNKHD	1	Uncharacterized protein	Q6AV23_ORYSJ
780	20761	57	1	CL1Contig4029	29	Phosphoglycerate kinase	PGKY_TOBAC
781	17509	57	2	CL1Contig9487	3	Fructose-bisphosphate aldolase	Q38HV4_SOLTU
782	17186	57	1	FQEH0BT01EL4E5	1	Cytoplasmic ribosomal protein S13	Q9MAV9_PANGI
783	45783	57	1	CL1831Contig2	19	Argininosuccinate synthase, chloroplastic	ASSY_ARATH
784	38710	57	2	CL238Contig1	27	Uncharacterized protein	A9PEC2_POPTR
785	12648	57	2	FQEH0BT01B6LSU	1	GTP-binding protein	Q9SA73_ARATH
786	42295	56	1	CL3219Contig1	13	Uncharacterized protein	Q60E58_ORYSJ
787	12949	56	1	CL14406Contig1	2	Chloroplast chaperonin	Q8S7D5_ORYSA
788	41243	56	1	CL208Contig3	11	UDP-glucose:glycoprotein glucosyltransferase	Q9FVU8_ARATH
789	47004	56	1	CL3193Contig1	13	Uncharacterized protein	A9U407_PHYPA
790	13733	56	1	FQEH0BT01CITBA	1	Uncharacterized protein	A9PGL4_POPTR
791	19052	56	1	CL1Contig382	4	Uncharacterized protein	A9PD55_POPTR
792	16582	56	1	FQEH0BT01CTG9J	1	Uncharacterized protein	Q9S9T5_ARATH
793	49074	56	1	CL1665Contig1	12	Uncharacterized protein	B7FKA1_MEDTR
794	26545	56	1	CL4699Contig1	5	Uncharacterized protein	A9PAU1_POPTR
795	16849	56	1	CL15226Contig1	2	NOGO-interacting protein, mitochondrial	Q5JMM6_ORYSJ
796	31968	56	1	CL2218Contig2	11	Signal peptidase complex subunit 2	SPCS2_ARATH
797	81866	56	1	CL1318Contig1	29	Cytochrome P450	A9ZT59_COPJA
798	17082	56	1	CL1105Contig2	7	Transmembrane protein	Q7XI54_ORYSJ
799	54277	56	1	CL477Contig4	16	Uncharacterized protein	A3AB81_ORYSJ
800	15855	56	1	FQEH0BT01ESIOA	1	Calreticulin	CALR_BERST
801	17277	56	1	FQEH0BT01A4DCI	1	MtN19	Q6ZD31_ORYSJ
802	25615	56	1	CL3487Contig1	12	Nuclear transport factor 2	Q8LC75_ARATH
803	96072	56	2	CL115Contig2	33	Coatomer alpha subunit	Q70I39_LOTJA
804	14897	56	1	CL1Contig9568	3	Uncharacterized protein	A9PAK9_POPTR
805	38450	56	1	CL1Contig2501	65	Uncharacterized protein	B7FMK4_MEDTR
806	63329	55	1	CL1Contig9300	52	Uncharacterized protein	A9SV98_PHYPA
807	24472	55	1	CL1Contig10332	2	Non-symbiotic hemoglobin 2	HBL2_BRANA
808	36948	55	1	CL1Contig5408	17	Splicing factor	B6TDA1_MAIZE

809	69460	55	1	CL460Contig2	21	Uncharacterized protein	Q9SAJ2_ARATH
810	17340	55	1	FQEH0BT01A2PX4	1	1-acyl-sn-glycerol-3-phosphate acyltransferase	B2LWM3_RICCO
811	6694	55	1	FQEH0BT01D4621	1	Adenosylhomocysteinase	Q2XTD0_SOLTU
812	44564	55	1	CL511Contig2	17	T-complex protein 1 theta chain	Q653F6_ORYSJ
813	26118	55	1	CL9599Contig1	4	Uncharacterized protein	B7FGI3_MEDTR
814	12254	55	1	FQEH0BT01BDH9L	1	Reversibly glycosylated polypeptide	Q8VWN8_GOSHI
815	72940	55	1	CL75Contig3	23	Uncharacterized protein	Q9SA62_ARATH
816	68475	55	1	CL225Contig1	94	3-Ketoacyl-CoA thiolase	P93112_9ROSI
817	28806	55	1	CL806Contig3	6	Transaldolase	Q7XAS4_GOSHI
818	69920	55	1	CL307Contig1	38	Uncharacterized protein	Q940J9_ARATH
819	20107	55	1	CL1Contig6481	2	Subtilsin-like protease	A9XG41_TOBAC
820	12228	55	1	FQEH0BT01EWPT7	1	Cytochrome c	CYC_SAMNI
821	21856	55	1	CL64Contig2	7	Fumarate hydratase 1, mitochondrial	FUM1_ARATH
822	20903	55	1	CL1Contig9770	12	EBP1	A0FH76_SOLTU
823	38880	55	1	CL4872Contig1	8	Cysteinyl-tRNA synthetase	O82267_ARATH
824	38396	55	1	CL1Contig9211	14	Ubiquitin-activating enzyme E1	Q75VJ8_TOBAC
825	31105	55	1	CL1Contig7365	27	Uncharacterized protein	A9NK75_PICSI
826	81575	54	1	CL222Contig2	53	Glucosidase II alpha subunit	Q9FN05_ARATH
827	59196	54	1	CL153Contig3	111	Disulfide-isomerase-like protein	B3TLY7_ELAGV
828	25468	54	1	CL594Contig4	27	CHP-rich zinc finger protein	Q84S20_ORYSJ
829	68155	54	2	CL496Contig2	35	Glutathione reductase	Q0GA75_RHEAU
830	17540	54	1	FQEH0BT01EIVW2	1	NADH dehydrogenase subunit 7	Q9MF86_BETVU
831	20959	54	2	CL12231Contig1	3	Glycoprotein	Q3HVK7_SOLTU
832	58187	54	1	CL29Contig4	32	3,4-Dihyroxy-2-butanone kinase	DHBK_SOLLC
833	34322	54	1	CL1Contig9896	14	Eukaryotic translation initiation factor 3 subunit A	EIF3A_TOBAC
834	24376	54	1	CL10050Contig1	3	Embryo-specific protein 3	Q9LVB6_ARATH
835	13942	54	1	FQEH0BT01BGM4R	1	Uncharacterized protein	B6SHC8_MAIZE
836	15827	54	2	CL1Contig1563	2	60S ribosomal protein L27	Q9MAV8_PANGI
837	39968	54	1	CL140Contig5	31	Dehydrogenase complex E1 alpha subunit	A8IXJ9_BRACM
838	32808	54	1	CL1Contig9306	70	Ribosomal protein S13	Q2V995_SOLTU
839	67401	54	1	CL896Contig2	31	Peptide chain release factor subunit 1 protein	Q75I90_ORYSJ
840	18574	54	1	CL1147Contig1	31	Proteasome beta subunit	Q0J0L6_ORYSJ
841	88384	54	1	CL550Contig2	44	ATP-binding cassette protein GCN3	GCN3_ARATH
842	20456	54	2	CL203Contig1	7	Transketolase 1	O78327_CAPAN
843	17737	54	1	FQEH0BT01BA49A	1	Eukaryotic translation initiation factor 3 (EIF-3)	Q1EPJ8_MUSAC
844	69433	54	1	CL1Contig9422	61	Aminotransferase	Q6VMN8_ORYSJ
845	41707	54	1	CL2097Contig1	19	Uncharacterized protein	A9PD04_POPTR
846	19724	54	1	CL1Contig10559	3	Tetrahydroprotoberberine-*N*-methyltransferase	Q108P1_PAPSO
847	12693	54	1	FQEH0BT01ARCXO	1	Uncharacterized protein	A3AP61_ORYSJ
848	26329	53	1	CL1Contig7380	24	Methionyl-tRNA synthetase	SYM_ORYSJ
849	49674	53	1	CL819Contig1	35	Uncharacterized protein	A9PCQ2_POPTR
850	34640	53	1	CL6769Contig1	5	U2 snRNP protein A'	Q6EUK2_ORYSJ
851	16842	53	1	FQEH0BT01D2AAK	1	Spermidine synthase	A2VC28_LOTJA
852	17699	53	1	CL360Contig2	7	Uncharacterized protein	A9PBF5_POPTR
853	28969	53	1	CL1Contig9954	3	Norcoclaurine 6-*O*-methyltransferase	Q7XB09_PAPSO
854	47582	53	1	CL839Contig2	8	Glutathione *S*-transferase	Q8H9E5_CUCMA
855	51091	53	1	CL1Contig3432	32	Uncharacterized protein	A0B9Y0_ORYSA
856	81718	53	2	CL203Contig2	50	Uncharacterized protein	A9P7Z7_POPTR
857	16046	53	1	CL1Contig5643	3	GTPase	Q307Y7_SOLTU
858	61962	53	1	CL1Contig4229	38	Uncharacterized protein	Q8VZ33_ARATH
859	32708	53	1	CL6101Contig1	5	Nuclear pore complex protein Nup155	Q5Z4E0_ORYSJ
860	60436	53	1	CL2885Contig1	14	Uncharacterized protein	Q9LHN3_ARATH
861	14965	53	1	CL584Contig1	2	UTP--glucose-1-phosphate uridylyltransferase	UGPA_ASTME
862	40659	53	1	CL155Contig2	70	Fructokinase 2	Q7XAE3_PETIN
863	16866	53	1	CL1Contig3731	3	Serine hydroxymethyltransferase	A9PL11_POPTM
864	45477	53	2	CL114Contig2	13	Uncharacterized protein	Q541W5_ARATH
865	36937	53	1	CL1Contig2695	43	Cullin 1-like protein C	A0ELU7_PETIN
866	25517	53	1	CL1126Contig1	10	Uncharacterized protein	A9PIB2_POPTR

867	68214	53	1	CL1Contig6890	22	Uncharacterized protein	A3C7K8_ORYSJ
868	117963	53	2	CL263Contig3	37	G2484-1 protein	O23575_ARATH
869	19321	53	1	CL2633Contig2	8	Mago nashi-like protein	Q5YJQ9_HYAOR
870	33367	52	2	CL1058Contig2	6	Ttranslational activator	Q9XIR5_ARATH
871	61241	52	1	CL1002Contig2	21	Glycine dehydrogenase, mitochondrial	GCSP_SOLTU
872	17170	52	1	CL7477Contig1	4	Separation anxiety protein	Q9LFM3_ARATH
873	19301	52	1	CL1Contig2367	13	Poly(A)-binding protein	Q9M6E4_TOBAC
874	24402	52	1	CL1320Contig5	5	Selenium-binding protein	Q93WS1_MEDSA
875	23253	52	1	CL7263Contig1	5	Uncharacterized protein	Q6YZH8_ORYSJ
876	15892	52	1	FQEH0BT01E0PUB	1	Norcoclaurine synthase 1	Q4QTJ2_PAPSO
877	30746	52	1	CL485Contig3	37	RPN9	O076B1_NICBE
878	14192	52	1	FQEH0BT01BD56L	1	Uncharacterized protein	A9PC60_POPTR
879	21927	52	1	CL7390Contig1	5	Uncharacterized protein	A9PCA8_POPTR
880	42460	52	2	CL1025Contig1	11	Uncharacterized protein	A9P7V1_POPTR
881	51411	52	1	CL727Contig1	43	Ubiquitin activating enzyme	Q9LUF3_ARATH
882	15694	52	1	FQEH0BT01DAS8X	1	Uncharacterized protein	B7FGX4_MEDTR
883	34581	52	1	CL1Contig6040	8	Dolichyl-di-phosphooligosaccharide protein	Q6ZLK0_ORYSJ
884	61869	52	1	CL1Contig5073	146	Phosphate transporter, mitochondrial	A8TU59_PAESU
885	30096	52	3	CL37Contig6	36	Uncharacterized protein	A9PE02_POPTR
886	33710	52	1	CL422Contig4	12	Uncharacterized protein	A2X9V8_ORYSI
887	10210	52	1	FQEH0BT01DL9JF	1	Uncharacterized protein	A9PGV2_POPTR
888	88661	52	1	CL18Contig13	141	ATP/ADP transport protein 2, chloroplastic	Q1AK42_MANES
889	204995	52	1	CL29Contig6	71	Uncharacterized protein	Q9SSD2_ARATH
890	28131	52	1	CL1492Contig2	5	Uncharacterized protein	Q93ZV7_ARATH
891	29597	52	1	CL3231Contig2	9	Serine carboxypeptidase	Q8VWQ0_GOSHI
892	10256	51	1	FQEH0BT01AKN3A	1	ATP phosphoribosyl transferase, chloroplastic	Q56UT3_ALYLE
893	19679	51	1	CL1Contig9852	3	Norcoclaurine 6-*O*-methyltransferase	Q7XB09_PAPSO
894	31171	51	1	CL1Contig7568	4	CAAX prenyl protease 1 homolog	FACE1_ORYSJ
895	31182	51	1	CL27Contig9	36	Uncharacterized protein	A9PCX2_POPTR
896	52200	51	1	CL951Contig1	32	Aminomethyltransferase, mitochondrial	GCST_SOLTU
897	17990	51	1	FQEH0BT01BR9FW	1	Phenylalanine ammonia-lyase	Q9LEP1_BETVE
898	35408	51	2	CL1291Contig1	15	Exportin-2	XPO2_ARATH
899	35157	51	1	CL6237Contig1	6	Polynucleotide phosphorylase	Q9S7G6_ARATH
900	15488	51	1	FQEH0BT01DSKBM	1	Glutamate synthase	Q9LV03_ARATH
901	14794	51	1	CL47Contig1	2	Isocitrate dehydrogenase	A0EJ87_9ROSI
902	-	51	1	FQEH0BT01AULAX	1	Elongation factor G	Q9SI75_ARATH
903	21102	51	1	CL1Contig3381	6	Uncharacterized protein	A9SRC9_PHYPA
904	16435	51	1	CL4Contig6	2	Uncharacterized protein	Q38HU5_SOLTU
905	24529	51	1	CL232Contig2	8	Elongation factor Tu	Q0WUV8_ARATH
906	9741	51	1	CL17609Contig1	2	Pectinesterase	Q9FY03_9ROSI
907	22953	51	1	CL1Contig546	7	Uncharacterized protein	Y2766_ARATH
908	16642	51	1	FQEH0BT01CJOE9	1	Transaminase/transferase	B6TIG6_MAIZE
909	17493	51	1	CL1Contig8844	2	31 kDa ribonucleoprotein, chloroplastic	ROC4_NICSY
910	17704	51	1	CL1289Contig1	6	14 kDa zinc-binding protein	Q10M57_ORYSJ
911	67263	51	1	CL1376Contig1	16	Nuclear pore protein	Q9FI62_ARATH
912	74230	51	1	CL1Contig8222	46	Uncharacterized protein	A9XLG6_SOLBU
913	30768	50	1	CL2375Contig2	12	Membrane bound *O*-acyltransferase	Q6EP89_ORYSJ
914	43675	50	1	CL1Contig2728	92	Fructose-bisphosphate aldolase	Q38JH2_SOLTU
915	12069	50	1	FQEH0BT01B04C0	1	Uncharacterized protein	B7FLW3_MEDTR
916	15453	50	1	FQEH0BT01AS90K	1	Chaperone GrpE type 2	Q9ZSP3_TOBAC
917	14193	50	1	FQEH0BT01AIFWH	1	Uncharacterized protein	A9PHA6_POPTR
918	36386	50	1	CL1Contig4830	138	Uncharacterized protein	A9PA10_POPTR
919	7603	50	1	FQEH0BT01CNO3J	1	60S ribosomal protein L27	B3TLV4_ELAGV
920	28697	50	3	CL1Contig8445	3	ADP/ATP carrier protein 1	ADT1_GOSHI
921	28079	50	1	CL220Contig2	7	Histone H2A variant 3	B6T3F2_MAIZE
922	48684	50	2	CL1Contig7877	77	Cell division cycle protein	B2M1Y5_9ROSI
923	8419	50	1	FQEH0BT01CJOLP	1	Nuclear-pore anchor	A4GSN8_ARATH
924	17764	50	1	FQEH0BT01EV9ZF	1	Endoplasmin homolog	ENPL_CATRO

925	34036	50	1	CL7812Contig1	5	Histidinol-phosphate aminotransferase, chloroplastic	HIS8_NICPL
926	36837	50	1	CL182Contig4	17	Pyruvate dehydrogenase E1 beta subunit	O24458_ARATH
927	20441	50	1	CL141Contig9	2	Coatomer subunit beta-2	COPB2_ARATH
928	59145	50	1	CL1Contig7095	89	DAHP synthase	Q30D02_FAGSY
929	19965	50	1	CL17Contig3	43	Peptidyl-prolyl cis-trans isomerase	A9PAQ9_POPTR
930	46357	50	2	CL1Contig2921	14	Uncharacterized protein	A9PG97_POPTR
931	24343	50	1	CL5206Contig1	3	Uncharacterized protein	Q9LER5_ARATH
932	12076	50	1	FQEH0BT01DLYKV	1	Thioredoxin H3	Q6VBI4_IPOBA
933	14923	50	1	FQEH0BT01EEJGU	1	Nuclear protein-like	Q9FMF9_ARATH
934	19949	50	1	CL1Contig6258	3	Proteasome subunit alpha type	Q6H852_ORYSJ
935	29981	50	1	CL849Contig2	8	H/ACA ribonucleoprotein complex subunit 1	Q2R242_ORYSJ
936	17295	50	1	FQEH0BT01BYUF4	1	Methionyl-tRNA synthetase	SYM_ARATH
937	111461	49	1	CL1Contig6364	165	Nitrate reductase	NIA_CUCMA
938	29761	49	1	CL861Contig2	13	Vesicle transport v-SNARE 11	Q307Y3_SOLTU
939	12852	49	1	FQEH0BT01A30ZC	1	Uncharacterized protein	B7FGI8_MEDTR
940	20975	49	1	CL4642Contig1	8	Elongation factor protein	Q9LNC5_ARATH
941	10647	49	2	FQEH0BT01AHMP5	1	Uncharacterized protein	B7FFD7_MEDTR
942	15529	49	1	FQEH0BT01DILCZ	1	RNA-binding protein	Q8SMH8_PERAE
943	49952	49	1	CL1Contig6814	36	Coatomer subunit epsilon-1	COPE1_ARATH
944	25903	49	1	CL451Contig2	28	Uncharacterized protein	A2WNG7_ORYSI
945	19497	49	1	CL253Contig4	6	Uncharacterized protein	B6TL97_MAIZE
946	37005	49	1	CL1Contig10131	16	Actin-depolymerizing factor 1	B2CM17_SOLCH
947	70114	49	2	CL1Contig619	150	Enolase	ENO_RICCO
948	17687	49	1	CL3165Contig2	2	Tropinone reductase-like	Q9S9W2_ARATH
949	14389	49	1	FQEH0BT01CYCU2	1	Tryptophan synthase alpha chain	Q06H27_ARAHY
950	66067	49	1	CL2674Contig1	13	Lon protease homolog	Q4ZJ72_ORYSI
951	14918	49	1	FQEH0BT01DK2YZ	1	Aspartate carbamoyltransferase	Q8L6B8_SOLTU
952	25955	49	1	CL16773Contig1	2	Uncharacterized protein	A9S7W1_PHYPA
953	15426	49	2	CL1Contig2914	2	Cell division cycle protein	B2M1Y5_9ROSI
954	55507	49	1	CL2648Contig2	6	PTOR	Q9FR53_ARATH
955	18980	49	1	CL6029Contig2	2	Uncharacterized protein	A3A802_ORYSJ
956	26380	49	1	CL1Contig721	27	Uncharacterized protein	A9TGH1_PHYPA
957	15352	49	1	FQEH0BT01CK6WX	1	UDP-glucoronyltransferase-I	Q653F4_ORYSJ
958	20484	49	1	CL1Contig8831	12	Uncharacterized protein	Q7XLG5_ORYSJ
959	47530	48	1	CL1Contig2592	22	UDP-glucose 4-epimerase	GALE1_PEA
960	34038	48	1	CL1Contig1919	6	Alcohol dehydrogenase	Q19P40_9ROSI
961	98506	48	1	CL510Contig2	32	Uncharacterized protein	Q7XN10_ORYSJ
962	24390	48	1	CL4951Contig3	3	Kinesin, motor region	Q2HTE3_MEDTR
963	16988	48	2	FQEH0BT01BKFU1	1	Uncharacterized protein	Q8LLP7_ORYSJ
964	30133	48	1	CL1Contig3169	5	*S*-adenosylmethionine synthetase	B3GLJ1_PANGI
965	18805	48	1	CL1Contig394	3	Uncharacterized protein	Q0DVV0_ORYSJ
966	17529	48	1	CL1Contig7915	6	Uncharacterized protein	A9P8B1_POPTR
967	19202	48	1	FQEH0BT01BJGTJ	1	Norcoclaurine synthase	B6E2Z2_PAPSO
968	34733	48	1	CL765Contig2	24	Small GTPase Rab2	Q946G3_TOBAC
969	6799	48	1	FQEH0BT01E2E7V	1	Methylenetetrahydrofolate reductase 1	MTHR1_ARATH
970	13397	48	1	FQEH0BT01AH3MW	1	Tetrahydroprotoberberine-*N*-methyltransferase	Q108P1_PAPSO
971	73375	48	1	CL2Contig3	105	Cell wall invertase	Q9ZP42_FRAAN
972	77692	48	1	CL1Contig8953	34	Uncharacterized protein	B3LFA4_ARATH
973	60099	48	1	CL655Contig1	45	T-complex protein 1, delta subunit	Q9LV21_ARATH
974	28239	48	1	CL312Contig4	4	Uncharacterized protein	A9PHY6_POPTR
975	23549	48	1	CL674Contig3	3	Phi-1 protein	O82161_TOBAC
976	60811	48	1	CL306Contig1	41	Succinate dehydrogenase iron-sulfur subunit 2	DHSB2_ARATH
977	30597	48	2	CL1Contig4371	13	RAB1A	Q40201_LOTJA
978	44946	48	1	CL1Contig8493	5	Uncharacterized protein	A9NWV6_PICSI
979	15787	47	1	FQEH0BT01E2WNA	1	Uncharacterized protein	A2Y4I2_ORYSI
980	46949	47	1	CL2225Contig4	6	Serine/threonine-protein phosphatase	PP2A_TOBAC
981	22803	47	1	CL1Contig7508	93	Uncharacterized protein	A9NZ81_PICSI
982	72955	47	1	CL1Contig7895	173	Serine hydroxymethyltransferase	O23254_ARATH

983	18916	47	1	CL385Contig2	5	Monocopper oxidase	SKU5_ARATH
984	66615	47	2	CL505Contig1	32	Eukaryotic translation initiation factor 2 gamma	Q2QME5_ORYSJ
985	38193	47	1	CL1433Contig2	7	Rab GDP dissociation inhibitor alpha	B6TLN5_MAIZE
986	61278	47	1	CL537Contig3	39	Uncharacterized protein	Q94CF0_ARATH
987	63250	47	1	CL29Contig7	63	Predicted protein	A9U2F5_PHYPA
988	44614	47	1	CL1Contig9235	14	Initiation factor eIF4 gamma	Q2HUL1_MEDTR
989	25821	47	1	CL7178Contig1	5	2-Oxoglutarate dehydrogenase, E1 subunit	Q9ZRQ2_ARATH
990	-	47	1	FQEH0BT01EW4MQ	1	Ubiquitin-conjugating enzyme 2	Q45W76_ARAHY
991	8778	47	1	FQEH0BT01D9XPD	1	Cell division control protein 48 homolog D	CD48D_ARATH
992	20612	47	1	CL394Contig2	15	Uncharacterized protein	A9PBP7_POPTR
993	21418	47	1	CL1Contig7792	2	Glutathione *S*-transferase	Q8H9E5_CUCMA
994	16494	47	1	CL4844Contig1	2	Thioredoxin 3	Q5ZF46_PLAMJ
995	21493	47	1	CL1Contig3942	4	DEAD/DEAH box RNA helicase	Q9ZVW2_ARATH
996	13014	47	1	FQEH0BT01AGQMZ	1	Serine hydroxymethyltransferase	A9PL09_POPTM
997	9997	47	1	FQEH0BT01B23A7	1	ATP synthase gamma chain	A7BJ39_IPOTI
998	28055	47	1	CL1512Contig1	4	DEAD-box ATP-dependent RNA helicase 11	RH11_ARATH
999	27192	47	2	CL6143Contig1	6	Hydroxyphenylpyruvate reductase	Q65CJ7_SOLSC
1000	10144	47	1	FQEH0BT01BDBTB	1	Splicing factor	Q011A4_OSTTA
1001	37509	46	2	CL162Contig1	16	Growth regulator like protein	O23508_ARATH
1002	14738	46	1	FQEH0BT01BRUK1	1	Serine hydroxymethyltransferase	A3B494_ORYSJ
1003	17286	46	1	FQEH0BT01EDRIH	1	Glutamate *N*-acetyltransferase	Q3C251_CITLA
1004	30013	46	1	CL9158Contig1	4	WD-40 repeat protein	Q5VPC8_ORYSJ
