# Supplementary material for: Integration of deep transcriptome and proteome analyses reveals the components of alkaloid metabolism in opium poppy cell cultures
Source: BMC Plant Biol. 2010 Nov 18;10:252. doi: 10.1186/1471-2229-10-252 (PMC3095332; doi:10.1186/1471-2229-10-252)
Supplement: Additional file 6 — Candidate proteins identified by LC-MS/MS and potentially involved in benzylisoquinoline alkaloid metabolism in opium poppy cell cultures. [file 1471-2229-10-252-S6.PDF]

Additional File 6: List of candidate enzymes identified by LC-MS/MS and potentially involved in benzylisoquinoline alkaloid metabolism in opium poppy cell cultures.

FAD oxidoreductases

Rank	Annotation	Species	Gene Ontology/Function
27	Monodehydroascorbate reductase	*Camellia sinensis*	Oxidoreductase, FAD-binding
40	Monodehydroascorbate reductase	*Camellia sinensis*	Oxidoreductase, FAD-binding
77	Carbohydrate oxidase	*Helianthus annuus*	Oxidoreductase, FAD-binding
119	FAD linked oxidase, *N*-terminal	*Medicago truncatula*	Oxidoreductase, FAD-binding
286	Putative berberine bridge enzyme	*Arabidopsis thaliana*	Oxidoreductase, FAD-binding
324	Aldehyde oxidase	*Solanum lycopersicum*	Oxidoreductase, FAD-binding
620	Monohydroascorbate reductase(NADH)-like	*Arabidopsis thaliana*	Oxidoreductase, FAD-binding

Other oxidoreductases

Rank	Annotation	Species	Gene Ontology/Function
266	Polyphenol oxidase	*Ananas comosus*	Catechol oxidase, metal-ion binding
325	Polyphenol oxidase	*Malus domestica*	Catechol oxidase, copper-ion binding
359	NADPH:quinone oxidoreductase	*Arabidopsis thaliana*	NAD(P)H dehydrogenase
401	Polyphenol oxidase	*Taraxacum officinale*	Catechol oxidase, metal-ion binding
543	Dehydroascorbate reductase	*Malus domestica*	Glutathione dehydrogenase
549	Cytochrome P450	*Coptis japonica*	Monooxygenase, heme-binding
609	Similar to Dihydroflavonol reductase	*Arabidopsis thaliana*	Reductase, coenzyme-binding
671	Oxidoreductase 2OG-Fe(II) oxygenase	*Arabidopsis thaliana*	Oxidoreductase, Iron-binding
673	Polyphenol oxidase	*Malus domestica*	Catechol oxidase, copper-ion binding
763	Polyphenol oxidase	*Vitis vinifera*	Catechol oxidase, copper-ion binding
767	Oxidoreductase 2OG-Fe(II) oxygenase	*Arabidopsis thaliana*	Oxidoreductase, Iron-binding
797	Cytochrome P450	*Coptis japonica*	Monooxygenase, heme-binding
830	NADH dehydrogenase subunit 7	*Beta vulgaris*	Oxidoreductase, NAD or NADH binding
837	Dehydrogenase complex E1 alpha subunit	*Brassica campestris*	Pyruvate dehydrogenase (acetyl-transfering)
983	Monocopper oxidase	*Arabidopsis thaliana*	Oxidoreductase, copper-ion binding

Others

Rank	Annotation	Species	Gene Ontology/Function
639	Acetyltransferase-like protein	*Oryza sativa*	Transferase
640	Benzoquinone reductase	*Gossypium hirsutum*	2-hydroxy-1,4-benzoquinone reductase, FMN-binding
752	*N*-acetyltransferase	*Arachis hypogaea*	*N*-acetyltransferase
777	N-terminal acetyltransferase	*Arabidopsis thaliana*	Transferase
948	Tropinone reductase-like	*Arabidopsis thaliana*	Short-chain denydrogenases/reductases
